# Cell atlas of the developing human meninges reveals a dura-like nature of meningiomas

**DOI:** 10.1038/s41556-026-02074-9

**Published:** 2026-09-28

**Authors:** Elin Vinsland, Sergio Marco Salas, Ivana Kapustová, Lijuan Hu, Simone Webb, Xiaofei Li, Xiaoling He, Mats Nilsson, Muzlifah Haniffa, Roger A. Barker, Oscar Persson, David R. Raleigh, Erik Sundström, Peter Lönnerberg, Sten Linnarsson

**Affiliations:** 1 Laboratory of Molecular Neurobiology, Department of Medical Biochemistry and Biophysics, Biomedicum, Karolinska Institutet, Stockholm, Sweden; 2 Science for Life Laboratory, Department of Biochemistry and Biophysics, Stockholm University, Stockholm, Sweden; 3 Institute of Computational Biology, Computational Health Center, Helmholtz Munich, Munich, Germany; 4 Biosciences Institute, Newcastle University, Newcastle upon Tyne, UK; 5 Wellcome Sanger Institute, Hinxton, UK; 6 Division of Neurogeriatrics, Department of Neurobiology, Care Sciences and Society, Karolinska Institutet, Solna, Sweden; 7 John van Geest Centre for Brain Repair, Department of Clinical Neurosciences, Cambridge Stem Cell Institute, University of Cambridge, Cambridge, UK; 8 Department of Clinical Neuroscience, Karolinska Institute, Stockholm, Sweden; 9 Department of Neurosurgery, Karolinska University Hospital, Stockholm, Sweden; 10 Department of Radiation Oncology, Department of Neurological Surgery, Department of Pathology, University of California San Francisco, San Francisco, CA USA

**Keywords:** CNS cancer, Cell lineage

## Abstract

The vertebrate central nervous system is enveloped by the meninges, consisting of the pia, arachnoid and dura layers. The arachnoid is hypothesized to give rise to the most common primary intracranial tumours, meningiomas. However, supporting molecular evidence is lacking. There are no effective medical therapies to treat meningiomas that are resistant to local interventions, encumbered by our limited understanding of their cellular origin. Here, to advance our understanding of meningioma biology, we generated a comprehensive reference single-cell and spatial transcriptomic atlas of human fetal meninges at postconceptional weeks 5–13. We found that the meningeal layers develop concurrently, and identified an inner *CDH1*-positive dura layer expressing tight-junction genes consistent with barrier function. Comparing meningiomas with fetal meninges, we show that, transcriptionally, meningioma cells resemble dura-lineage cells, and that common meningioma driver genes were expressed in the dura lineage. This raises the hypothesis that meningiomas could originate from dura-lineage cells.

## Abstract (web summary)

Vinsland et al. provide a single-cell and spatial transcriptomics atlas of postconceptional week 5–13 human meninges and perform comparative studies with meningioma data.

## Main

The vertebrate central nervous system (CNS) is enveloped by the meninges, comprising the pia, arachnoid and dura maters. The meninges attach the brain to the skull, supply blood flow and immune protection, and provide space through which cerebrospinal fluid (CSF) flows around the brain. Several CNS developmental defects result from aberrations of the meninges, such as those caused by *FOXC1* mutations^1^. Importantly, the meninges give rise to the most common primary intracranial tumours, meningiomas. Although commonly benign or low grade, there are no effective medical therapies for higher-grade atypical or anaplastic meningiomas that are resistant to standard interventions^2^, partly due to our limited understanding of their origin.

Meningioma tumours arise from meningeal cells, but the precise cell type of origin is not fully known. John Cleland (1864)^3^ first described meningiomas as a tumour of the arachnoid granulations, which are protrusions of the arachnoid mater into the dural venous sinuses that drain CSF into venous blood. Martin Schmidt (1902)^4^ showed that meningioma cells can resemble arachnoid cap cells, which form whorls and psammoma (sand-like) bodies on the perimeter of arachnoid granulations—an observation agreed upon by the influential neurosurgeon Percival Bailey (1931)^5^. Contemporary authors generally concur with the arachnoid cap cell origin^6^. However, this origin has only been inferred on the basis of histological and morphological similarities, while molecular evidence is lacking.

In other tumours, the cell of origin has been inferred from transcriptional similarity to normal cell types, for example, based on single-cell atlases^7^. However, meningiomas have not yet been described on the basis of their similarity to cells of the different meningeal layers. While efforts to map the cellular architecture of the meninges have increased in recent years, few studies have specifically focused on meningeal fibroblasts. The first such effort performed single-cell RNA sequencing (scRNA-seq) of embryonic day (E)14.5 *Col1a1*-positive cells in mice, and shed initial light on fibroblast heterogeneity within the pia, arachnoid and dura mater^8^. This was followed by a scRNA-seq study of the human adult dura^9^, and the adult mouse and human leptomeninges (pia and arachnoid)^10,11^. Other studies incidentally included parts of the meninges in their datasets when sampling the brain^12,13^, whereas others focused their analyses on the cranium^14,15^ or immune cells^16^.

Tumours tend to hijack developmental mechanisms^7,17,18^. Therefore, understanding the molecular determinants of meningeal origin and development is key to identifying the drivers of growth and transformation of meningiomas. Despite the clinical importance of meningeal origin for meningioma biology, no comprehensive cell atlas has been published covering the developing human meninges. As a result, not much is known about how the human meninges develops either, such as the genesis and maturation of meningeal layers by fibroblasts, and in codevelopment with immune and vascular cells in the meninges.

**Fig. 1:**
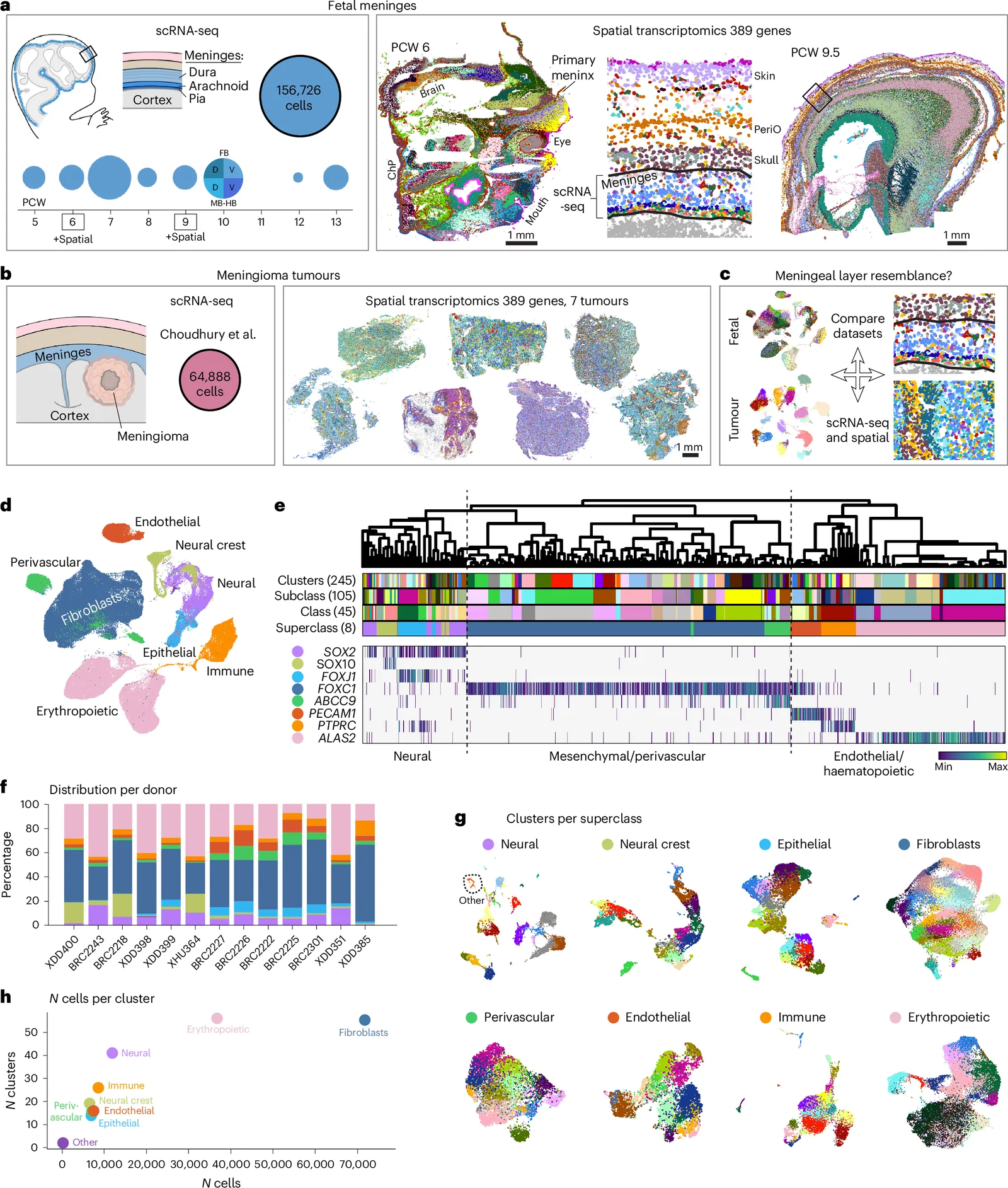
Cellular and spatial atlas of the developing human meninges. **a**, Data from fetal meninges. Left: number of cells collected from PCW 5–13 meninges for scRNA-seq (in total, 156,726 cells passing QC, *n* = 13 samples). Right: spatial transcriptomics of sagittal sections of PCW 6 and 9.5 heads (*n* = 1 each). Polygons are segmented nuclei coloured by clusters. Lines define the meninges and where scRNA-seq data were collected. **b**, Data from meningioma tumours. Left: scRNA-seq data from Choudhury et al.^19^. Right: spatial transcriptomics generated in this study (*n* = 7 tumours). **c**, Strategy to compare scRNA-seq and spatial data from fetal meninges and meningioma tumours, to study conserved developmental cell types in meningioma. **d**, UMAP embedding of scRNA-seq data from fetal meninges coloured by Superclass. **e**, scRNA-seq dendrogram with attributes from top to bottom: 245 clusters, 105 subclasses, 45 classes and 8 superclasses; coloured as in **d**, heatmap showing gene expression of neural cells (*SOX2*), neural crest (*SOX10*), ependymal cells (*FOXJ1*), meningeal fibroblasts (*FOXC1*), pericytes (*ABCC9*), endothelial cells (*PECAM1*), immune cells (*PTPRC*) and erythropoietic cells (*ALAS2*). **f**, Distribution of superclass cell types across donors. Colours as in **d** and **e**. **g**, UMAPs of each superclass coloured by clusters. **h**, Scatter plot of number of cells and number of clusters per superclass. *n* = 13 samples for **d**–**g**. Source data

Here, we therefore set out to survey the development of human meninges, from embryonic postconceptional week (PCW) 5–8 and fetal PCW 9–13 (hereafter PCW 5–13 is collectively referred to as ‘fetal’), by scRNA-seq and probe-based in situ gene expression analysis (Xenium, hereafter ‘spatial transcriptomics’; Fig. 1a). We also collected published bulk RNA-seq and scRNA-seq data for human meningiomas^19^, and generated new spatial transcriptomic data for seven such tumours (Fig. 1b). Below, we describe our cell atlas of the developing meninges, focusing on immune cells, vascular cells and, importantly, fibroblasts. We then use well-annotated fibroblast cell types and gene expression programs that are active during normal meningeal development as a blueprint to further our understanding of the cellular composition and drivers of human meningiomas (Fig. 1c).

Our work is published alongside a paper by the Sarah Teichmann group^20^ that complements our findings with a particular focus on the immune compartment of the developing meninges.

## Results

### Comprehensive cellular and spatial atlas of the developing human meninges

**Extended Data Fig. 1:**
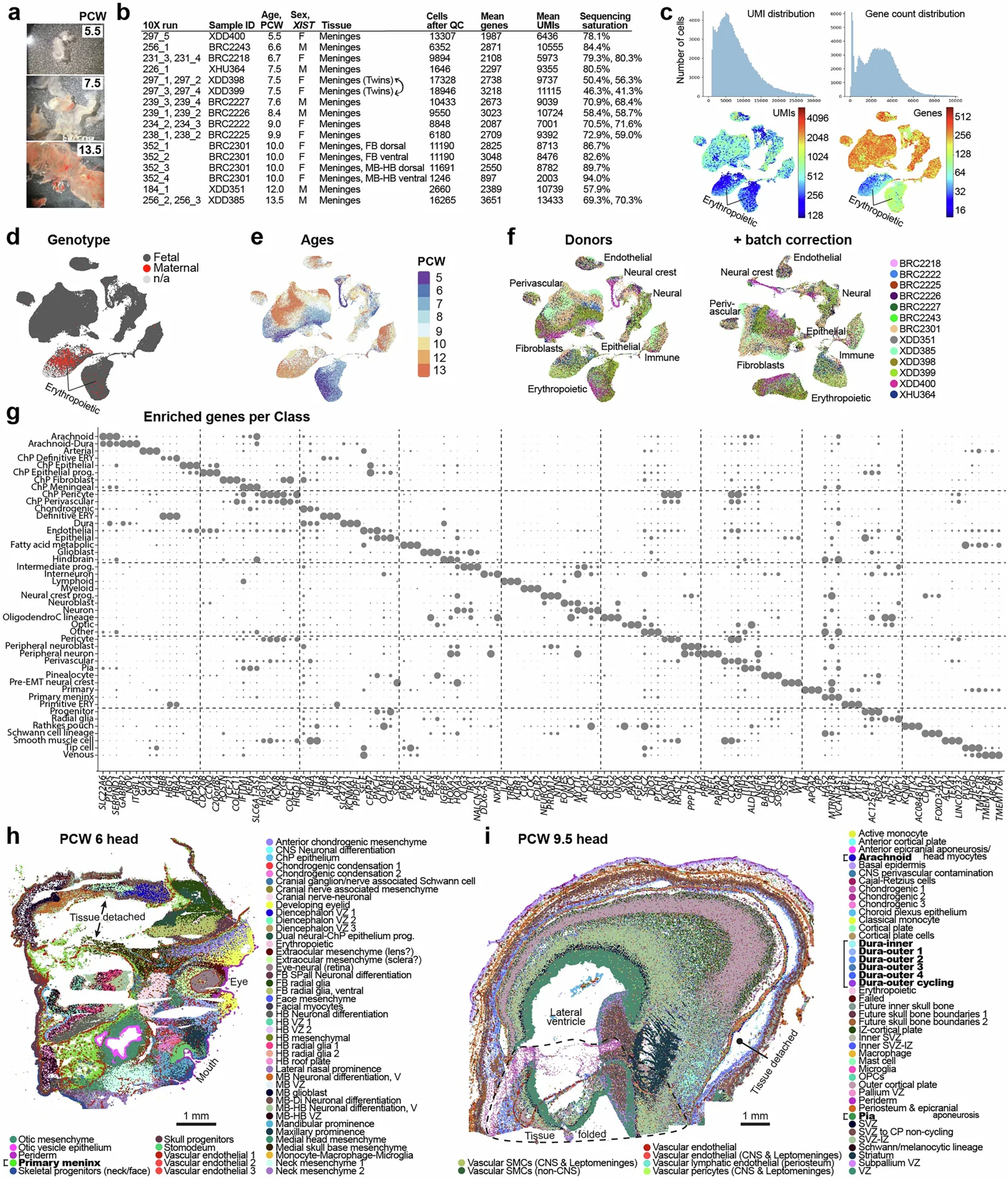
scRNA-seq QC and spatial clusters, related to Fig. 1. **a**, PCW 5.5 (n = 1), 7.5 (n = 3) and 13.5 (n = 1) meninges dissected off fetal brains. **b**, ScRNA-seq samples and sequencing information. **c**, Top: UMI distribution and gene count histograms. Bottom: UMAP embeddings of the scRNA-seq dataset, coloured by log UMIs and log genes per cell. **d**, UMAP coloured by maternal/fetal genotype, from Souporcell SNP analysis. **e**, UMAP coloured by sample ages. **f**, UMAPs without and with Harmony batch correction, coloured by donors. **g**, Dotplot of enriched genes per Class annotation. n = 13 samples for **c-g**. ChP, Choroid plexus; EMT, epithelial-mesenchymal transition; ERY, erythropoietic; OligodendroC, oligodendrocyte; prog., progenitor. **h-i**, Spatial transcriptomic clusters in sagittal sections of one PCW 6- (H) and one PCW 9.5 (I) fetal head. Brackets and bold text highlight meninges-associated clusters. Dashed lines show where tissue folded before analysis. CP, cortical plate; Di, diencephalon; HB, hindbrain; IZ, intermediate zone; MB, midbrain; OPC, oligodendrocyte progenitor cell; SMC, smooth muscle cell; SVZ, subventricular zone; V, ventral; VZ, ventricular zone. Source numerical data and code to reproduce the figure has been deposited at Zenodo under 10.5281/zenodo.21356807.

For scRNA-seq, we collected 16 human meninges samples from 13 donors across PCW 5–13. We collected the whole meninges that peeled off the brain (Extended Data Fig. 1a,b). A total of 156,726 high-quality cells were used for further analysis (Fig. 1a), and the mean unique molecular identifiers (UMIs) per sample were 8,842. As expected, anucleated erythropoietic cells showed the lowest gene and UMI counts (Extended Data Fig. 1c); excluding those cells, the mean UMI count was 10,249 (Supplementary Table 1). An analysis of the distribution of maternal and paternal SNP alleles^21^ (Methods) indicated that all cells were fetal except some erythrocytes (Extended Data Fig. 1d). We found that the data were well integrated across samples even in the absence of batch correction, which we therefore omitted to avoid suppressing true biological variation (Extended Data Fig. 1e,f).

We generated a total of 245 clusters, annotated to four levels of detail: cell type (*n* = 115), subclass (*n* = 105), class (*n* = 45) and superclass (*n* = 8). We computed metadata such as cell cycle score, fetal age and enriched genes for each cluster (Supplementary Table 2). The largest superclass consisted of *FOXC1*^+^ fibroblasts, which made up half the dataset, in addition to *PECAM1*^+^ endothelial, *ABCC9*^+^ perivascular, *PTPRC*^+^ immune and *ALAS2*^+^ erythropoietic cells in the meninges, across all donors. We also acquired interacting *SOX2*^+^ neural, *SOX10*^+^ neural crest and *FOXJ1*^+^ epithelial (choroid plexus, ChP) cells (Fig. 1d–h). Due to the diversity of our dataset, we were able to generate highly specific markers for many cell types of the meninges (Extended Data Fig. 1g).

We used spatial transcriptomics to validate and locate cell types identified from using scRNA-seq data. To keep the meninges anatomically intact, we cryosectioned whole heads at PCW 6 and PCW 9.5. Two mediolateral sagittal sections from PCW 6 and one lateral sagittal section from PCW 9.5 were analysed, with a custom probe-set targeting 389 genes (Supplementary Table 3) selected on the basis of the fetal scRNA-seq data. We generated 53 and 50 clusters respectively, after single-nucleus segmentation at the two ages (Extended Data Fig. 1h,i and Supplementary Table 4). At PCW 6, we found diverse populations of craniofacial mesenchyme, neural crest-derived tissues, progenitors and differentiating neural cells of the CNS, ChP and vascular and immune cells. Importantly, we identified the earliest described layer of the meninges; the primary meninx (Extended Data Fig. 1h). At PCW 9.5, clustering highlighted a remarkably complete stack of layers spanning the developing cortical layers from the ventricular zone to Cajal–Retzius cells in the cortical plate, to fibroblasts of the meninges, future skull, periosteum and skin (more in Fig. 4). We also noted scattered populations of various immune cells, Schwann cells (melanocytic lineage) and vascular cells, among others (Extended Data Fig. 1i).

In the following sections, we analysed each superclass of meningeal cells—immune, vascular and fibroblasts—focusing on their heterogeneity and maturation during meningeal development.

### Fetal immune heterogeneity and B-lineage cells from PCW 5

**Fig. 2:**
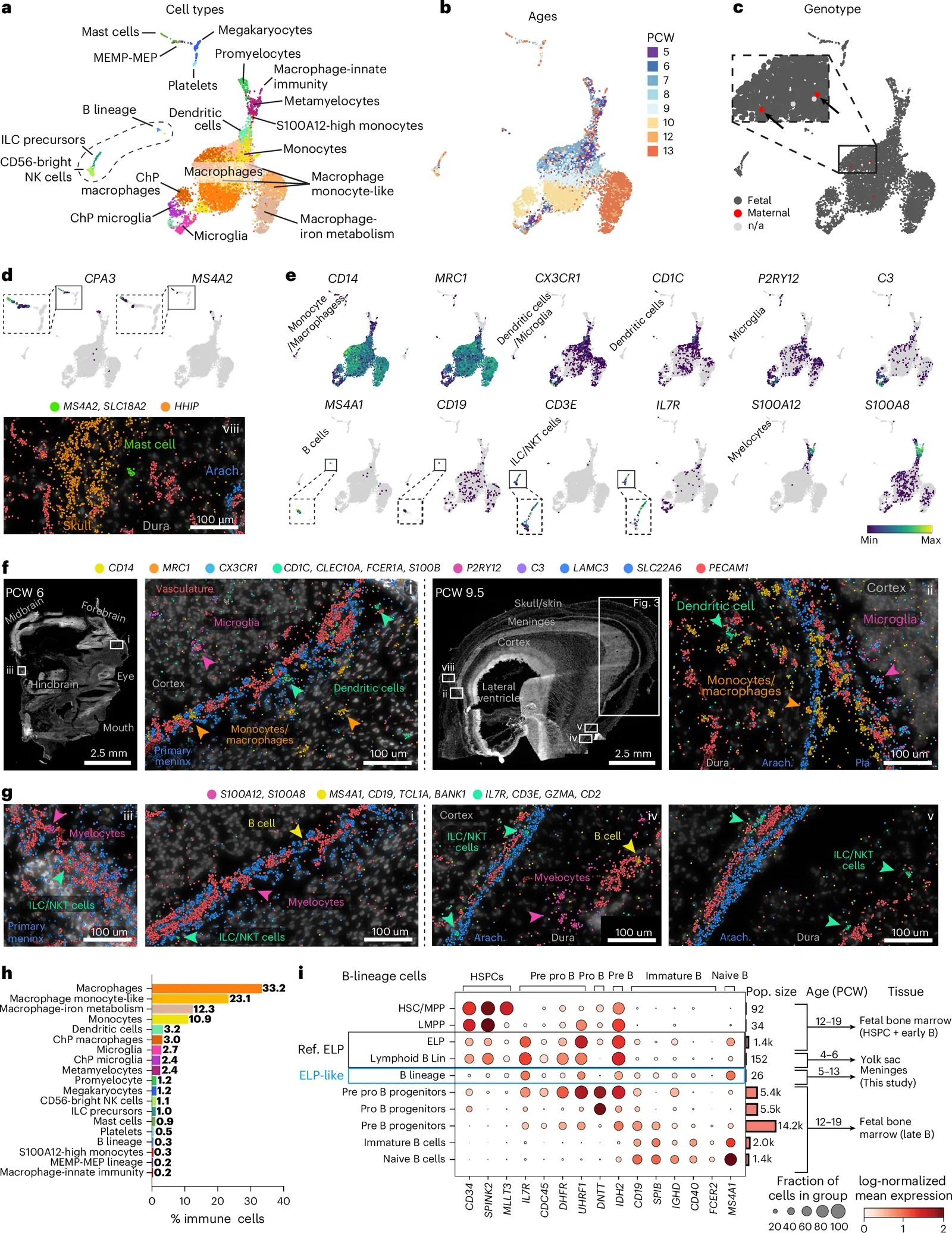
Immune cell diversity in the developing meninges. **a**, UMAP coloured by clusters of annotated immune cells. MEMP, megakaryocyte erythroid mast cell progenitor; MEP, megakaryocyte erythroid progenitor. **b**, UMAP coloured by sample age. **c**, UMAP coloured by maternal/fetal genotype, by maternal/paternal SNP analysis (Methods). The inset highlights two maternal immune cells (arrows). n/a, genotype not called. **d**, UMAPs coloured by gene expression on a grey background of all cells, and spatial transcriptomics at PCW 9.5, of mast cell markers. **e**, UMAPs coloured by gene expression of immune cell markers. **f**, Spatial transcriptomics of PCW 6 (*n* = 1) and 9.5 (*n* = 1) fetal heads, showing RNA molecules on DAPI. Marker genes for microglia, monocytes/macrophages and dendritic cells are shown, coloured as in **a**. **g**, The same as **f** but for ILC/natural killer T (NKT) cells, B cells and the myelocytic lineage. **h**, Percentage of immune cell types, coloured as in **a**. **i**, Dot plot of scRNA-seq gene expression of B-lineage markers across developmental tissues (fetal bone marrow as B cell ref. ^24^ versus yolk sac^23^, and meninges (these data)). The size of the dot represents the percentage of cells within a group that express a given gene, and the colour of the dot indicates ln(*x* + 1) mean gene expression within the group, where *x* equal counts normalized to 1 × 10^4^ per cell. ELP, early lymphoid cell; HSCs, haematopoietic stem cell; HSPCs, haematopoietic stem and progenitor cells. *n* = 13 meninges samples for **a**–**e**, **h** and **i**. Source data

**Extended Data Fig. 2:**
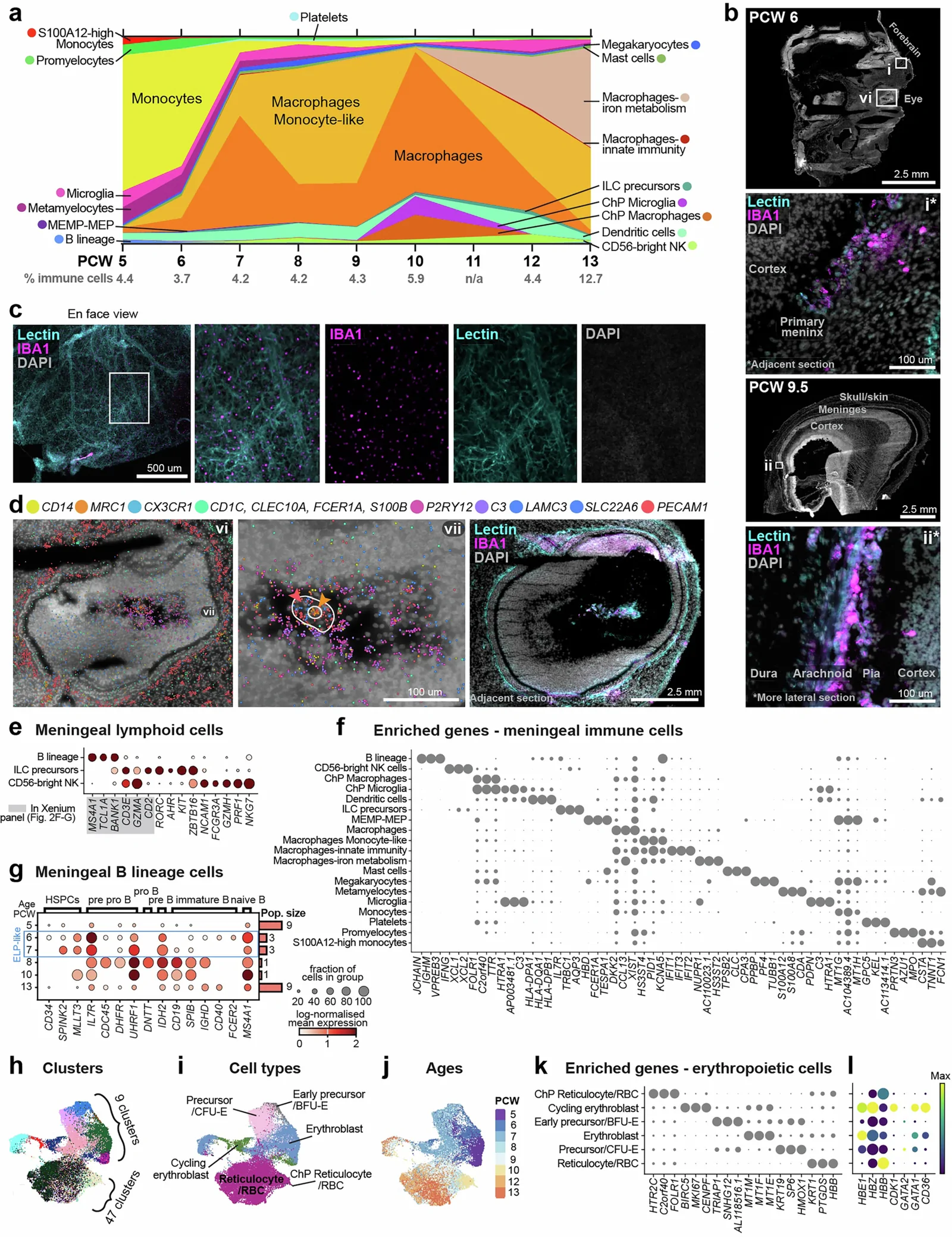
Macrophages/microglia, lymphoid, and erythropoietic cells, related to Fig. 2. **a**, Proportion of immune cell types by age, for all n = 13 samples. In grey: proportion of immune cells by age compared to all cells in the meninges. **b**, IBA1 Immunostaining (macrophages and microglia), Lectin dye (vasculature), and DAPI (nuclei) in sagittal sections adjacent to sections used for spatial transcriptomics (n = 1). **c**, As **b** but PCW 9.5 floating meninges (n = 3). **d**, PCW 6 eye, with spatial transcriptomic data showing dots representing RNA molecules of markers for monocytes/macrophages, microglia, and dendritic cells, and IBA1 immunostaining (n = 1). **e**, Dot plot showing the expression of lymphoid markers in a subset of meningeal lymphoid cells. Grey box indicates genes also included in the spatial transcriptomics (Xenium in situ) probe panel. **f**, Enriched genes for fetal meningeal immune cells for all n = 13 samples. **g**, Dot plot of scRNAseq gene expression of B-lineage markers in developmental meninges across time (from 5-13 PCW). The size of the dot represents the % of cells within a group that express a given gene, and the colour of the dot indicates ln(x + 1) mean gene expression within the group, where x equal counts normalised to 1e4 per cell. ELP, early lymphoid cell. **h**, UMAP of erythropoietic cells coloured by clusters (n = 13 samples for **h-l**). **i**, UMAP of erythropoietic cells coloured by cell type annotation. **j**, UMAP of erythropoietic cells coloured by sample ages. **k**, Enriched genes per erythropoietic cell annotation. **l**, Dot plot showing expression of yolk sac-derived primitive (*HBE1/HBZ*) and definitive (*HBB*) markers, and classical genes involved in erythropoiesis. Source numerical data is in Source_Data_EDFig2.xlsx, and code to reproduce the figure has been deposited at Zenodo under 10.5281/zenodo.21356807. Source data

The fetal meninges harboured a surprising heterogeneity of immune cells across PCW 5–13, even at the earliest stages. We identified 19 cell types of myeloid and lymphoid origin (Fig. 2a,b and Extended Data Fig. 2a). Except for three cells, all immune cells were of fetal origin (Fig. 2c). Furthermore, spatial transcriptomics validated and located those immune cells in the primary meninx at PCW 6, and leptomeninges and dura at PCW 9.5 (Fig. 2d–g).

Macrophages were the most abundant (Fig. 2h), with plentiful IBA1^+^ cells concentrated around the primary meninx/leptomeninges at PCW 6 and 9.5 (Extended Data Fig. 2b). IBA1 staining of meninges revealed a uniform sparse tiling of macrophages in en face floating sections (Extended Data Fig. 2c), consistent with repelling mechanisms controlling their location. The leptomeninges defined an anatomical barrier where microglia (*CX3CR1*, *P2RY12* and *C3*) always resided on the brain side, while monocytes/macrophages (*CD14* and *MRC1*) resided in the leptomeninges and the rest of the head, already from PCW 6 (Fig. 2f). Microglia were also concentrated inside the developing eye, while monocytes/ macrophages resided outside, except one macrophage inside a ring of endothelial cells at the very centre (Extended Data Fig. 2d).

There is growing evidence for a B lymphoid cell presence in early human development. Recent investigations defined *CD34*^+^*IL7R*^+^*CD19*^−^ early lymphoid progenitors (ELP) in human fetal liver as early as PCW 6^22^, and the yolk sac ‘lymphoid B lineage’ from PCW 4–6^23^. These data raise the intriguing possibility that adaptive lymphoid cells are generated and established in peripheral organs as early as PCW 6. However, this has not been determined yet in the embryonic head and meninges. B-lineage cells made up 0.3% of immune cells in the meninges (Fig. 2h) and were clearly distinct from innate lymphoid subtypes (innate lymphoid cell precursors and CD56-bright natural killer cells) (Extended Data Fig. 2e,f). Our scRNA-seq data confirmed that *CD34*^+^*IL7R*^+^*CD1*9-low ELP-like B-lineage cells were transcriptionally similar to second trimester fetal bone marrow ELPs^24^, where B cells are well described to differentiate and diversify (Fig. 2i). We confirmed ELP presence in embryonic meninges as early as PCW 5, and up until about PCW 7, after which point (PCW 8-13) the meningeal B lineage appeared to differentiate and acquire more mature markers for pre pro-, pro-, pre-, and immature B cells (Extended Data Fig. 2g).

These data position the meninges as a contributor to the first- and second-trimester differentiation and diversification of human B cells. Furthermore, we demonstrate B cell presence in the embryonic meninges 1 week earlier in gestation than previously reported for liver tissue.

### Fetal meningeal vasculature and ChP

**Fig. 3:**
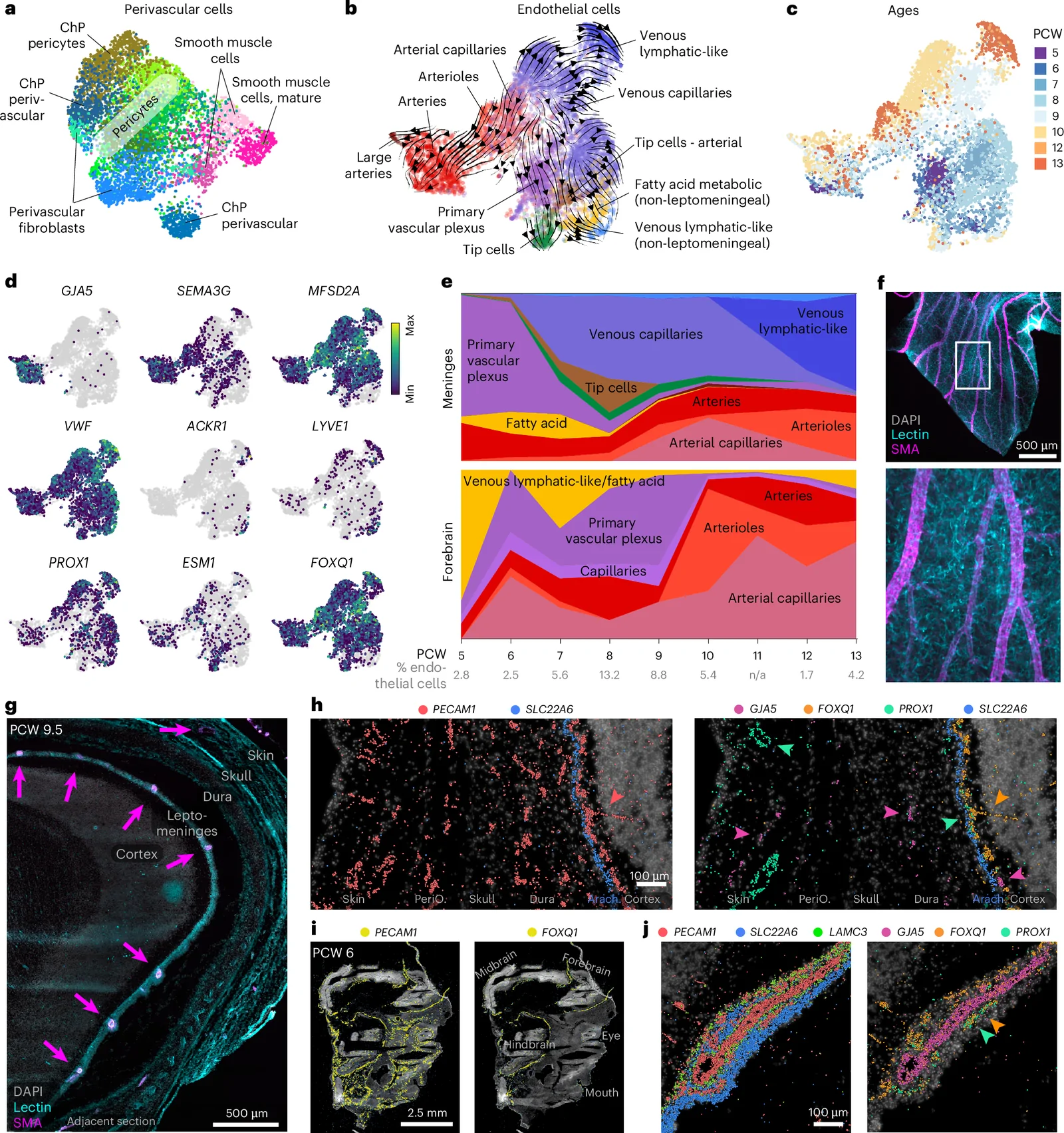
Vasculogenesis in the meninges and brain. **a**, UMAP of perivascular cells (*n* = 13 samples). **b**, UMAP of endothelial cells (*n* = 13 samples), coloured by arteriovenous zonation. Arrows show RNA velocities. **c**, UMAP of endothelial cells coloured by sample ages. **d**, Gene expression of arteriovenous zonation markers, as well as lymphatic and tip cells. **e**, Proportion of endothelial cell types by age, in the meninges (*n* = 13) and forebrain^13^ (*n* = 14). **f**, Lectin dye (vasculature), DAPI (nuclei) and smooth muscle actin (SMA) immunostaining in a PCW 9.5 floating meninges (*n* = 3). **g**, The same as **f** but in a sagittal section (red arrows point to arteries, *n* = 1). **h**, Spatial transcriptomics of all endothelial cells (PECAM1), arteries (GJA5 and pink arrows), venous lymphatic-like cells (PROX1 and mint-green arrows), leptomeninges and brain-specific endothelial cells (FOXQ1 and orange arrows) and arachnoid (SLC22A6) in a PCW 9.5 head. Coloured dots represent RNA molecules. **i**, Spatial transcriptomics of PECAM1 and FOXQ1 in a PCW 6 head. **j**, The same as **h** but with pia (LAMC3), around an artery. Source data

The fetal meninges contained perivascular cells such as pericytes, smooth muscle cells, and perivascular fibroblasts (Fig. 3a), as well as vascular endothelial cells (Fig. 3b,c). The endothelial cells expressed stereotypical genes involved in arteriovenous zonation (Fig. 3d,e), as previously described in mouse and human^25–28^, and also contained *COL15A1*^+^*ESM1*^+^ tip cells (Fig. 3d). RNA velocity (scVelo; Fig. 3b) and analysis of endothelial cluster composition over time (Fig. 3e) showed that PCW 5 was dominated by a primary vascular plexus giving rise to tip cells penetrating the brain. This was followed by the appearance of arterial and venous capillaries around PCW 6–9, giving rise to larger arterioles and venous lymphatic-like cells respectively at PCW 12–13. However, the forebrain lacked venous capillaries from PCW 5 to 13 (Fig. 3e), indicating that this vascular specification occurred earlier in the meninges. Immunostaining of smooth muscle actin (*ACTA2* gene) showed that larger arteries existed in the leptomeninges, dura and skin at PCW 9.5 (Fig. 3f,g).

Most endothelial cells in the meningeal scRNA-seq dataset expressed the transcription factor *FOXQ1* (Fig. 3d), which controls the differentiation of the blood–brain barrier endothelial cells in mice^29^. Spatial transcriptomics showed that all endothelial cells in the head expressed *PECAM1*, while *FOXQ1* was uniquely expressed by endothelial cells below the arachnoid and extending into the brain (Fig. 3h) as early as PCW 6 (Fig. 3i).

The cellular ontogeny of meningeal lymphatic vessels is still unclear^30^. We found that *ACKR1*^+^*LYVE1*^+^ venous cells expressed the key lymphatic transcription factor *PROX1*^31–33^ (Fig. 3d). Spatially, *PROX1*^+^*FOXQ1*^+^ endothelial cells were present below the arachnoid, and *PROX1*^+^*FOXQ1*^−^ endothelial cells in the developing skin at PCW 9.5 (Fig. 3h,j). These observations suggest that (1) meningeal lymphatic cells were mostly derived from the venous endothelium, and (2) subarachnoid lymphangiogenesis may have started in early fetal development, surprisingly so, because meningeal lymphatics develop postnatally in mice^34^.

**Extended Data Fig. 3:**
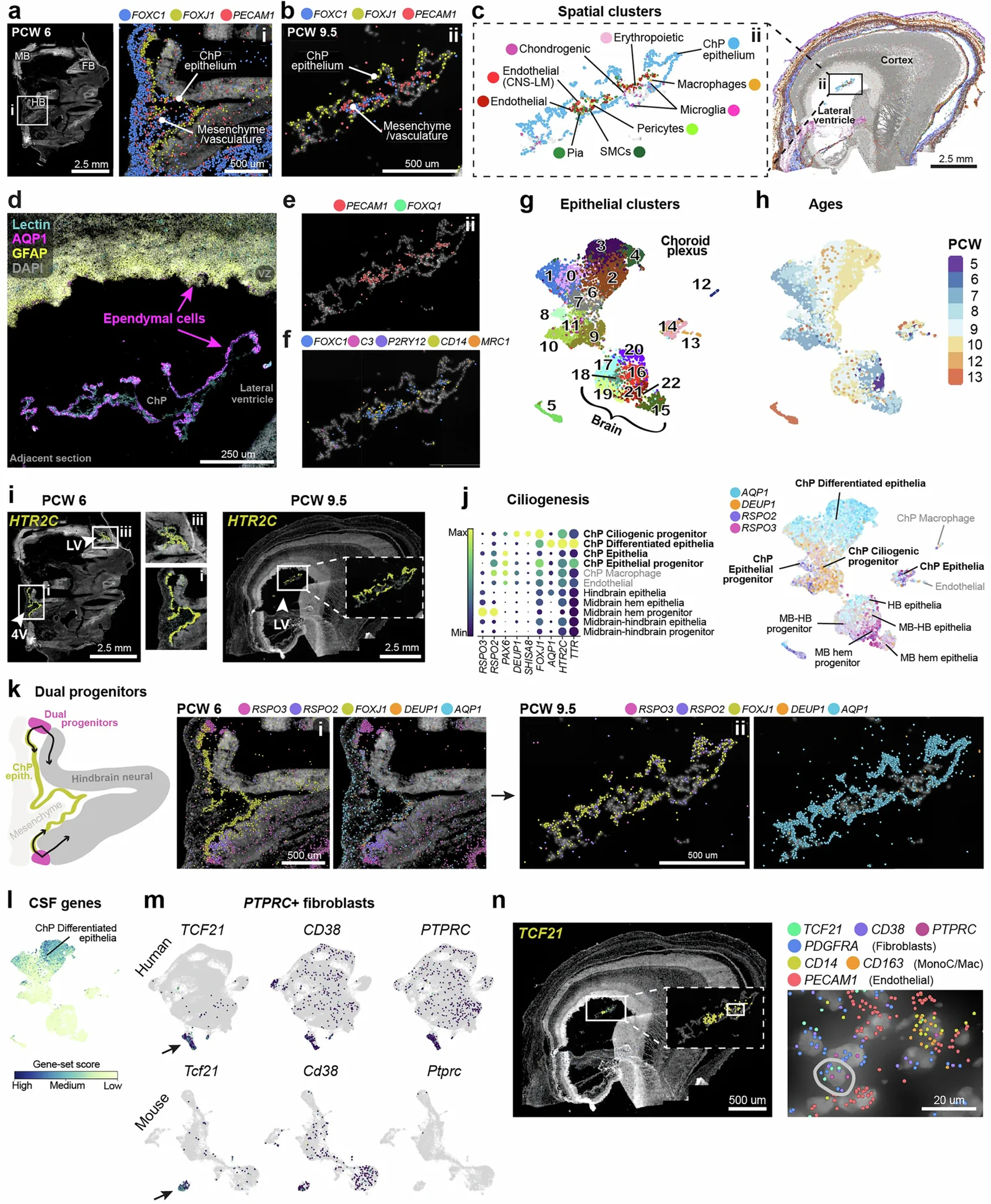
Fetal choroid plexus development, related to Fig. 3. **a**, Spatial transcriptomic data showing *FOXC1*+ mesenchymal/meningeal progenitors, endothelial cells (*PECAM1*), and choroid plexus (ChP) ependymal cells (*FOXJ1*) at PCW 6 (n = 1). FB, forebrain; MB, midbrain; HB, hindbrain. **b**, Same as **a** but at PCW 9.5 (n = 1). **c**, Annotated spatial clusters of the lateral ventricle ChP at PCW 9.5. **d**, Lectin dye (vasculature), DAPI (nuclei) and Aquaporin 1 (AQP1) immunostaining (n = 1). **e**, Spatial transcriptomic data showing *PECAM1* and *FOXQ1* RNA molecules in the ChP stroma. **f**, Same as **e** but *CD14*/*MRC1*+ monocytes/macrophages, *FOXC1* + ChP meningeal stroma, and *C3/*P2RY12+ microglia. **g**, scRNA-seq UMAP of ChP and brain epithelial cells, coloured by clusters (from n = 13 samples). **h**, UMAP of epithelial cells coloured by sample ages. **i**, Spatial transcriptomics showing *HTR2C* expression (n = 2). **j**, Dot plot showing the expression of genes involved in epithelial ciliogenesis (left), per annotated cell type on UMAP (right) (n = 13). **k**, Schematic and spatial validation of *RSPO2/3*+ dual neuro-epithelial progenitors, and ciliogenesis markers, at PCW 6 and 9.5. **l**, UMAP of epithelial cells, coloured by a CSF gene-set score (Methods) (n = 13 samples). **m**, UMAPs of human fetal (this study, n = 13)) and mouse embryonic (n = 42)^44^ meningeal fibroblasts coloured by expression of *TCF21/Tcf21*, *PTPRC/Ptprc*, and *CD38/Cd38* on a grey background of all cells. **n**, Spatial validation of *TCF21* + *PTPRC* + *CD38*+ fibroblasts in the PCW 9.5 ChP (n = 1). Source numerical data and code to reproduce the figure has been deposited at Zenodo under 10.5281/zenodo.21356807.

CSF is generated by filtration of blood at the ChP, filling the brain ventricles and subarachnoid spaces. We characterized the ChP with its epithelial cells, stroma, vascular and immune cells, and its inside-out anatomy in relation to the brain and meninges (Extended Data Fig. 3a–e). The ChP epithelium develops by differentiation from adjacent neuroepithelium (future multiciliated ependymal cells). Our scRNA-seq data comprised 22 clusters of brain and ChP epithelial cells from PCW 5 to 13 (Extended Data Fig. 3g,h) and identified *HTR2C* as a unique ChP epithelial marker (Extended Data Fig. 3i). At the junctions between future ChP epithelium and surrounding neuroepithelium, we identified similar dual neuro-epithelial progenitors (*RSPO2* and *RSPO3*^+^) and ciliogenesis programs (*DEUP1* (mouse *Ccdc67*)*, SHISA8* and *AQP1*) as previously described in mice^35^ (Extended Data Fig. 3j,k). Interestingly, by creating a gene-set score based on adult CSF proteomics^36,37^ (Supplementary Table 5), we observed that CSF genes were already expressed in fetal ChP epithelial cells (Extended Data Fig. 3l).

We also identified a fibroblast type present only in the ChP, which uniquely expressed *TCF21* (Extended Data Fig. 3m,n). Both scRNA-seq and spatial transcriptomics showed that they also expressed the immune-related genes *PTPRC* (CD45, pan-leukocyte marker), and *CD38*. However, mouse *Tcf21*^+^*Cd38*^+^ fibroblasts did not express *Ptprc* (Extended Data Fig. 3m).

Our findings demonstrate that human ChP development uses mechanisms that are largely conserved from rodents, but with human-specific features that suggest an evolved role for ChP fibroblasts in immune surveillance at the blood–CSF border.

### Fibroblast layer development in the fetal meninges and head

Fibroblasts are the principal cells of connective tissues. However, a comprehensive molecular analysis of human fetal head fibroblasts and the transcriptional cell-type composition of newly forming meningeal layers remain to be elucidated.

#### Fibroblast heterogeneity and organization

**Fig. 4:**
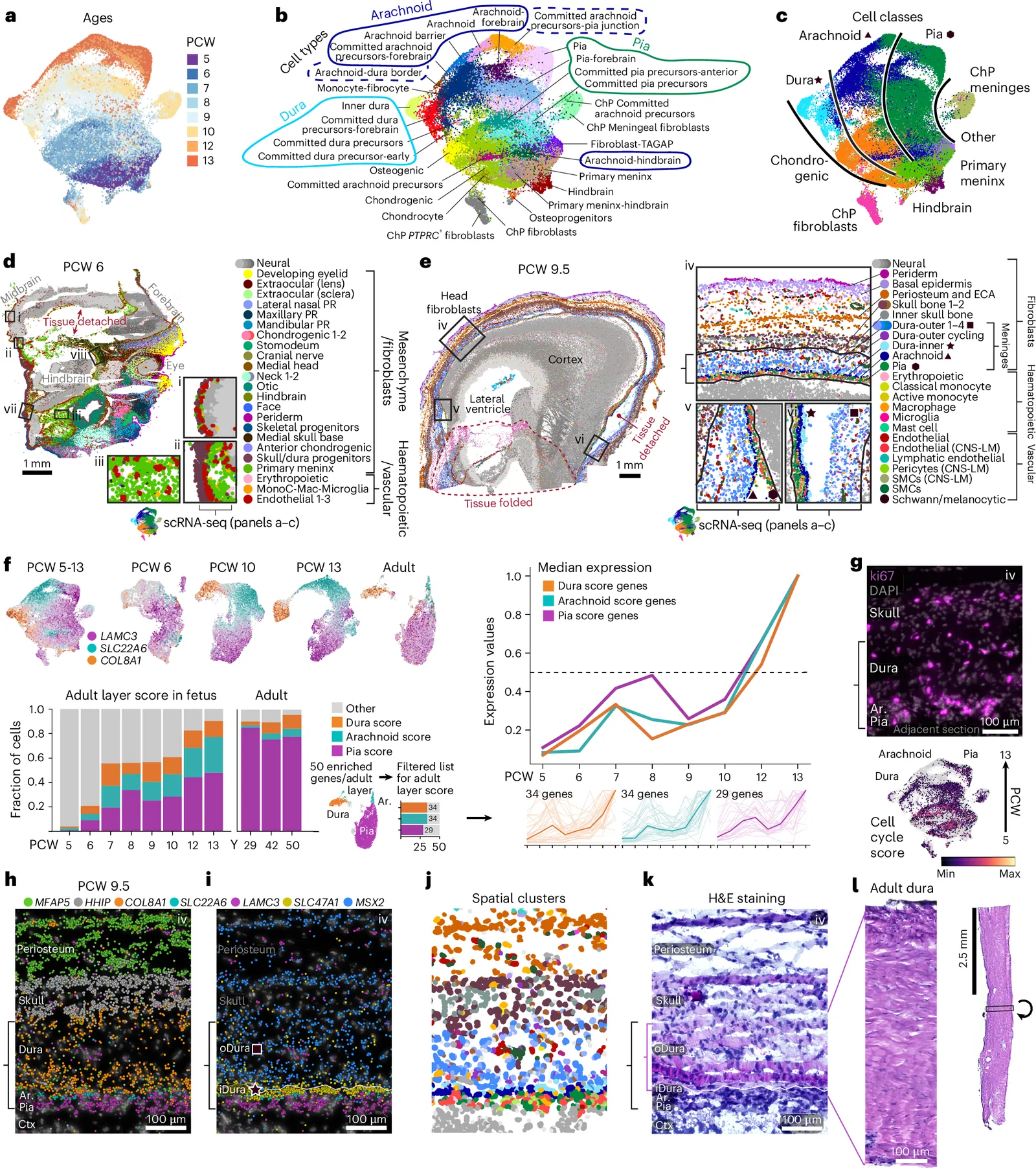
Fibroblast layer development in human fetal heads. **a**, UMAP of fetal meningeal fibroblasts coloured by sample age (*n* = 13 samples). **b**, UMAP coloured by annotated cell types. **c**, UMAP coloured by class. Black lines separate putative pia, arachnoid and dura lineages. **d**, Sagittal section of a PCW 6 head coloured by spatial clusters (*n* = 1). Three insets show the primary meninx, and where scRNA-seq was sampled. The colour for the primary meninx matches the scRNA-seq class ‘Primary meninx’ in **c**. Mac, macrophage; MonoC, monocyte; PR, prominence. **e**, The same as **d** but at PCW 9.5 around the telencephalon (*n* = 1). Insets show the meningeal clusters, and where scRNA-seq was sampled. Colours for meningeal layers match those in **c**. ECA, epicranial aponeurosis; LM, leptomeninges; SMC, smooth muscle cells. **f**, Analysis of meningeal layer maturation. Top left: UMAP of all fibroblasts (*n* = 13), subsets of PCW 6 (*n* = 2), 10 (*n* = 2) and 13 (*n* = 1) (this study) and adult^12^ (*n* = 3) fibroblasts. Cells are coloured by their expression of the gene they express most highly among *LAMC3* (pia), *SLC22A6* (arachnoid) and *COL8A1* (dura). Bottom left: stacked bar chart showing the fraction of fetal and adult fibroblasts expressing a list of enriched genes from adult pia, arachnoid, and dura (Methods). Y, years. Right: median expression of the enriched gene lists over developmental timepoints (*n* = 13). **g**, Top: immunohistochemistry of ki67 and DAPI in a PCW 9.5 head (*n* = 1). Inset region ‘iv’ as in **e** but on an adjacent section. Bottom: scRNA-seq UMAP of fibroblasts coloured by cell cycle score. **h**, Spatial transcriptomics showing layer-specific markers. Brackets indicate the meninges. **i**, The same as **h** but *SLC47A1* (inner dura) and *MSX2* (outer dura, skull and periosteum). **j**, The same region as **h** and **i** but showing spatial clusters. **k**, H&E staining (*n* = 1). Black brackets show the meninges and pink brackets the dura. **l**, H&E staining of adult human dura (*n* = 4). Source data

**Extended Data Fig. 4:**
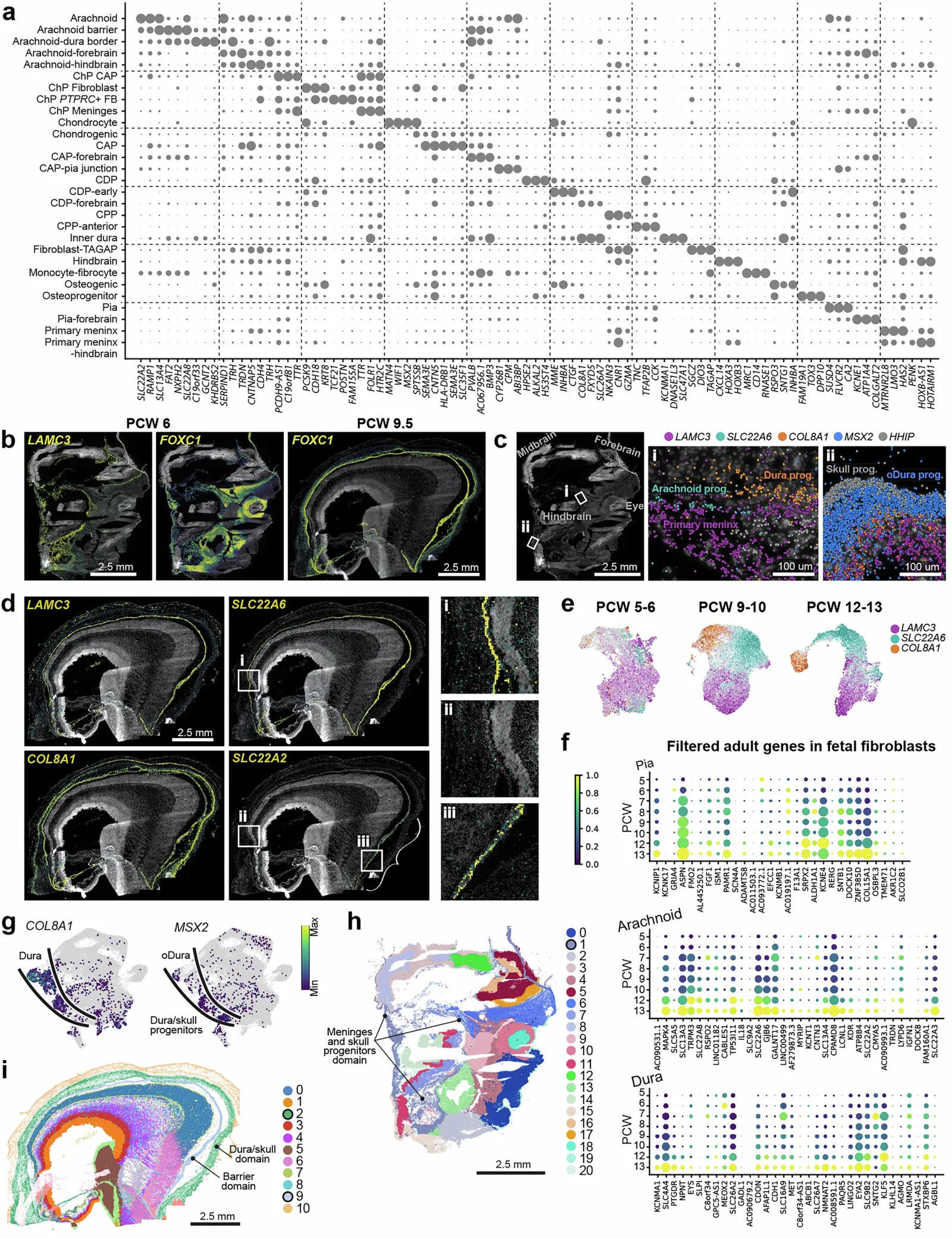
Meningeal layer marker dynamics, related to Fig. 4. **a**, Dotplot of enriched genes per CellType annotation (n = 13 samples). CAP, Committed arachnoid precursor; CDP, Committed dura precursor; ChP, Choroid plexus; CPP, Committed pia precursor. **b**, Spatial transcriptomics showing *LAMC3* and *FOXC1* expression at PCW 6, and *FOXC1* at 9.5. n = 1 for PCW 6 and 9.5 in **b-d**. **c**, Spatial transcriptomics of identified layer markers *LAMC3* (pia), *SLC22A6* (arachnoid), *COL8A1* (dura), *MSX2* (outer dura, skull and periosteum), and *HHIP* (skull) at PCW 6. **d**, Spatial transcriptomics showing *LAMC3, SLC22A6, COL8A1*, and *SLC22A2* expression at PCW 9.5. **e**, UMAPs of subsets of pooled PCW 5-6 (n = 3), 9-10 (n = 3), and 12-13 (n = 2) fibroblasts. Cells are coloured by their expression of the gene they express most highly among *LAMC3* (pia), *SLC22A6* (arachnoid), *COL8A1* (dura). **f**, Dotplot showing the expression of enriched genes from adult pia, arachnoid, and dura (Fig. 4f), in fetal fibroblasts over developmental timepoints (n = 13). **g**, UMAP showing the expression of *COL8A1* (dura) and *MSX2* (outer dura) in fetal meningeal fibroblasts. **h**, Banksy domain analysis of PCW 6 spatial transcriptomic data (n = 1). Spatial map coloured by domains. **i**, Same as **h** but for PCW 9.5 (n = 1). Source numerical data and code to reproduce the figure has been deposited at Zenodo under 10.5281/zenodo.21356807.

Our scRNA-seq analysis across the PCW 5–13 meninges yielded 71,657 fibroblasts. From this, we generated 55 clusters that we annotated as 29 cell types with distinctly enriched genes (Fig. 4a,b, Extended Data Fig. 4a and Supplementary Table 2). Twenty of these cell types belonged to the three meningeal layers; the pia, arachnoid and dura mater, their precursors, and the early primary meninx. We also captured other interesting fibroblasts, such as a fibroblast expressing *TAGAP* (T cell activating protein), osteogenic fibroblasts (*PTHLH*), chondrocytes (*MATN4*), hindbrain (*HOXA3)* fibroblasts and the previously mentioned *PTPRC*^+^ fibroblast unique to the ChP (Fig. 4b,c).

Next, we charted the distribution of fibroblast cell types in situ, in whole heads at PCW 6 and PCW 9.5 (Fig. 4d,e). At PCW 6, early craniofacial mesenchyme was found in well-defined spatial domains such as the mesenchyme of extraocular (eyes), maxillary/mandibular (jaws), otic (future ears) and cranial nerve-associated tissues (Fig. 4d). We also identified a spatial cluster representing the earliest meninges described in the literature, the primary meninx, from which we had also obtained scRNA-seq data (Fig. 4a–c). The primary meninx was a thin layer surrounding the entire brain together with *FOXQ1*^+^ endothelial cells (Fig. 4d, insets i–ii, and Fig. 3i), except around the lower ventral hindbrain where a pool of primary meninx, endothelial, immune and erythropoietic cells were mixed together (Fig. 4d, inset iii). We found that *LAMC3* was uniquely expressed by the primary meninx (Extended Data Fig. 4b and Supplementary Table 4). By contrast, the meningeal marker *FOXC1* was expressed broadly in craniofacial fibroblasts at PCW 6, and only later became specific to the meninges (Extended Data Fig. 4b).

At PCW 9.5, fetal head fibroblasts surrounding the telencephalon were already stratified into well-defined layers stacked on top of each other (Fig. 4e, inset iv). Clustering of the spatial data allowed us to identify fibroblasts of the skin (periderm and basal epidermis), future skull and periosteum, and the meningeal layers. Other cell types were also present within these fibroblast layers, such as the neural crest-derived Schwann-melanocytic lineage between the periosteum and basal epidermis, lymphatic endothelial cells and pools of erythropoietic cells in the outer periosteum, and classical monocytes mostly in the outer dura. The meninges were divided into eight spatial clusters: five of the outer dura, one inner dura layer, the arachnoid and the pia.

#### The meningeal layers develop concurrently

Next, we focused on meningeal layer formation over time (Fig. 4f–h). Using the scRNA-seq and spatial transcriptomic data, we identified and validated unique markers distinguishing the pia (*LAMC3*), arachnoid (*SLC22A6*) and dura (*COL8A1*), from PCW 5–13 and in adults (Fig. 4f,h and Extended Data Fig. 4a). *LAMC3*, which was expressed in the primary meninx at PCW 6, was specifically expressed in the pia in later ages. This indicated that the first meningeal fibroblasts to surround the brain are pia precursors, and/or that the pia develops from the primary meninx. Arachnoid and dura precursors were also present at PCW 5–6, although the emerging spatial organization of meningeal layers was found only around parts of the hindbrain in these sections (Extended Data Fig. 4c). At PCW 9.5, precursors of all three layers covered the entire cortex, and *SLC22A2*, which was expressed in more mature arachnoid populations, appeared first in the ventral telencephalon (Extended Data Fig. 4d).

We wondered whether the meningeal layers develop as separate lineages, and if therefore intermediate lineage-specific precursor cell types could be identified, as in haematopoiesis. However, examining the expression of the layer-specific markers *LAMC3*, *SLC22A6* and *COL8A1* in subsets of cells from PCW 6, 10 and 13, instead revealed gene expression gradients and a continuum of pia, arachnoid and dura cells at each age (Fig. 4f, top left). The same was true when pooling samples at PCW 5–6, 9–10 and 12–13, ruling out batch effects (Extended Data Fig. 4e). While this does not account for the spatiotemporal aspects of layer maturation observed in mice, it indicated that the meninges mature as a continuum of cell states, segregating into distinct layer-specific types only at developmental timepoints beyond PCW 13.

To further support this parallel model of meningeal development, we created a maturation score based on 50 enriched genes from each adult meningeal layer (Methods) (Fig. 4f, bottom left) and examined the temporal emergence of each layer as measured by this score. The analysis revealed that (1) genes expressed by the adult meninges were expressed in the first trimester, (2) their expression increased over PCW 5-13, demonstrating a gradual maturation of the meningeal layers, and (3) the pia, arachnoid and dura mature concurrently (Fig. 4f bottom left and right, and Extended Data Fig. 4f). The latter was also supported by the observation of cycling populations in each layer (Fig. 4g). Taken together, these results contradict a branched lineage model as seen in neurogenesis or haematopoiesis. Instead, they support a model where the meningeal layers are formed concurrently, by a gradual refinement of cell states that only segregate into truly distinct layer identities at later developmental timepoints. This resembles how patterning by morphogenetic gradients and cell–cell interactions influence cell fate and layer stratification^38,39^.

#### Identification of an inner and outer dura layer

Next, we investigated the dura in more detail (Fig. 4h–l). Our spatial transcriptomic data identified separate outer and inner layers of the dura (Fig. 4e). While both expressed *COL8A1*, the known chondrogenic marker *COL2A1*^14^ and dura marker *FXYD5*^8^, the inner layer was distinguished by high *SLC47A1* expression (previously attributed to the dura border^10^). The outer dura was distinguished by expression of *MSX2*, which was also expressed in osteogenic tissues (Fig. 4h–j and Extended Data Fig. 4g). We identified a spatial cluster representing their potential common progenitor, which joined the outside of the primary meninx to surround the brain, and expressed *MSX2*, *HHIP* and *COL2A1* (Fig. 4d ‘Skull/dura progenitors’, Extended Data Fig. 4c and Supplementary Table 4). This suggests that, from PCW 5 to 6, this lineage, together with the primary meninx, give rise to all the meningeal layers. This observation, together with the similarity between outer dura and skull progenitors, was also supported by Banksy spatial domain analysis (Methods; Extended Data Fig. 4h,i). Surprisingly, haematoxylin and eosin (H&E) staining revealed 8–12 sheets of fibroblasts in the outer dura (and extending to the outermost layer of the head), depositing extracellular matrix (ECM) that becomes compact in the adult dura (Fig. 4k,l).

### The inner dura expresses many tight-junction genes necessary to form a barrier

**Fig. 5:**
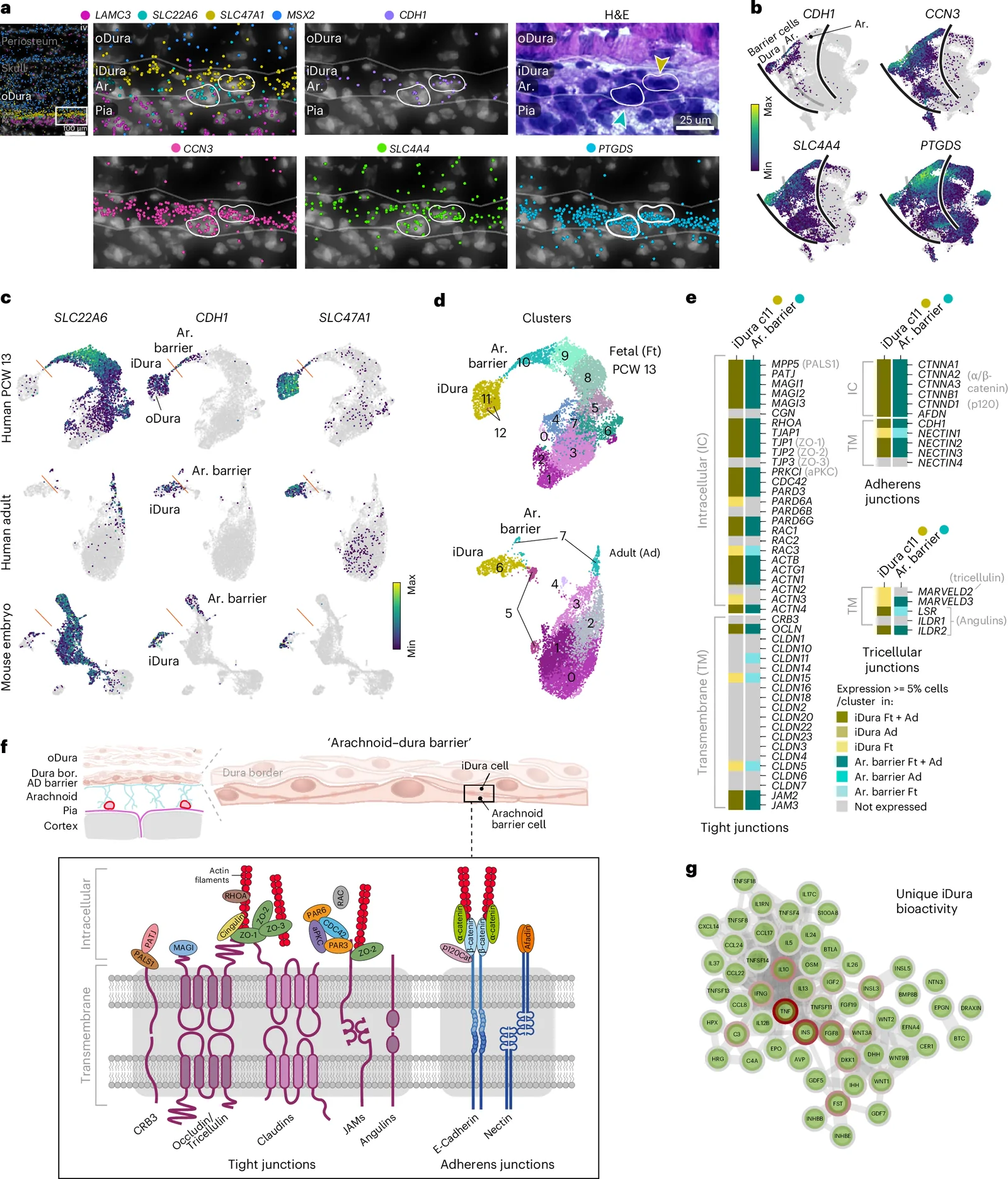
Analysis of the arachnoid barrier and inner dura. **a**, Spatial transcriptomics of *CDH1*, *CCN3* (also known as *NOV*), *SLC4A4* and *PTGDS*, and H&E staining at PCW 9.5 (*n* = 1). Ar., arachnoid; iDura, inner dura; oDura, outer dura. **b**, The same genes as **a** but coloured on scRNA-seq UMAPs of fibroblasts, on a grey background of all cells (*n* = 13). Black lines separate putative pia, arachnoid and dura lineages. **c**, UMAPs with *SLC22A6* (arachnoid), *CDH1* (barrier cells) and *SLC47A1* (inner dura) expression in human fetal PCW 13 (this study, *n* = 1), human adult^12^ (*n* = 3) and mouse embryonic^43^ (*n* = 42) fibroblasts. Orange lines indicate the separation between arachnoid and dura, based on *SLC22A6* expression. **d**, Clusters in fetal PCW 13 fibroblasts (this study) and adult fibroblasts^12^. Cluster colours match the expression of layer-specific genes in **a**. **e**, Stylized heatmaps showing the expression of genes encoding core components of tight-, adherens- and tricellular junction complexes, in clusters of fetal and adult inner dura and arachnoid barrier cells. Grey text indicates protein names where the gene names are dissimilar. **f**, Schematic illustration of our hypothesis that the arachnoid barrier is created between a layer of arachnoid barrier cells and the inner dura. Tight- and adherens junction proteins produced by genes in **e**, are shown, where the proteins left to right are in the same order as in **e** top to bottom, for intracellular and transmembrane domains. AD barrier, arachnoid–dura barrier. **g**, STRING interaction network of a uniquely bioactive gene signature in inner dura (*n* = 13 samples). Gene centrality outlined by red. Source data

**Extended Data Fig. 5:**
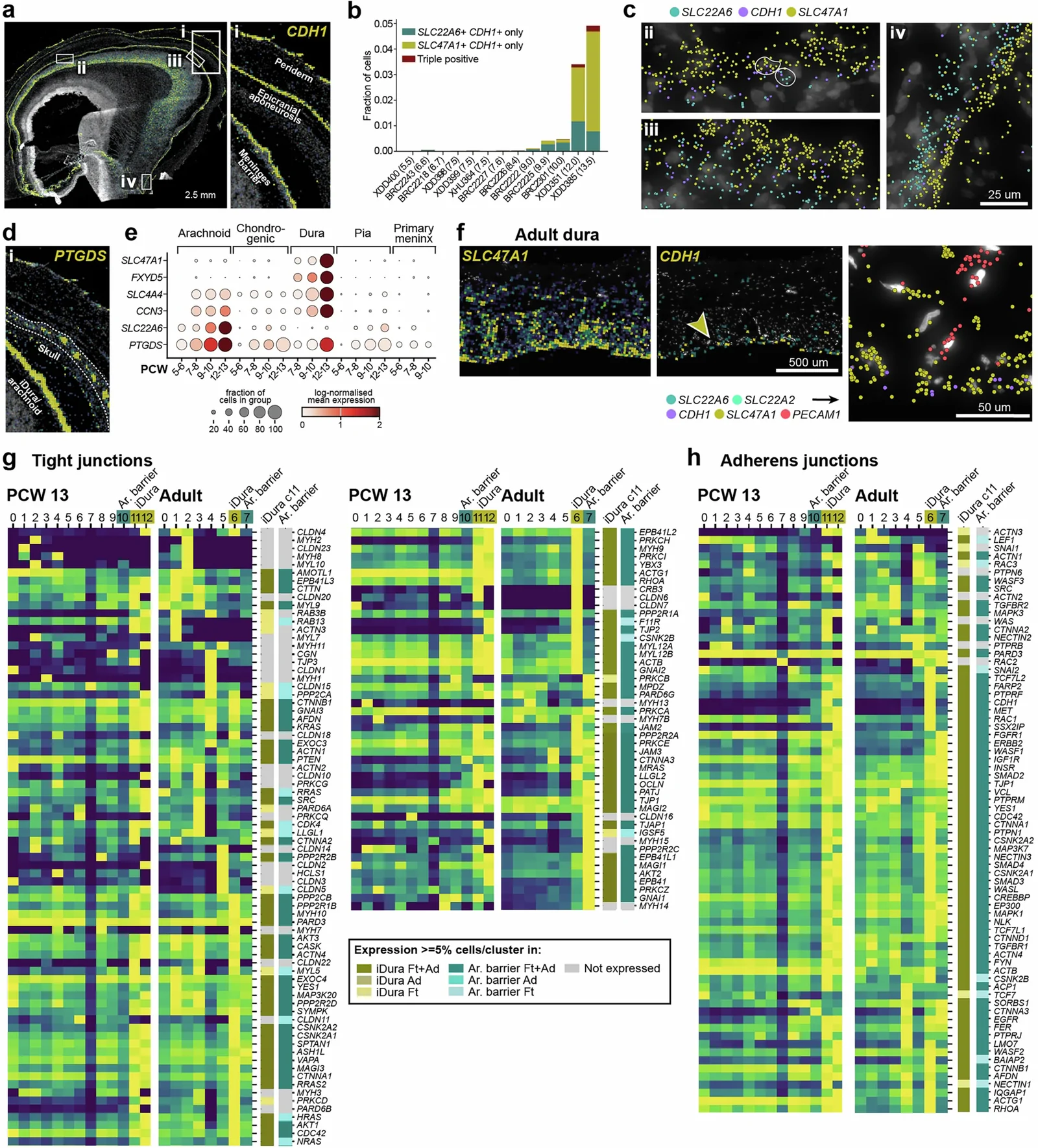
Shared and unique gene expression in inner dura and arachnoid barrier cells, related to Fig. 5. **a**, Spatial transcriptomics showing *CDH1* expression in PCW 9.5 head fibroblasts (n = 1). **b**, Stacked bar chart showing the fraction of *SLC22A6* + *CDH1*+ and *SLC47A1* + *CDH1*+ double positive cells, and *SLC22A6* + *SLC47A1* + *CDH1*+ triple positive cells across all donors. **c**, Spatial transcriptomics of *SLC22A6*, *SLC47A1*, and *CDH1* in three different telencephalic regions at PCW 9.5 (n = 1). Insets as in (A). White lines are drawn around two example cells. **d**, Same as **a** but *PTGDS* expression. **e**, Dotplot showing the expression of arachnoid and dura-specific genes over time, in arachnoid, chondrogenic, dura, pia, and primary meninx (n = 13 samples). **f**, Spatial transcriptomics showing *SLC47A1*, *CDH1*, *SLC22A2*, *SLC22A6* and *PECAM1* expression in human adult dura (representative image for n = 4 adult dura). **g**, Heatmap showing the expression of genes from the full KEGG list of tight junction genes (#M11355), in scRNA-seq clusters of PCW 13 (n = 1) and adult (n = 3) meningeal fibroblast. Right: Stylised heatmaps with a threshold criteria of genes being expressed in at least 5% of cells/cluster. **h**, Same as **g** but the full KEGG list of adherens junction genes (#M638). Source numerical data is in Source_Data_Fig5_EDFig5.xlsx, and code to reproduce the figure has been deposited at Zenodo under 10.5281/zenodo.21356807. Source data

The inner layer of the dura comprised a sheet of single cells, apposed onto the arachnoid. In the fetal head, the epithelial marker *CDH1* (E-cadherin) was expressed in three thin fibroblast layers: the meningeal barrier, the outermost periosteal layer (epicranial aponeurosis) and the periderm (Extended Data Fig. 5a). Focusing on the meningeal barrier, in mice, *Cdh1* is reportedly expressed only by arachnoid barrier cells^40^. In our data, *CDH1* was expressed by both the *SLC47A1*^+^ inner dura and *SLC22A6*^+^ arachnoid barrier precursors, forming two very closely aligned layers of cells from PCW 9 onwards (Fig. 5a,b and Extended Data Fig. 5b,c). The inner dura and arachnoid barrier precursors also shared expression of *CCN3* (also known as *NOV*), *SLC4A4* and *PTGDS* (Fig. 5a,b) among others. *PTGDS* has been reported to target the arachnoid^41^, but *Ptgds-Cre* lineage tracing in mice has shown that it is expressed in the whole meninges^42^. In our data, *PTGDS* expression progressively increased with age in all three layers, including the pia (Extended Data Fig. 5d,e).

These observations suggest that the inner dura layer may form a functionally distinct layer attached directly on top of the arachnoid, rather than being a simple continuation of the dura proper. Supporting this idea, the inner dura histologically resembled arachnoid barrier precursors rather than the outer dura (Fig. 5a, H&E). We noted further that arachnoid and dura cells in both human adult^12^ and mouse fetal meninges^43^ also expressed *CDH1* (Fig. 5c), demonstrating that this inner dura is an evolutionarily conserved feature that persists into the adult (Fig. 5c). Spatial transcriptomics of four human adult dura demonstrated that there existed *CDH1*^+^*SLC47A1*^+^ cells, and that were negative for *SLC22A6* and *SLC22A2* (Extended Data Fig. 5f).

To form a seal separating the dural and subarachnoid spaces, arachnoid barrier cells express tight- and adherens junctions. Interestingly, we found that genes encoding most of the components of a functional tight-junction complex (Fig. 5d–f) were also expressed in both the human fetal and adult inner dura (Fig. 5e, olive colour). Key genes included the transmembrane components *OCLN* (Occludin), a few *CLDN*s (Claudins) and *JAM*s, and the intracellular domains *MAGI*s, *TJP*s (Zona Occludens proteins), *PARD3*/*PARD6A-B* (PAR3/PAR6) and *CDC42*. Likewise, adherens junction genes were expressed, like *CDH1* (E-Cadherin), *NECTIN*s, *CTNNA*/*Bs* (α/β-catenin) and *AFDN* (Afadin) (Fig. 5e–f and Extended Data Fig. 5g,h). Finally, genes encoding four components of tricellular junctions were also expressed in the inner dura, *MARVELD2* (Tricellulin), *MARVELD3*, *LSR* (Angulin-1) and *ILDR2* (Angulin-3), which together are sufficient to form functional tricellular junction units (Fig. 5e).

Based on these observations, we propose the possibility that the complete meningeal barrier is formed by two *CDH1*^+^ layers of cells: the inner dura and the arachnoid barrier (‘Discussion’).

**Extended Data Fig. 6:**
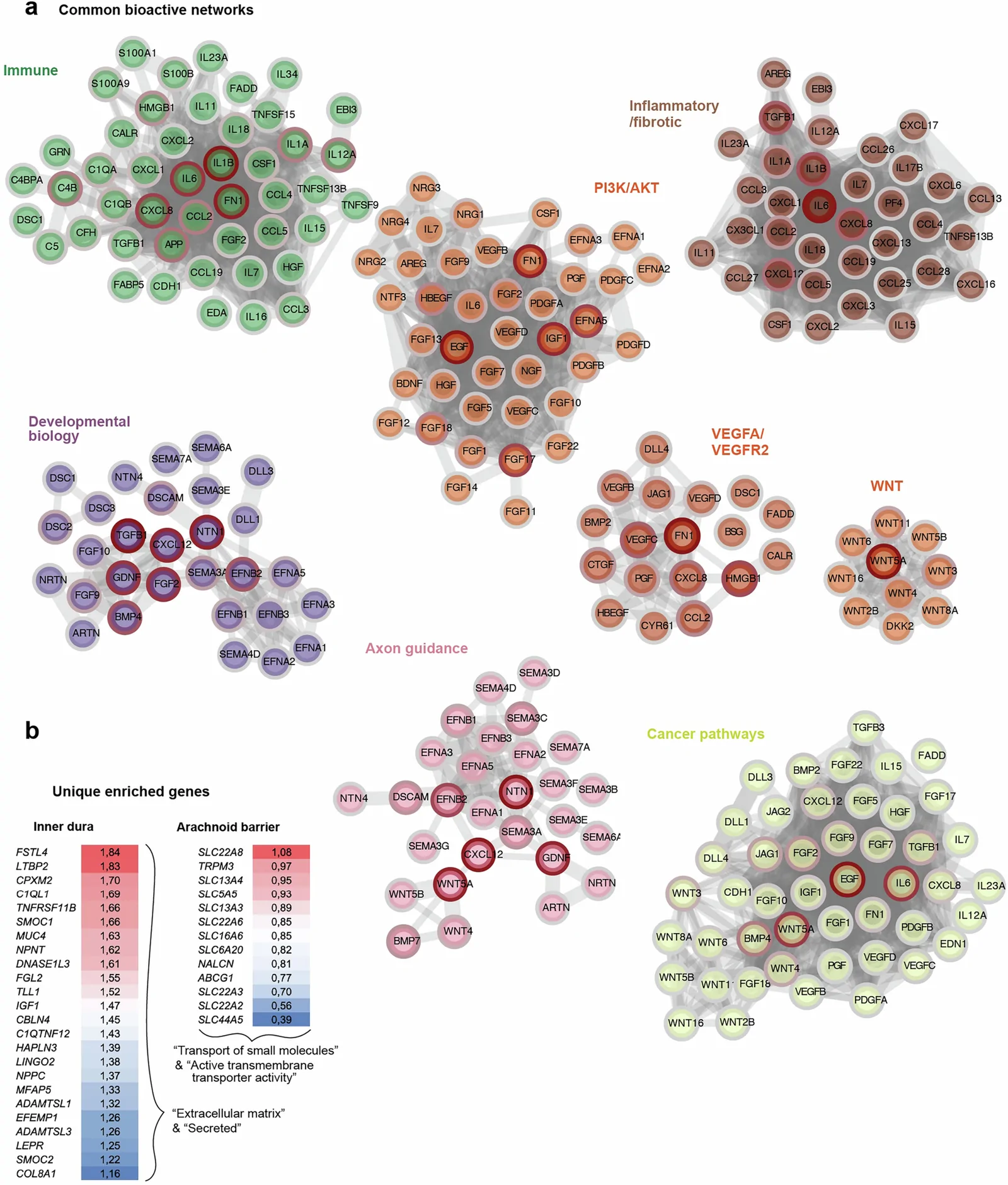
STRING interaction network analysis, related to Fig. 5. **a**, STRING interaction networks of shared bioactive genes between inner dura and arachnoid barrier clusters, split into subsets of significantly enriched functional pathways. Gene centrality (connectedness) outlined by red (n = 13 samples). **b**, Subset of enriched genes in inner dura and arachnoid barrier clusters, and relevant ontologies. Source numerical data and code to reproduce the figure has been deposited at Zenodo under 10.5281/zenodo.21356807.

We also found high bioactivity in the developing meningeal barrier (Supplementary Table 6). STRING network and pathway analysis showed that the inner dura and arachnoid barrier shared expression of genes involved in IL-6-centred inflammation and fibrosis; PI3K/AKT, WNT and VEGFA/VEGFR2 signalling; axon guidance; EGF–IL-6–WNT5A-centred cancer pathways; and an IL-1β–FN1-centred immune network (Extended Data Fig. 6a). However, inner dura cells uniquely had a strong TNF and INS-central cytokine and growth factor signature (Fig. 5g).

Ontologies of shared inner dura and arachnoid barrier enriched genes included ‘Cell junction’ and ‘Anchoring junction’ (Supplementary Table 6). However, uniquely enriched genes in the inner dura represented ‘Extracellular matrix’ and ‘Secreted’, while the arachnoid barrier was enriched by ‘Transport of small molecules’ and ‘Active transmembrane transporter activity’ via solute carriers (Extended Data Fig. 6b). Therefore, the function of the meningeal barrier may also include those of inner dura cells.

### Meningioma tumours are dura-like

Meningioma tumours are thought to arise from the meningothelial cells of the arachnoid (arachnoid cap and barrier cells) based on their histological similarities^6^. However, molecular evidence supporting the similarity of meningioma tumour cells to normal meningeal cell types is still lacking. To establish this, we re-analysed previously published single-cell and bulk RNA-seq data^19^, and generated new spatial transcriptomic data from seven meningiomas that we compared with our fetal datasets.

**Fig. 6:**
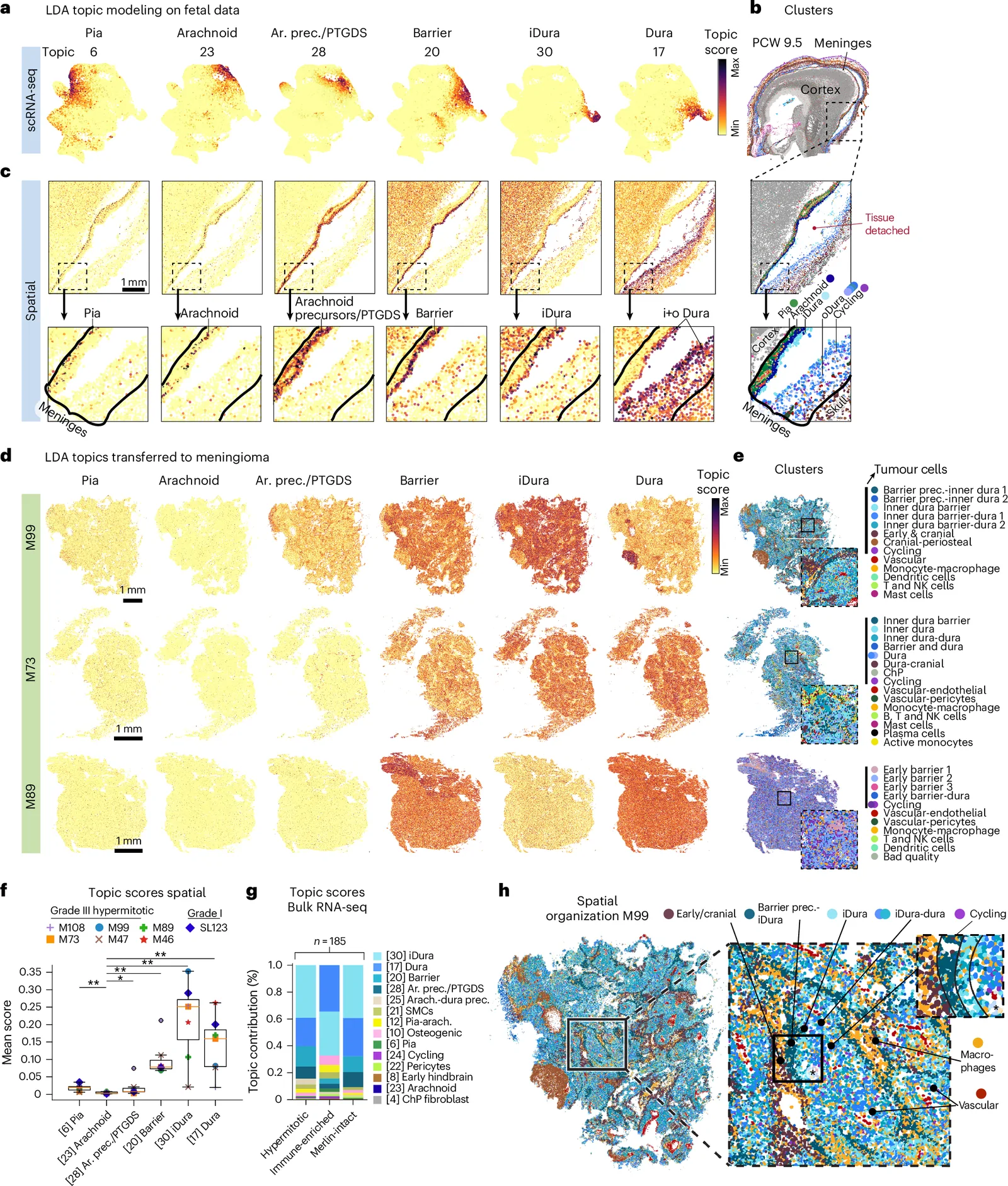
LDA topic modelling in fetal meninges and meningioma tumours. **a**, UMAP of fetal fibroblasts and perivascular cells (*n* = 13 samples), coloured by their LDA topic score, with topics annotated. Ar. prec., arachnoid precursor; iDura, inner dura; oDura, outer dura. **b**, PCW 9.5 spatial clusters (*n* = 1). Insets highlight clusters of meningeal layers. **c**, LDA topics transferred to fetal spatial data. Insets are the same as in **b**. **d**, LDA topics transferred to spatial data of grade 3 hypermitotic meningiomas M99, M73 and M89. **e**, Spatial clusters of tumours. Clusters were coloured by their most similar meningeal layer (for example inner dura-like cells were coloured cyan, as in **b**. **f**, Boxplots showing scores from LDA topic transfer to spatial data, from *n* = 7 meningiomas. Mann–Whitney *U* test with false discovery rate (FDR) multiple correction. *FDR <0.05, **FDR <0.01. FDR values for topics compared with topic 23: [topic 6] FDR = 0.0091, [topic 28] FDR = 0.0298, [topic 20] FDR = 0.0036, [topic 30] FDR = 0036, [topic 17] FDR = 0036. **g**, Stacked bar chart showing LDA topic scores transferred to bulk RNA-seq data from *n* = 185 meningiomas^19^, separated into three methylation profiles (grades I–III). **h**, Insets of M99 spatial clusters. Source data

**Extended Data Fig. 7:**
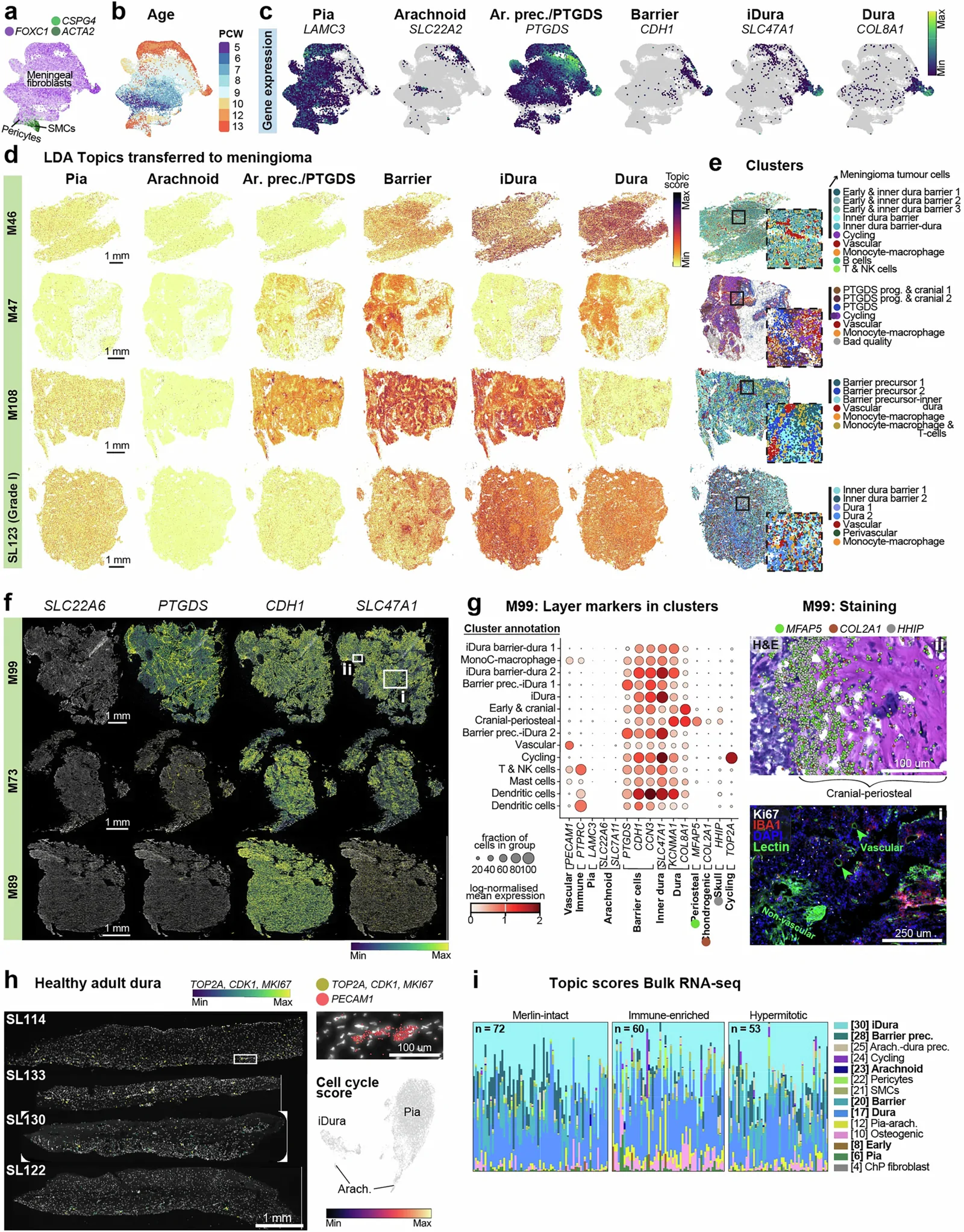
Further analysis demonstrating that meningioma tumours are dura-like, related to Fig. 6. **a**, Cells coloured by their expression of the gene they express most highly among *FOXC1* (meningeal fibroblasts & pericytes), *CSPG4* (pericytes), *ACTA2* (SMCs). n = 13 samples for **a-c**. **b**, UMAP of fetal fibroblasts and perivascular cells coloured by sample ages. **c**, Genes ranked highly by our LDA model for meningeal layer topics. Gene expression shown on UMAP of fetal fibroblasts and perivascular cells, on grey background of all cells. Ar. prec., Arachnoid precursors; iDura, inner dura. **d**, LDA topics transferred to spatial data from three high grade hypermitotic meningioma tumours; M46, M47 & M108, and one grade I tumour; SL123. **e**, Spatial clusters of tumours shown in **d**, coloured by their most similar meningeal layer. **f**, Spatial transcriptomic data showing *SLC22A6*, *PTGDS*, *CDH1*, and *SLC47A1* expression in M99, M73, and M89 tumours. Inset references i and ii are for **h**. **g**, Left: Dot plot showing gene expression of meningeal layer markers in M99 clusters. MonoC, Monocytes. Top right: Spatial transcriptomic data of *MFAP5*, *COL2A1* and *HHIP*, on H&E staining. Bottom right: Immunohistochemistry of Ki67, and IBA1, and DAPI and Lectin dyes (n = 1). Inset references i and ii can be seen in **f**. **h**, Spatial transcriptomic data of four human adult dura samples, showing RNA molecules of cycling cells (*TOP1A, CDK1, MKI67*) and endothelial cells (*PECAM1*). Cell cycle score on scRNA-seq UMAP of adult fibroblasts. **i**, Stacked bar chart showing scores of LDA topics transferred to bulk RNA-sequencing data from n = 185 meningioma tumours^21^. Source numerical data is in Source_Data_EDFig7.xlsx, and code to reproduce the figure has been deposited at Zenodo under 10.5281/zenodo.21356807. Source data

We processed ten sections from seven meningiomas (Supplementary Table 1) with spatial transcriptomics using the same gene panel as the fetal samples (Supplementary Table 3). To allow comparison with fetal meninges, we applied latent Dirichlet allocation (LDA) topic modelling^44^ to our scRNA-seq data from PCW 5–13 fibroblasts and perivascular cells (Fig. 6a and Extended Data Fig. 7a,b). Like clustering, LDA identifies topics—gene modules—that correspond to cell identities. In addition, it can reveal modules that are shared across cell types, such as cell cycle genes. We generated 35 topics (see details in the Methods) and identified those corresponding to the pia, arachnoid, *PTGDS*^+^ precursors, inner dura and dura (inner and outer) (Fig. 6a). We also identified one topic shared between arachnoid and inner dura cells; topic #20, ‘barrier’, with the junctional gene *CDH1* appearing as one of the topic-defining genes (Extended Data Fig. 7c and Supplementary Table 7). To validate these inferred topic identities, we transferred the topics to fetal spatial transcriptomic data, validating topic activity to the corresponding anatomical layers of the meninges. As expected, the barrier topic #20 was active in both arachnoid and inner dura layers (Fig. 6b,c).

Having thus confirmed LDA topic specificity to meningeal layers, we next transferred the topics to spatial data from one grade I and six grade III tumours (Fig. 6d–f and Extended Data Fig. 7d). Although tumour cells are highly abnormal, we reasoned that the expression of normal gene programs might still reveal their underlying cell identity. As expected, the pia topic was nearly absent. More surprisingly, the arachnoid topic was also absent, and only M108 expressed an arachnoid precursor topic. Instead, the most highly activated topics were the inner dura, dura and barrier. Notably, in normal meninges, the barrier topic was shared between arachnoid and inner dura cells, but the absence of arachnoid topic activity in meningiomas argues strongly that tumour cells were not arachnoid, but dural. The presence of meningeal gene programs also informed our annotation of meningioma clusters (Fig. 6e and Extended Data Fig. 7e). Furthermore, we did not notice any *SLC22A6* expression (arachnoid), while *CDH1* (barrier) and *SLC47A1* (inner dura) markers were clearly expressed. Consistently, where *PTGDS* was expressed, it was in non-arachnoid cells (Extended Data Fig. 7f,g). Notably, some tumour cells expressed periosteal, chondrogenic and cranial markers (*MFAP5*, *COL2A1* and *HHIP*) (Extended Data Fig. 7g, dot plot, and Supplementary Table 4) and had a bone-like appearance with H&E staining (Extended Data Fig. 7g). Immunohistochemistry of tumours also confirmed cycling cells, vasculature, macrophages and lectin-positive non-vascular regions (Extended Data Fig. 7g). While cycling tumour cells expressed *CDH1*, *CCN3*, and *SLC47A1*, and were therefore inner dura-like, the only cycling cells in normal adult dura were vascular, and not dural fibroblasts (Extended Data Fig. 7h).

We also extended the LDA analysis to a previously published bulk RNA-seq dataset of 185 grade I–III meningiomas^19^ (Fig. 6g and Extended Data Fig. 7i). In agreement with our spatial data, these bulk datasets showed very low activity of pia and arachnoid topics. Instead, inner dura and dura topics dominated, followed by the barrier topic in hypermitotic and merlin-intact meningioma subtypes, and an osteogenic topic in immune-enriched meningiomas.

**Extended Data Fig. 8:**
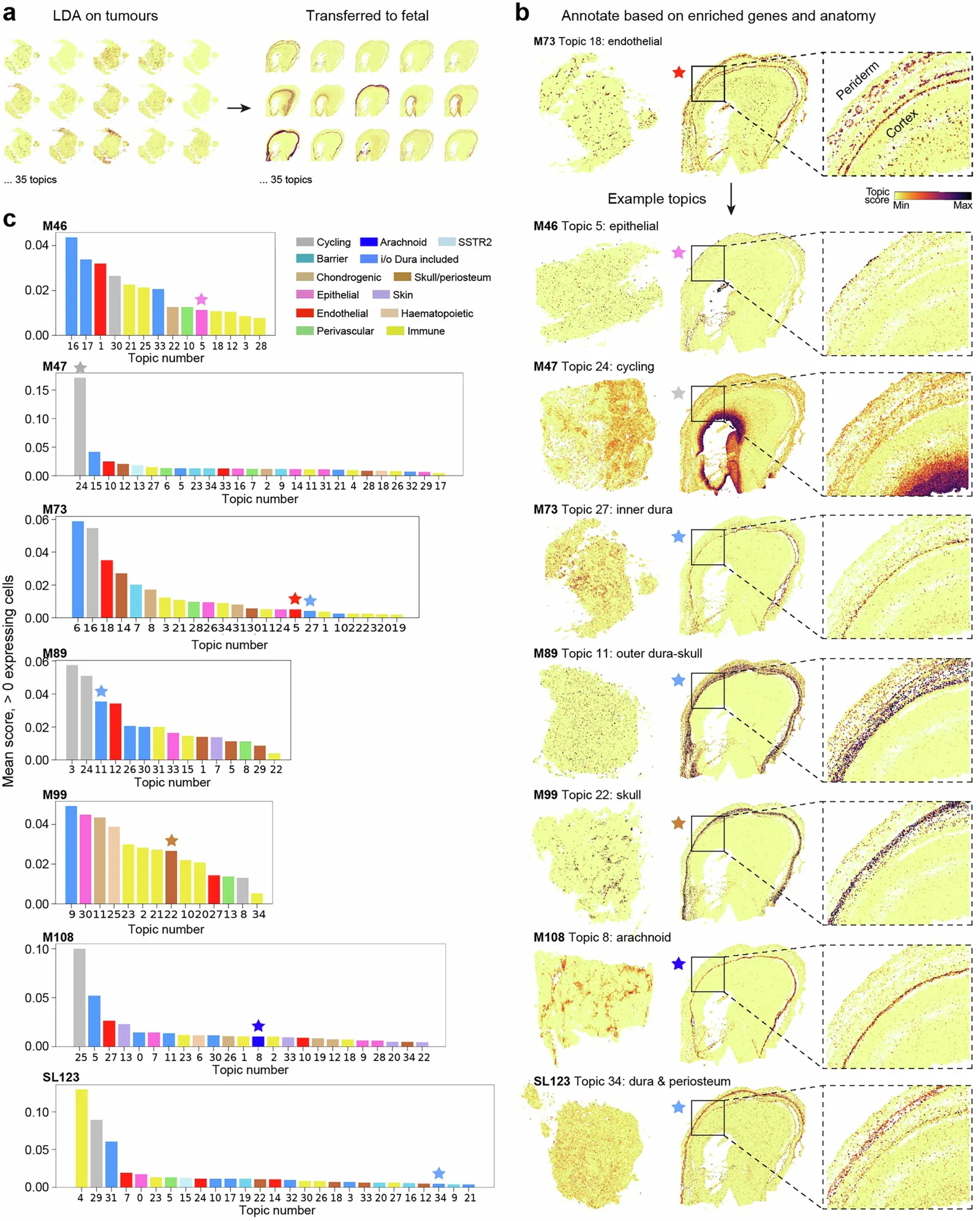
LDA topic analysis of tumour spatial data, related to Fig. 6. **a**, Strategy to generate LDA topics on tumour spatial data, and transfer them to PCW 9.5 fetal spatial data to map gene programs by anatomy. **b**, Scatterplots of spatial data, coloured by their LDA topic score (n = 7). Examples of annotated topics for each tumour, and their transferred scores in fetal data. Stars indicate the plotted topic in **c**. **c**, Bar chart showing quantification of topic scores in >0 expressing cells for each tumour. The bars are coloured by their annotation, also matched in Supplementary Table 8. Stars indicate the plotted topic in **b**. Source numerical data is in Source_Data_EDFig8.xlsx, and code to reproduce the figure has been deposited at Zenodo under 10.5281/zenodo.21356807. Source data

As a control, we generated LDA topics directly from the tumour spatial data, too, and transferred the tumour topics to fetal spatial data to validate layer resemblance (Extended Data Fig. 8a). Consistent with the above, all tumours contained a combination of topics resembling either barrier precursors, the inner dura, dura and/or chondrogenic and osteogenic populations. Only tumour M108 contained, in addition to dural topics, an arachnoid topic (Extended Data Fig. 8b,c and Supplementary Table 8), consistent with our results in Fig. 6f. Overall, these observations strongly suggest that meningiomas predominantly contain dura-like cells. It also raises the intriguing hypotheses that meningiomas (1) originating from arachnoid barrier cells later develop into dura-like cells and/or (2) could originate from dural cells, instead of, or in addition to, the arachnoid.

**Extended Data Fig. 9:**
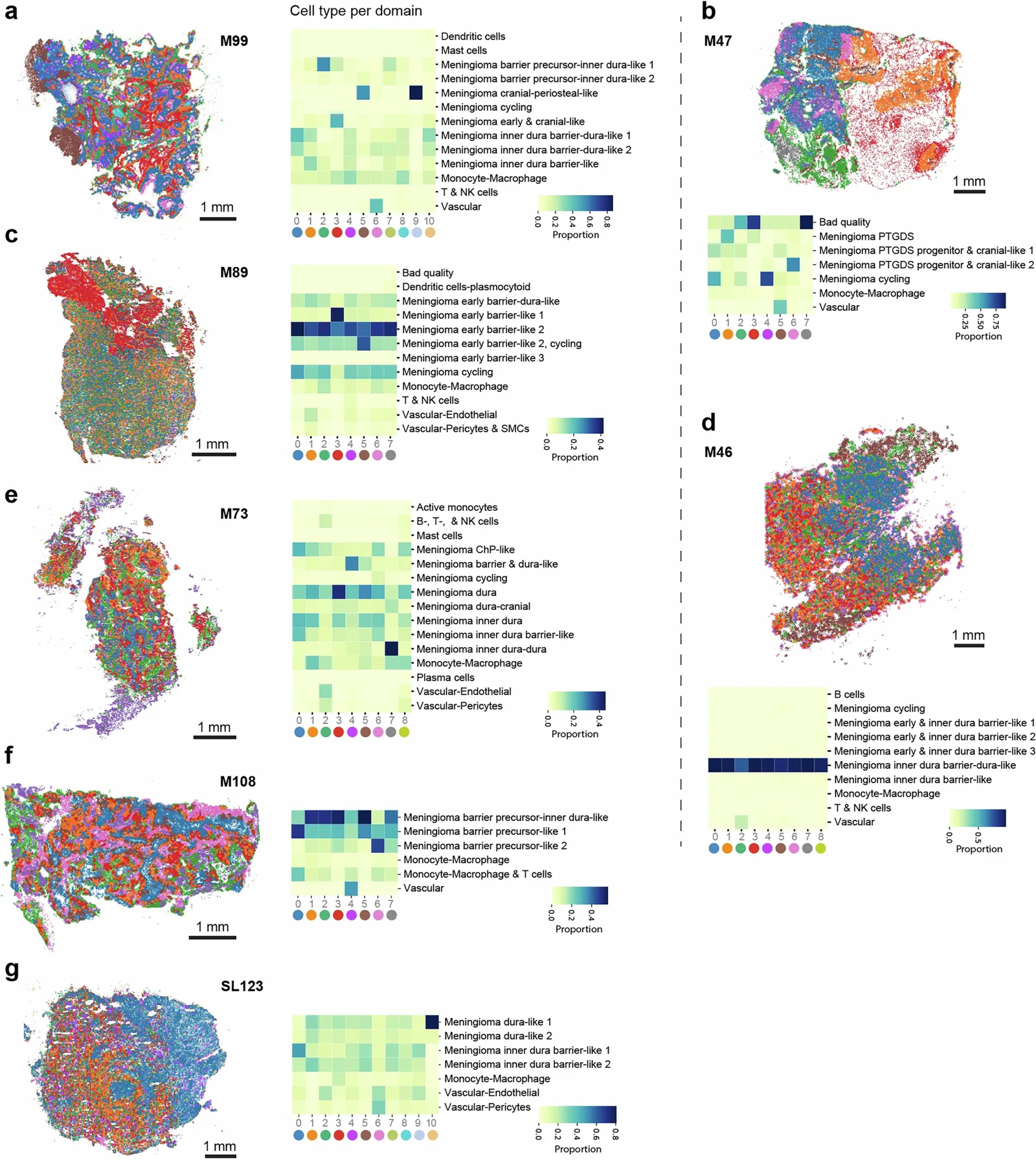
Banksy domain analysis of tumour spatial data, related to Fig. 6. **a**, Spatial plot coloured by domains, and heatmap of proportion of cell types per domain, in tumour M99. See also Supplementary Table 9. **b**, Same as **a** for tumour M47. **c**, Same as **a** for tumour M89. **d**, Same as **a** for tumour M46. **e**, Same as **a** for tumour M73. **f**, Same as **a** for tumour M108. **g**, Same as **a** for tumour SL123. Source numerical data and code to reproduce the figure has been deposited at Zenodo under 10.5281/zenodo.21356807.

Furthermore, spatial visualization of cell type clusters, and a Banksy spatial domain analysis (Supplementary Table 9) showed that some tumours were highly spatially structured in a manner that resembled a jumbled version of dural and early cranial development, with vascular and immune cells interspersed (Fig. 6h and Extended Data Fig. 9). In other tumours, however, cells appeared mixed more uniformly (Fig. 6e and Extended Data Figs. 7e and 9).

### The dura lineage expresses genes frequently mutated in meningioma

**Fig. 7:**
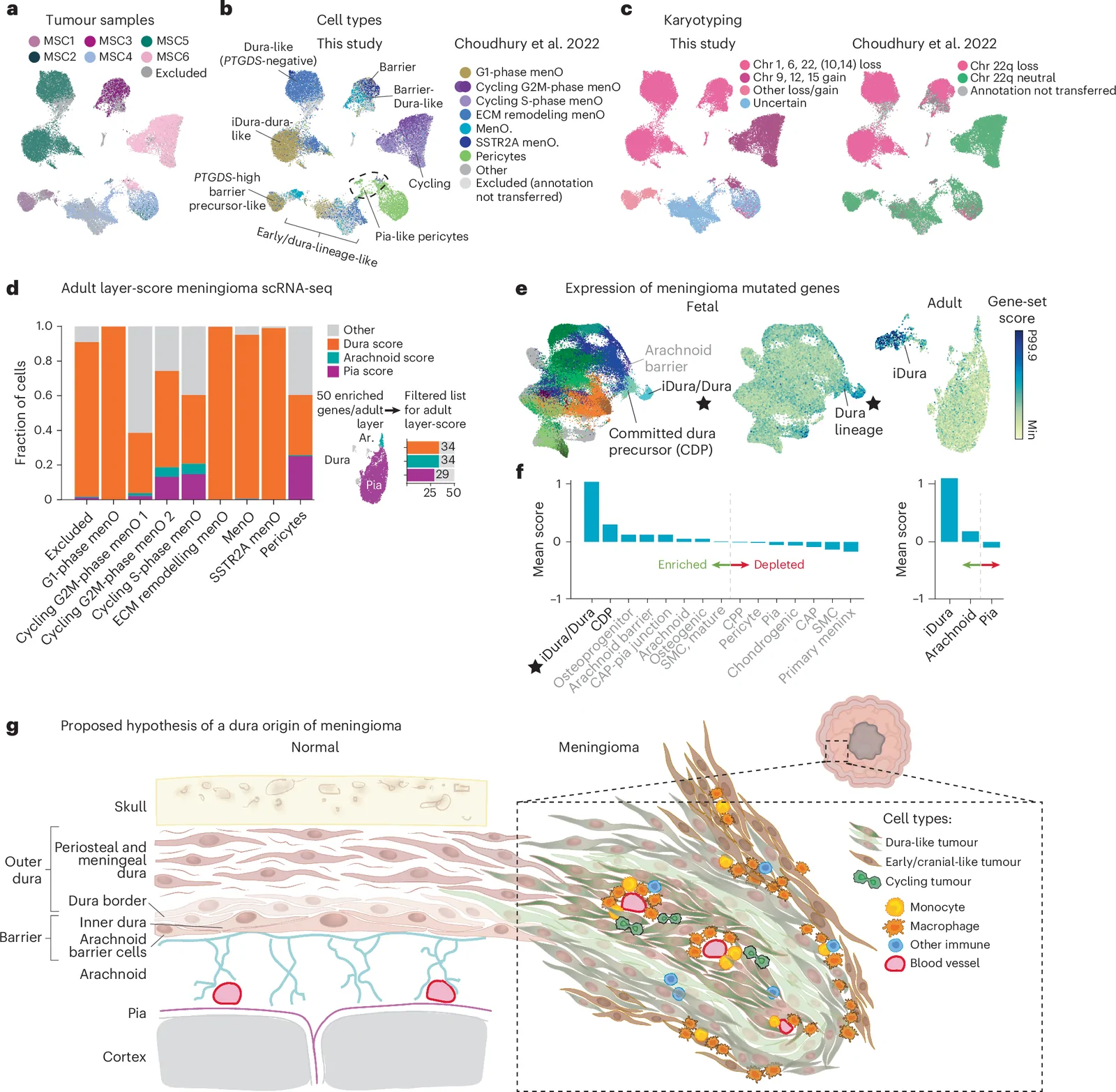
Genetic analysis supporting a dura origin of meningioma tumours. **a**, UMAP of pooled but not harmonized tumour cells and pericytes, coloured by samples (*n* = 6). Data from Choudhury et al.^19^. **b**, UMAP coloured by transferred annotations^19^, with additional annotations added in this study. Arach., arachnoid; iDura, inner dura; menO, meningioma. **c**, UMAP coloured by karyotype. **d**, Stacked bar chart showing the fraction of tumour cell types expressing enriched genes from adult meningeal layers (as in Fig. 4). Lane for immune cells not shown because we focused on tumour cells. **e**, Left: UMAP of fetal fibroblasts and perivascular cells, coloured by subclass. Right: UMAPs of fetal fibroblasts and perivascular cells and adult fibroblasts^12^, coloured by a gene-set score of the top 20 mutated genes in meningioma, minus *TERT* (https://cancer.sanger.ac.uk/cosmic/browse/tissue). **f**, Mutated gene-set score as in **e** quantified per fetal subclass. Stars indicate the same cells across **e** and **f**. CDP, committed dura precursor; CAP, committed arachnoid precursor; CPP, committed pia precursor. **g**, Schematic illustration of healthy meninges, and the proposed formation of meningioma tumours from dura cells. Source data

**Extended Data Fig. 10:**
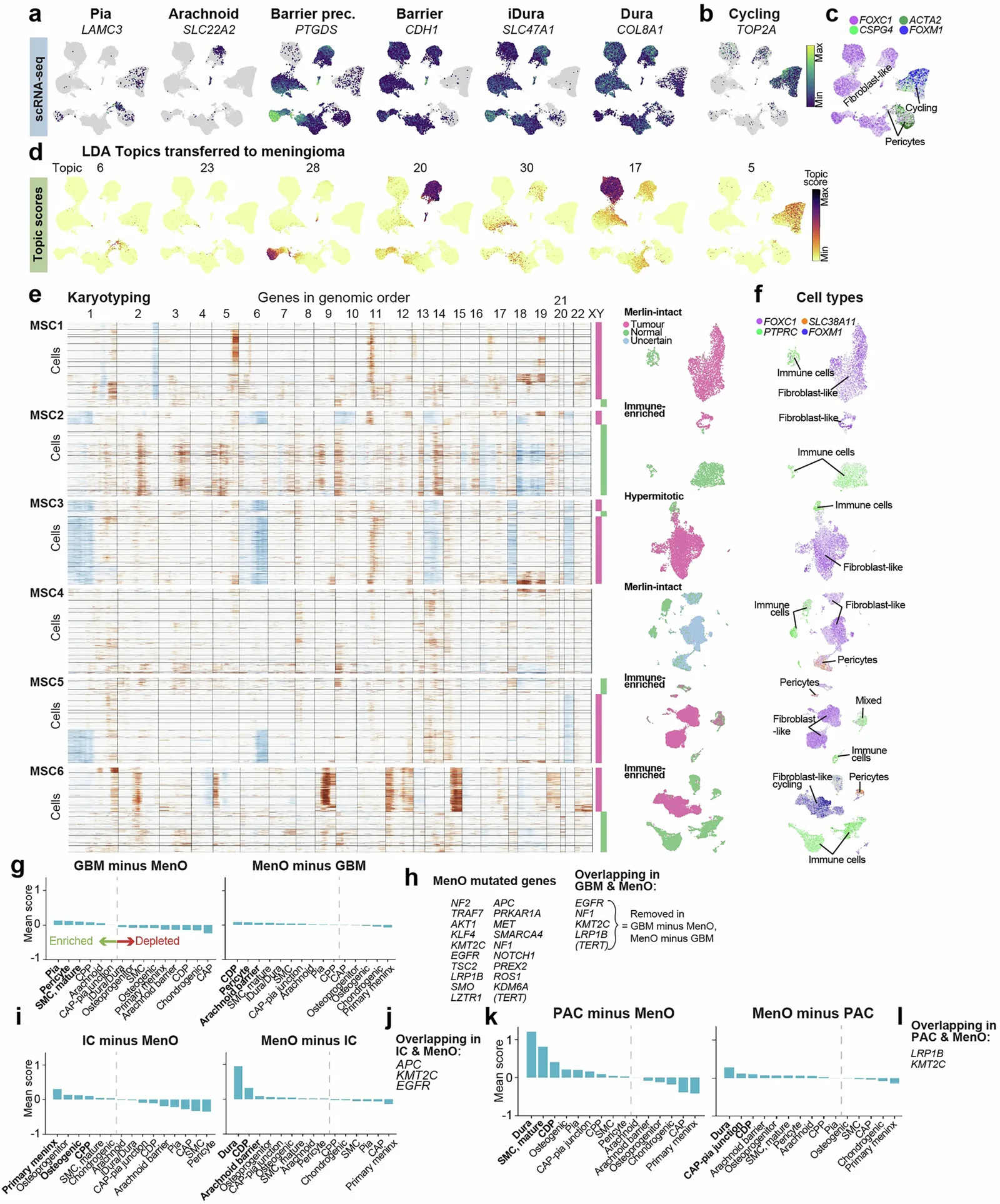
Analysis of meningioma scRNA-seq, and mutated genes from various cancers in fetal meningeal fibroblasts, related to Fig. 7. **a**, UMAP of tumour cells, coloured by the expression of genes ranked highly by our LDA model for meningeal layer topics, on a grey background of all cells (n = 6 for whole figure). **b**, UMAP of tumour cells coloured by *TOP2A* expression. **c**, Cells coloured by their expression of the gene they express most highly among *FOXC1* (meningeal fibroblasts & pericytes), *CSPG4* (pericytes), *ACTA2* (SMCs), and *FOXM1* (cycling tumour). **d**, LDA topics for meningeal layers and cycling cells (Topic 5) transferred to meningioma scRNA-seq data. **e**, Karyotyping of individual tumours. Chromosomal deletions are blue, and gains are brown. Bars to the right and UMAPs of individual tumours indicate cells that are tumour (pink), normal (green) and of uncertain karyotype (light blue). **f**, Annotation of individual samples, and expression of *FOXC1* (meningeal), *SLC38A11* (pericytes), *ACTA2* (SMCs), *PTPRC* (immune). **g**, Quantification of gene-set score of top 20 mutated genes in glioblastoma (GBM) tumours minus genes overlapping with top 20 mutated genes in meningioma (MenO), and MenO minus GBM overlapping genes, in fetal meningeal fibroblast and perivascular cell Subclass (as in Fig. 7f. Analysis shows average of n = 10 runs). **h**, Top 20 mutated genes in meningioma, and genes overlapping between the top 20 mutated genes in GBM and MenO. **i**, Same as **g** but for intestinal carcinomas (IC). **j**, List of overlapping genes between the top 20 mutated genes in IC and MenO. **k**, Same as **g** but for prostate adenocarcinomas (PAC). **l**, List of overlapping genes between the top 20 mutated genes in PAC and MenO. Source numerical data is in Source_Data_EDFig10.xlsx, and code to reproduce the figure has been deposited at Zenodo under 10.5281/zenodo.21356807. Source data

Further extending our analysis to a previously analysed scRNA-seq dataset of six meningiomas (grades I–III)^19^ (Fig. 7a), we found again that barrier, inner dura and dura topics dominated in separate clusters (Extended Data Fig. 10a–d). Notably, our ‘Dura-like meningioma’ overlapped with the previously annotated ‘ECM remodelling meningioma’, and a subset of pericytes were ‘pia-like’ (Fig. 7b and Extended Data Fig. 10a–d). By applying an improved karyotyper algorithm (Methods) to this scRNA-seq dataset, we found that most cycling cells were derived from tumour MSC6, which carried a distinct karyotype (chromosome 9, 12 and 15 gain) and lacked the typical chromosome 22 loss that was used to define tumour cells in the original publication (Fig. 7c and Extended Data Fig. 10e).

The LDA topics above were defined on developing meninges, but tumours are found in postnatal brains. As an alternative indicator of cell identity, we used adult layer-specific genes (as in Fig. 4) and examined their expression in tumour cells. We found that, in each subtype of tumour cells, the dura score dominated. Arachnoid scores were very low, and pia scores were present in some cycling populations and pericytes (Fig. 7d).

As an independent line of evidence, we reasoned that both tumour suppressors and driver genes must be normally expressed in the cell of origin, before oncogenic transformation. We collected the 20 most frequently mutated genes in meningiomas, (omitting *TERT*; Methods; Extended Data Fig. 10h) and analysed their collective expression in normal fetal and adult meninges. Intriguingly, the cells with by far the highest expression of these putative meningioma cancer drivers were those of the dural lineage (Fig. 7e,f). To reduce the influence of common cancer driver genes shared with other cancer types, we repeated the analysis after removing genes commonly mutated also in glioblastoma, intestinal cancer or in pancreatic cancer (Supplementary Table 10). In each case, inner dura/dura and/or committed dura precursors remained the most enriched for expression of cancer drivers (Extended Data Fig. 10g–l). While future mechanistic data are necessary to establish a new model, these results lend further independent support to the possibility that meningioma tumours could arise from cells of the dural lineage (Fig. 7g).

## Discussion

Fibroblasts and mesenchymal cells are poorly described in general, partly due to their unconventional lineage plasticity^45,46^. This often leads to ambiguous descriptions of cellular subtypes and function, and poor subtype markers. Spatial transcriptomics greatly aided in generating a more detailed annotation of fetal craniofacial and meningeal fibroblasts. By using scRNA-seq and spatial transcriptomics we molecularly described the earliest progenitors of pia, arachnoid and dura mater. We showed that meningeal layer development is defined by gene expression gradients, and occurs concurrently. We also provided previously undescribed insights into human fetal immune cells, vasculature and ChP.

Here, we hypothesize that inner dura cells form a second barrier sheet on top of the arachnoid. We discovered a fibroblast type transcriptionally similar to dura cells (*FXYD5* and *SLC47A1*) but expressing *CDH1*, that is, ‘inner dura’ cells forming a single sheet directly on top of *CDH1*^+^ arachnoid barrier precursors. The current literature reports that only arachnoid barrier cells express *CDH1*^40^, and *CDH1*^+^*PTGDS*^+^ cells have been annotated as the arachnoid barrier^11^. The first scRNA-seq paper of mouse E14.5 *Col1a1*^+^ cells found two clusters expressing *Cdh1*, one arachnoid (cluster ‘M3’) and one dura (‘M4’). However, it was reported that only the ‘M3’ arachnoid cluster expressed tight-junction genes^8^. Interestingly, our inner dura cells expressed tight-junction genes and resembled arachnoid barrier cells histologically. This raises the intriguing possibility that the inner dura layer is epithelial (*CDH1*) and that the complete meningeal barrier is formed by two layers of cells: the inner dura and the arachnoid barrier. This model is further supported by electron microscopy^10^, which has shown the meningeal barrier to be formed by precisely two layers of cells. Our findings are not necessarily inconsistent with mouse models of arachnoid barrier layer development^47^, but show that the use of *CDH1* (and *PTGDS*) does not discriminate between the arachnoid barrier and inner dura, and therefore propose that the composition of the ‘arachnoid barrier’ may include the inner dura. To confirm the barrier function of the inner dura, the expression of tight-junction proteins needs to be validated, together with functional experimentation. If confirmed, we propose renaming the arachnoid barrier to the ‘arachnoid–dura barrier’, and the *CDH1*^+^ inner dura cells to ‘dura barrier cells’.

Recent efforts have refined meningioma classifications based on integrative multidimensional molecular profiling, paired with clinical parameters to more accurately reflect clinical outcomes^48–51^. Surprisingly, however, meningiomas have not been described on the basis of their molecular similarity to meningeal layers. Potentially, this has been due to the longstanding acceptance of the meningothelial arachnoid cell origin^52,53^, and the existence of very few meningeal molecular and cellular atlases^8–11^ to compare with. Here, we leveraged our comprehensive atlas of human meninges development to show that meningioma cells are predominantly dura lineage-like. Instead, arachnoid gene signatures were depleted. Our results prompt the hypothesis that inner dura cells could be a meningioma progenitor cell, because they (1) expressed *CDH1*, as did meningioma, (2) were located in the meningeal barrier, consistent with previous anatomical evidence, (3) histologically resembled arachnoid barrier cells, (4) of all the topics, inner dura cells (topic 30) were the most consistent in all meningiomas, and (5) expressed cancer driver genes. The fact that genetic driver genes of meningioma were enriched in inner dura cells suggests that these cells are particularly vulnerable to cancer transformation.

Previous studies generated meningiomas from *PTGDS*-expressing primordial meningeal cells in mouse-models^54,55^. However, we found that *PTGDS* does not distinguish the arachnoid barrier from the inner dura. While our results challenge the view of arachnoid meningothelial cells as the predominant meningioma cell of origin, our findings are not necessarily inconsistent with previous findings, just that they did not discriminate between arachnoid barrier and inner dura cells experimentally. Considering the clinical diversity of meningioma tumours it is likely that other progenitor cells drive or contribute to meningioma tumorigenesis as well, such as *NOTCH3*^+^ mural cells^42^. Future experiments will be necessary to test our hypothesis and establish whether cells of the dura lineage can generate meningiomas, and whether different tumour subtypes might arise from arachnoid barrier cells versus dural cells.

Our study has several limitations. Fetal tissue availability was a limiting factor; for example, we did not access PCW 11 meninges. Most dura cells in our scRNA-seq atlas expressed *CDH1*; therefore, we captured mostly inner dura. Likewise, we aspired to collect the arachnoid and whole dura from adult patients for spatial analysis; however, due to surgical limitations, we mostly obtained the dura border to some periosteal dura.

In this study, we mainly focused on hypermitotic grade 3 meningiomas for spatial transcriptomics. However, meningiomas have 15 histological subtypes^56^. While we performed LDA topic transfer to bulk RNA-seq of 185 meningiomas (grades 1–3), further investigation is necessary to conclude whether our observations apply to other subtypes or clinical parameters (age, sex, survival and so on).

## Methods

### Donors

Human prenatal samples were collected from elective medical abortions at the Department of Gynecology, Danderyd Hospital and Karolinska Huddinge Hospital, and Addenbrooke’s Hospital in Cambridge, following oral and written informed consent by the patient. In Sweden, the use of abortion material was approved by the Swedish Ethical Review Authority and the National Board of Health and Welfare. In the UK, approval from the National Research Ethics Service Committee East of England – Cambridge Central was obtained (Local Research Ethics Committee, 96/085). No compensation was provided to the donors.

### Patient samples

Human dura samples and one meningioma sample (‘SL123’) were collected from the Karolinska Hospital with informed consent from the patients and with ethical approval from the Swedish Ethical Review Authority (2020-03505). The use of samples was approved by the Swedish Ethical Review Authority (2020-02096).

Meningioma samples were collected from the University of California San Francisco (UCSF), complied with all relevant ethical regulations, and was approved by the UCSF Institutional Review Board (13–12587, 17–22324, 17–23196 and 18–24633). As part of routine clinical practice at UCSF, all patients who were included in this study signed a written waiver of informed consent to contribute deidentified data to research projects.

#### Population characteristics

##### Sex

Fetal samples were collected and processed as soon as possible; hence, the sex of the sample could not be determined ahead of time. Therefore, the sex was inferred afterwards based on expression of the gene *XIST*. For scRNA-seq, data from seven female and six males individuals was collected. The sex of two fetal samples used for Xenium and IHC is unknown. Sex was reported for adult patient samples (meningioma and dura), where data from five female and six male individuals was collected.

##### Demographics

Samples were collected with anonymity from Sweden, the UK and the USA, which might bias the biological variability. The donation procedures did not allow the collection of data regarding race, ethnicity or, for example, maternal health (for fetal samples).

Details about all samples can be found in Supplementary Table 1.

### Reagents and resources

A detailed list of all reagents and resources is available in Supplementary Table 11.

### Tissue sample collection

#### Human fetal meninges

Fourteen samples of human prenatal meninges were used in this study, at PCW 5–13. Two samples of fetal heads were used, at PCW 6 and 9.5. The postconception age of the embryos and fetuses was estimated by information from the clinical ultrasound, last menstrual period, true crown–rump length and age-dependent anatomical landmarks. However, the precision of determining the PCW should be considered as ±0.5 weeks.

For Karolinska Hospital samples, the tissue was immediately transported to the laboratory following abortion, and dissected in ice-cold 0.9% NaCl solution under sterile conditions within 1–2 h post abortion. For scRNA-seq, meninges were dissected and kept in ice-cold Hibernate-E medium until further processing. For spatial analysis, fetal heads were covered by Tissue-Tek Optimal Cutting Temperature compound (OCT) in cryomolds, snap-frozen in a slurry of 2-methylbutane (Sigma-Aldrich) and dry ice, and stored at −80 °C pending sectioning.

For Cambridge samples, tissues were dissected in a class II hood on the day of collection and stored in Hibernate-E medium at 4 °C. The tissue was shipped to Sweden at refrigerated temperature, and delivered 2 days after abortion. The procedure is covered under ethics REC: 96/085.

#### Human adult dura

Four dura samples were collected from patients undergoing surgery for low-grade gliomas and subependymoma (that is, non-invasive, and meninges unaffected). During surgery, dura was placed in Hibernate-E medium on ice and transported as such. Upon arrival, samples were washed in fresh Hibernate-E, then embedded in OCT, and snap-frozen in 2-methylbutane and dry ice slurry. Samples were stored at −80 °C until further use.

#### Human meningioma

Six meningioma tumour samples that were resected from 1991 to 2016 and snap-frozen were identified from an institutional biorepository and clinical database at the UCSF. World Health Organization (WHO) grading was performed using contemporary criteria outlined in the WHO 2016 and 2021 classification of tumours of the CNS. Samples were shipped to Sweden on dry ice, and stored at −80 °C. Upon use, samples were embedded in OCT for cryosectioning. ‘SL123’ meningioma was collected at the Karolinska Hospital as the dura samples above.

### Cell dissociation

Dissected meninges were processed around 6–48 h after tissue collection, depending on the source (Karolinska Hospital/Cambridge). Tissues were stored at 4 °C in Hibernate-E medium during transport from Cambridge and until processing. Ice-cold Earle’s Balanced Salt Solution (Worthington) was carbogenated (95% O_2_/5% CO_2_) and used throughout the whole procedure. Meninges were dissociated using the Worthington’s Papain Dissociation System (Worthington) (https://doi.org/10.17504/protocols.io.xmbfk2n). Tissues were enzymatically digested at 37 °C for 10–15 min, followed by trituration using fire-polished glass Pasteur pipettes. Cell suspensions were filtered through a 30-μm cell strainer (CellTrics, Sysmex) and centrifuged for 5 min at 200*g* to obtain cell pellets. Supernatants were carefully removed, and cells were resuspended in small volumes of Earle’s Balanced Salt Solution (depending on cell density). Cell concentrations were estimated using a counting haemocytometer (Bürker/Neubauer chamber) and diluted with Earle’s Balanced Salt Solution until the desired concentrations were reached. All suspensions were kept on ice until loading on the 10x Chromium chips.

### scRNA-seq

Droplet-based scRNA-seq was performed using the 10x Genomics Chromium Single Cell Kit v3. Single-cell suspensions, at a concentration of 800–1,200 cells ml^−1^, were mixed with master mix and nuclease-free water according to the Chromium manual, targeting 5,000–10,000 cells per reaction. Twelve PCR cycles were used for cDNA synthesis, and the rest of the library preparation was performed according to the manufacturer’s instructions (10x Genomics, Illumina). All libraries were sequenced on an Illumina NovaSeq 6000 using S4 to a target sequencing depth of 100,000 reads per cell. Sequencing saturation was examined for each sample using preseq (https://github.com/smithlabcode/preseq).

### Cryosectioning

Fresh-frozen tissue was embedded in OCT as above. The tissue was acclimatized in the cryostat chamber, and the specimen/chuck was set to −16 °C and the knife to −14 °C (for fetal heads, dura and meningiomas). The samples were sectioned at 10 μm and collected on Xenium slides following the manufacturer’s recommendations, or captured on Superfrost Plus Adhesion Microscope Slides for other stainings as below.

### Xenium in situ gene expression

Fresh-frozen 10-μm tissue sections were placed on Xenium slides. The next steps were performed at the In Situ Sequencing Facility at SciLifeLab. The tissue was fixed and permeabilized according to the Xenium Fixation and Permeabilization Protocol (Demonstrated Protocol CG000581). A predesigned Human Lung panel (289 genes) and a custom gene panel (100 genes; Supplementary Table 3) were applied to the tissue. Probes were hybridized to target RNA, followed by ligation and enzymatic amplification to generate multiple copies of each RNA target, as outlined in the Probe Hybridization, Ligation, and Amplification User Guide (User Guide CG000582). The prepared Xenium slides were subsequently loaded onto the Xenium Analyzer for imaging and analysis, following the Decoding and Imaging User Guide (User Guide CG000584). Instrument software version 1.4.3.1 and software analysis version 1.4.0.7 were used throughout the process.

### Post-Xenium H&E staining

To remove autofluorescence quencher from the post-Xenium slides, tissues were incubated in 1.74 mg ml^−1^ sodium hydrosulfite dissolved in Mili-Q water for 10 min. The slides were then rinsed three times in Mili-Q water for 1 min, followed by a 2-min wash with distilled water (dH_2_O). The slides were subsequently washed three times with 1× PBS and three times with dH_2_O for 5 min. Haematoxylin solution (H&E stain kit) was applied to each section for 1 min and rinsed in two changes of dH_2_O. Bluing agent (H&E stain kit) was applied to each section for 15 s, and the slide was rinsed twice with dH_2_O. The slide was dipped in ethanol, and excess >99.5% ethanol was blotted off. Eosin Y solution (H&E stain kit) was applied to each section for 30 seconds and rinsed in ethanol. The sections were subsequently dehydrated in three changes of >99.5% ethanol for 2 min each and rinsed in xylenes, before mounting with Entellan new. The slide was left to solidify for 24 h in a ventilated hood.

### Immunohistochemistry cryosections

The supplier information and dilution used for each antibody is provided in Supplementary Table 11. Fresh-frozen tissue samples were cryosectioned at 10 μm and captured on Superfrost Plus Adhesion Microscope Slides. A hydrophobic barrier was drawn around tissue sections using ImmEdge Hydrophobic Barrier PAP Pen. The sections were fixed with 4% formaldehyde in 1× PBS for 10 min. The slides were washed with 1× TBS for 5 min, then three changes of 0.025% Triton X-100 in 1× TBS for 5 min each with gentle agitation. The sections were incubated with 200 µl of 10% normal goat serum and 1% bovine serum albumin (BSA) in 1× TBS blocking solution per section for 2 h at room temperature. A mixture of desired primary antibodies was prepared with following dilutions: 1:1,000 recombinant Anti-Ki67 antibody (B56) and either 1:100 Aquaporin 1/AQP1 antibody (1/22), 1:1,000 Anti-Iba1 antibody (EPR16588), or 1:200 anti-alpha smooth muscle Actin antibody (SP171) with 1% BSA in 1× TBS. The sections were incubated with the primary antibody mixture in a humidifying chamber at 4 °C overnight. Slides were washed with two changes of 0.025% Triton X-100 in 1× TBS for 5 min each with gentle agitation. A mixture of secondary antibodies was prepared with following dilutions: 1:500 goat anti-mouse IgG (H + L) Highly Cross-Adsorbed Secondary Antibody Alexa Fluor Plus 555 (for use with Ki67 primary antibody) and either 1:500 goat anti-rabbit IgG (H + L) Highly Cross-Adsorbed Secondary Antibody Alexa Fluor 647 (for use with IBA1 or ACTA2 primary antibodies) or 1:500 goat anti-mouse IgG2b Cross-Adsorbed Secondary Antibody, Alexa Fluor 555 (for use with AQP1 primary antibody) with 1% BSA in 1× TBS. The sections were incubated with secondary antibodies for 1.5 h at room temperature in a dark staining chamber. Subsequently, the slide was washed with three changes of 1× TBS for 5 min each in a dark humidifying chamber. A stain mixture was prepared containing 2 μg ml^−1^ Hoechst 33258 and 1:200 Lectin HPA From Helix pomatia (edible snail) Alexa Fluor 488 Conjugate in 1× TBS. The sections were incubated with the stain mixture for 30 min at room temperature in a dark staining chamber. The slides were washed with 0.025% Triton X-100 in 1× TBS for 30 min and subsequently washed in three changes of 1× TBS for 5 min each at room temperature in a dark staining chamber. The slides were washed in 1× TBS for 30 min in a dark staining chamber, before mounting with ProLong Gold Antifade Mountant and a coverslip. The slides were left to solidify overnight in a dark staining chamber.

### Immunohistochemistry free-floating tissue

The supplier information and dilution used for each antibody is provided in Supplementary Table 11. Fresh meninges pieces were placed into a 12-well plate and fixed with 4% formaldehyde in 1× PBS for 10–15 min with gentle agitation. Subsequent steps were conducted as above but in a 12-well plate, with antibodies diluted in a volume of 1 ml per well to cover the tissue, and washed with 2 ml. Following the final washes, the floating meninges were placed on Superfrost Plus Adhesion Microscope Slides, and excess liquid was carefully removed. They were then mounted with ProLong Gold Antifade Mountant and a coverslip as above, and left to solidify overnight in a dark staining chamber

### Histology image acquisition

The supplier information and dilution used for each antibody is provided in Supplementary Table 11. Histology image acquisition was performed on a Zeiss Axio Scan.Z1 slide scanner with Zen Blue v3.1 acquisition software, using the Plan-Apochromat 20×/0.8 M27 objective with numerical aperture 0.8. For acquiring brightfield images (H&E staining), a Hitachi HV-F203SCL camera (pixel size of 4.4 µm) with a visible-light LED illumination source was used. For acquiring fluorescence images, a Hamamatsu ORCA-Flash4.0 V3 Digital CMOS camera (pixel size of 6.5 µm) with a Colibri 7 LED light source (100% intensity), multiband 405 + 493 + 575 + 654 + 761 beamsplitter and multi-bandpass 425/30 + 514/31 + 592/25 + 681/45 + 785/38 emission filters was used. Namely, the following configurations were used: H3258 channel (Hoechst stain) was imaged using LED-Module 385 nm and BP 385/30 excitation filter; AF488 channel (Lectin HPA Alexa Fluor 488 conjugate) was imaged using LED-Module 475 nm and BP 469/38 excitation filter; AF555 channel (goat anti-mouse IgG Highly Cross-Adsorbed Secondary Antibody, Alexa Fluor Plus 555, goat anti-mouse IgG2b Cross-Adsorbed Secondary Antibody, Alexa Fluor 555) was imaged using LED-Module 567 nm and BP 555/30 excitation filter; AF647 channel (goat anti-rabbit IgG Highly Cross-Adsorbed Secondary Antibody, Alexa Fluor 647) was imaged using LED-Module 630 nm and BP 631/33. Histology image acquisition was performed at Karolinska Institutet’s Biomedicum Imaging Core facility (https://ki.se/en/bic).

### Alignment of Xenium and histology images

H&E images are provided in the Xenium data folders (BioImage Archive, accession number S-BIAD1600). To align H&E files with the Xenium data, the appropriate .ome file and .csv alignment file need to be opened in the Xenium Browser.

### Statistics and reproducibility

No statistical method was used to predetermine sample size. Sample collection and processing was not intentionally randomized, but depended on the sequence of availability of human fetal tissue. No samples were excluded from analysis, and no data were excluded except where noted below (for example, doublet removal). The experiments were not randomized, and the investigators were not blinded during experiments and outcome assessment. Statistical testing (Fig. 6) used the Mann–Whitney *U* test, which does not assume a normal distribution.

### scRNA-seq data preprocessing

Illumina runs were demultiplexed with cellranger mkfastq version 6.1.2 (10x Genomics). Read mapping and UMI counts were determined using STARSolo^57^ version 2.7.10a, using human genome GRCh38.p12 and transcript annotations from ENSEMBL release 93 from reference package GRCh38-3.0.0 as available from 10Xgenomics (www.10xgenomics.com). STARSolo was run with the following parameters:

--soloType CB_UMI_Simple

--soloCellFilter EmptyDrops_CR <expectedNcells> 0.99 10 45000 90000 500 0.01 20000 0.01 10000

--soloCBmatchWLtype 1MM_multi_Nbase_pseudocounts

--soloUMIfiltering MultiGeneUMI_CR

--soloUMIdedup 1MM_CR

--clipAdapterType CellRanger4

--outFilterScoreMin 30

Replicates were pooled, resulting in one loom file (loompy.org) per sample.

### Quality control

Samples were analysed with ‘cytograph qc’ (https://github.com/linnarsson-lab/adult-human-brain), which uses a modified version of DoubletFinder to calculate a doublet score for each cell^58^. We captured a median 13,062 cells from each sample and then filtered cells on the basis of their total number of mRNA molecules—as counted by UMIs—and percentages of unspliced RNA, as well as doublet scores. ‘cytograph qc’ was initially run with standard parameters, where cells with fewer than 1,000 UMIs, unspliced molecule fraction less than 0.1, or a doublet score below 0.4 were removed from further analysis. A second round of quality control (QC) was performed after clustering (below). After QC, a final dataset containing 156,726 cells remained. Mean cells, genes and UMIs per sample are presented in Supplementary Table 1. Variability across donors probably reflected the quality of the tissue.

### Clustering

Cells were clustered using an updated version of the Cytograph package (https://github.com/linnarsson-lab/adult-human-brain). All cells that passed QC were pooled into a single dataset for initial clustering. The command ‘cytograph build’ was run with configuration factorization: HPF, nn_space: HPF, n_factors: 94, steps: nn, embeddings, clustering, aggregate, export. Default values were used for other parameters.

To obtain finer clusters, these top-level clusters were split by the dendrogram using the command ‘cytograph split --method dendrogram’. Fibroblasts, neural, and vascular cells were split one more time by the dendrogram such that no split had more than 37 clusters. A second round of more stringent QC was performed, whereby a cluster was removed if at least 40% of cells in the cluster had fewer than 1,800 UMIs, or if we manually spotted clusters containing doublets or *HBB* (erythrocyte) contamination. Then, the splits were pooled into final subsets; Subset_Neural, Subset_Neural_crest, Subset_Epithelial, Subset_Fibroblasts, Subset_Perivascular, Subset_Endothelial, Subset_Immune and Subset_Erythropoietic (Supplementary Table 2). Subset clusters were annotated and analysed separately, and pooled into a final complete dataset containing all 245 clusters (Supplementary Table 2; All_cells). Uniform Manifold Approximation and Projection (UMAP) embeddings were calculated for each subset and the pooled dataset of all cells.

### Annotation

Clusters were manually annotated as CellType based on literature, regional and age composition (Supplementary Table 2). Three additional levels of annotations were provided, on broader levels; ‘subclass’, ‘class’ and ‘superclass’. We computed cell cycle scores as previously described^43^. We used the expression of a set of well-known cell cycle genes^59^ as a proxy for active proliferation. We calculated the cell cycle score as the fraction of UMIs those genes represented, and then the percentage of cells per cluster with a cell cycle score >0.01. We also computed gene enrichment as previously described^43^, and available in deposited Jupyter Notebooks (https://github.com/linnarsson-lab/human-meninges-development). In brief, gene enrichment is a measure of overexpression in a cluster relative to other clusters, taking into account both mean expression and fraction of non-zero cells.

### Maternal calling by SNP analysis

We used Souporcell as created by Heaton et al.^21^, and freely available at https://github.com/wheaton5/souporcell, applying it to the output from the STARSolo runs. Souporcell uses variants detected in scRNA-seq reads to assign cells to their donor of origin.

### Processing of Xenium’s spatial datasets

Four types of sample were profiled: (1) developmental human meninges (PCW 6), (2) developmental human meninges (PCW 9.5), (2) adult human dura and (3) meningioma tumours (more details in Supplementary Table 1). Due to the differences expected between each of the sample types, each of the sample types was processed independently using Scanpy (see ‘Code availability’ section).

#### Processing of developmental and adult meningeal Xenium datasets

For developmental (PCW 6 and PCW 9.5) heads and adult dura samples, cells were first loaded and combined into a single AnnData object. With the aim of minimizing the segmentation errors caused by Xenium’s segmentation pipeline, only transcripts identified within segmented nuclei were kept for each of the profiled cells. This approach discards a considerable amount of the profiled reads but minimizes potential mis-segmentation effects^60^. General segmentation caveats have been reviewed elsewhere^61^. Only transcripts annotated as genes in the provided panel were retained. Cells were then filtered to remove low-quality observations, discarding cells with fewer than 8 detected genes or fewer than 20 total transcripts. After this, expression values were normalized to a total count of 10,000 transcripts per cell, followed by logarithmic transformation. Highly variable genes were identified based on specific thresholds of mean expression and dispersion, and principal component analysis was performed to reduce dimensionality. The neighbourhood graph was constructed using the top 50 principal components, and clustering was conducted using the Leiden algorithm. A UMAP 2D representation was generated to visualize cell clusters, and the processed data were saved. Based on the spatial location and differentially expressed genes identified for every cluster, clusters were annotated assigning a cell type to each high-quality cell profiled. For developmental datasets (PCW 6 and PCW 9.5), due to the complexity of both mesenchymal and neural clusters, these clusters were independently subclustered in a subsequent analysis (Code availability), integrating the resulting annotations into a final object containing a cell type assigned to each high-quality cell profiled.

#### Processing of meningioma Xenium datasets

Due to high biological variability between tumour samples, they were processed on a tumour basis, combining only replicates of the same tumour when available. QC measures were applied to filter out cells with fewer than three detected genes or fewer than five total transcripts. Expression data were normalized to a total count of 10,000 transcripts per cell, followed by a logarithmic transformation. Principal component analysis was used to reduce dimensionality, with the top 50 principal components selected to capture major sources of variation in the dataset. A neighbourhood graph was constructed using the top 40 components, enabling (1) the application of the Leiden algorithm to identify clusters and (2) a low-dimensional representation of the data, using UMAP.

### Banksy domain identification in Xenium datasets

BANKSY was used to identify spatially distinct tissue domains. Analysis was performed by grouping sections into independent batches based on developmental stage or, for tumour samples, by individual donor. Within each batch, multiple sections were aligned along the *x* axis with a 2,000-unit buffer to create a non-overlapping, unified spatial coordinate system. Spatial feature augmentation was performed by initializing a neighbourhood graph with 15 nearest neighbours using a scaled Gaussian decay kernel. We applied the BANKSY multiparametric clustering approach with a mixing parameter of = 1, a spatial weighting lambda of 1.0, and a clustering resolution of 0.4. The resulting spatial domain assignments were then integrated into the original metadata.

### B-lineage analysis

We produced dotplots to visualize gene expression, using raw gene expression count matrices subset to cell types of interest as input. We then normalized these count matrices using the Scanpy (v1.9.3) sc.pp.normalise_per_cell function with counts_per_cell_after = 1e4. A ln(*x* + 1) transformation was then applied to the normalized counts using the sc.pp.log1p function. Gene expression was then visualized using the sc.pl.dotplot function. To increase interpretability of plots, the vmax argument was sometimes used to apply an upper limit to the colour scale (where used, noted within GitHub scripts). Data produced in this study were compared with data from fetal bone marrow as B cell ref. ^24^ and yolk sac^23^.

### RNA velocity analysis

scVelo (Bergen et al.^62^) version 0.3.3 and scanpy version 1.12 were installed in February 2026, along with accompanying packages as per maintainers instructions (https://scvelo.readthedocs.io/en/stable/installation.html). For preprocessing, we used the commands pp.filter_and_normalize(adata, min_shared_counts = 20, n_top_genes = 2000), pp.moments(adata, n_pcs = 30, n_neighbors = 30), and generated velocity graphs with default settings using tl.velocity and tl.velocity_graph.

### CSF proteome analysis

Proteomics from human CSF was acquired from ref. ^39^ (Supplementary Table 3, ‘CSF minus plasma’) and from ref. ^38^ (CSF proteome resource: Supplementary Table 5, ‘Peptides glyco and non-glyco’). The lists were combined into a total of 272 proteins. The equivalent genes were identified, and 23 genes not expressed in fetal epithelial cells were removed. A collective CSF gene-set score was calculated with the remaining genes as described below (Neftel score adaptation).

### Neftel score

We independently re-implemented the gene profile scoring algorithm from Neftel et al.^63^, and generalized it to work with any given set of genes. Our implementation in Python is provided as neftel_score.py, and we confirmed that our code reproduces the original analysis from Neftel et al. Note that this is a non-deterministic algorithm, and the results that we presented in Fig. 7, Extended Data Fig. 10, and their respective source data are from an average of ten runs per analysis.

### Fibroblast layer-maturation score

Fibroblast data from the snRNA-seq data of Siletti et al.^12^ were downloaded, and the top 50 enriched genes per meningeal layer were calculated. The full pipeline is available in our accompanying GitHub page, Jupyter Notebook ‘Fig4_SFig4_and_layer_score_Fig7.ipynb’. In brief, cells were grouped by meningeal layer using label encoding, and for each gene we computed (1) the mean expression and (2) the fraction of non-zero cells per layer using grouped aggregation. An enrichment score was then calculated per gene and layer as the product of the ratio of in-layer versus out-of-layer non-zero fractions and mean expression (with a small pseudocount), where out-of-layer values were computed as weighted averages of all other layers. Genes were ranked by this enrichment score for each layer, and the top 50 genes per layer were selected. Next, three criteria were imposed to filter the gene lists such that they contained genes that are highly expressed, and only after PCW 6.

1. To be expressed in PCW 5–6: >15% of cells should have at least one transcript of the gene. If expressed in PCW 5–6, these genes were removed so that the gene list reflects genes involved in maturation of the meningeal layers.
2. To be expressed: gene count >2.
3. To be considered pia/arachnoid/dura: at least 5% of the enriched genes should be expressed in a cell to not be classified as ‘Other’.

### STRING network and pathways analysis

The gene expression values of a list of bioactive molecules, and enriched genes, for the inner dura and arachnoid barrier clusters were analysed and visualized using Cytoscape version 3.10.1^64^. The expression of bioactive molecules and enriched genes was analysed (1) as uniquely expressed by the inner dura or arachnoid barrier, and (2) as shared between the two. STRING database^65^ version 2.2.0 was used to visualize networks of interconnected molecules (50% confidence for node connectivity), belonging to significantly enriched pathway ontologies (Supplementary Table 6). Pathway analysis in STRING is based on KEGG, Reactome and Wiki pathways.

### Topic modelling with LDA

LDA^44^ was used to identify transcriptional modules that vary across cells, and also reveals co-expressed genes in single-cell data. We used tomotopy version 0.12.2 to perform topic modelling with LDA. Model training, identification of topic-representative genes, and other general parameters were performed as previously described^12^. Here, the number of topics ‘*k*’ generated were a range of 25, 30 and 35. Some topics were specific to quality, for example, *HBB* erythrocyte contamination, mitochondrial or ribosomal genes, and one topic contained genes from the Y chromosome. Some topics were not specific or did not seem to be biologically meaningful. Therefore, we manually chose topics of interest, based on those active in meningeal layers. At 35 topics, the dura topic split into an inner dura topic and dura topic, while those active in barrier, arachnoid precursors and arachnoid cells did not change. Therefore, we picked *k* = 35 and did not generate more. We identified representative genes for each topic by using the topic probabilities reported for each gene (function ‘get_topic_word_dist’). We filtered genes by specificity (topic probabilities for each gene normalized by that gene’s probability across all topics) and then sorted the remaining genes by their unnormalized probabilities (Supplementary Table 7). A script is available as optimize_lda.py at github.com/linnarsson-lab/adult-human-brain, and scripts of its application in this study are available at https://github.com/linnarsson-lab/human-meninges-development.

#### Transfer of scRNA-seq LDA topics to Xenium in situ gene expression data

LDA also allows you to score new cells for a given gene topic. We validated the scRNA-seq topic annotations by transfer to spatial data. Topics from the model were transferred to matching genes in the Xenium dataset (one grade I and six grade III tumours), and the weights obtained used to colorize the in situ images.

#### Transfer of scRNA-seq LDA topics to bulk RNA-seq data

Bulk RNA-seq data of 185 meningiomas was downloaded from Choudhury et al.^19^ and split according to their methylation group; merlin-intact, immune-enriched and hypermitotic (grades I–III). For each tumour and tumour type, the relative contribution of each topic was calculated. For visualization, topics that were not biologically relevant or not expressed by any of the tumours were removed, and sums of those expressed were normalized to 1.0.

#### LDA topic modelling on tumour Xenium in situ gene expression data

Topics were generated as above with *k* = 35 shown, but for individual tumour samples. Topics, filtered genes and topic annotations are presented in Supplementary Table 8. The topics were transferred to PCW 9.5 fetal spatial data, and were annotated on the basis of filtered genes and fetal anatomy resemblance. We did not annotate topics without filtered genes (that is, genes defining the topic did not pass the filtering criteria), except a few cases where they could be annotated on the basis of non-neural fibroblast layer resemblance. Tumour topics that mapped outside the meninges (for example, cycling genes also in the ventricular zone) might represent general gene programs associated with stemness, maturation or migration. Note that some topics annotated as ‘immune’ aren’t necessarily cell type specific, but rather represent a functional program (for example, attracting T cells).

### Reanalysis of meningioma scRNA-seq data

The data were downloaded from the work of Choudhury et al.^19^ and processed with the same Cytograph pipeline as described for the fetal meninges scRNA-seq data generated in this study. Tumour cells (fibroblast-like) and pericytes were pooled into a new loom file, as with the fetal meningeal fibroblasts and pericytes. This dataset contained two grade I tumours, three grade II tumours and one grade III tumour. Harmony batch correction on the tumour scRNA-seq data was not used, as we decided to proceed without it to preserve biological heterogeneity and to keep the comparison consistent with the fetal data.

### Computational expression-based karyotyping

We inferred chromosomal copy number aberrations by determining normalized gene expression along chromosomes, using a normal brain atlas^12^ as the reference. By using an external reference, we avoided the need to manually identify normal cells within each sample. We normalized expression in each cell to the median total UMIs across all cells. We included only genes from the autosomes that were expressed at more than 10% of the 99th percentile expression level in at least half of the clusters in the reference atlas. Then, for each cell in the sample, we identified the reference cell type (cluster in the atlas) with the greatest correlation coefficient. For each cell, we then computed a ploidy score along the chromosomes by dividing the normalized expression in the cell by the normalized expression in the reference cell type and multiplying by two. We smoothed the ploidy along the chromosomes in windows of 25 genes and then normalized the resulting ploidy to the median ploidy and multiplied by two. We used the resulting ploidy vectors (one for each cell in the sample) as the input for a clustering procedure: we computed the first five principal components, calculated a nearest-neighbour graph (*k* = 30 neighbours) and used Leiden graph clustering to find metacells having similar ploidy profiles. Finally, for each metacell, we used a hidden Markov model to infer integer chromosomal copy numbers. The detailed Python code implementing this algorithm is provided as hmm_karyotyper.py, which also provides visualization of the result.

### Expression of COSMIC-mutated genes in meningeal fibroblasts

We reasoned that genes frequently mutated in a tumour must often be expressed in the cell of origin, before oncogenic transformation. For example, a tumour suppressor would be expressed in the cell of origin, and its loss by for example deletion would drive tumorigenesis. On the other hand, a tumour suppressor gene that is not expressed in the cell of origin would not drive tumorigenesis even if mutated. Similarly, a proto-oncogene would be expressed in the cell of origin, but its expression might be increased by copy number amplification, driving tumour growth. However, if it is not expressed, amplification would not increase expression. Again, similarly, a mutation in a proto-oncogene protein will drive tumorigenesis only if the proto-oncogene is expressed; if it is not, the mutation cannot have any effect. As an exception, the telomerase gene *TERT* is a driver gene not expressed in somatic cells. It is commonly induced de novo by activating promoter mutations and, hence, not expected to be expressed in the cell of origin.

The Catalogue of Somatic Mutations in Cancer (COSMIC) Cancer Browser (https://cancer.sanger.ac.uk/cosmic/browse/tissue) version 98-99 was used to find the top 20 mutated genes in meningioma, glioblastoma (astrocytoma grade IV), intestinal carcinoma and prostate adenocarcinoma. Overlapping genes between cancers were removed, to ensure that generic cancer drivers did not have an effect (Supplementary Table 10). The telomerase gene *TERT* was removed, but including it in the analysis does not change the result because it is not normally expressed in somatic cells.

### Reporting summary

Further information on research design is available in the Nature Portfolio Reporting Summary linked to this article.

## Online content

Any methods, additional references, Nature Portfolio reporting summaries, source data, extended data, supplementary information, acknowledgements, peer review information; details of author contributions and competing interests; and statements of data and code availability are available at https://doi.org/10.1038/s41556-026-02074-9.

## Supplementary information

Reporting Summary

Peer Review File

Supplementary Table 1 Samples.

Supplementary Table 2 scRNA-seq cluster metadata.

Supplementary Table 3 Xenium probe panels.

Supplementary Table 4 Xenium clusters.

Supplementary Table 5 CSF analysis, related to Supplementary Fig. 3.

Supplementary Table 6 Network analysis, related to Figs. 5 and 6.

Supplementary Table 7 scRNA-seq topics, related to Figs. 6 and 7.

Supplementary Table 8 Spatial topics in tumours, related to Supplementary Fig. 8.

Supplementary Table 9 Spatial domain detection, related to Supplementary Figs. 4 and 9.

Supplementary Table 10 COSMIC-mutated genes, related to Fig. 7 and Supplementary Fig. 10.

Supplementary Table 11 Reagents and resources, related to the Methods.

## Source data

Source Data Fig. 1 Source data for graphs.

Source Data Fig. 2 Source data for graphs.

Source Data Fig. 3 Source data for graphs.

Source Data Fig. 4 Source data for graphs.

Source Data Fig. 5 and Extended Data Fig. 5 Source data for graphs.

Source Data Fig. 6 Statistical source data.

Source Data Fig. 7 Source data for graphs.

Source Data Extended Data Fig. 2 Source data for graphs.

Source Data Extended Data Fig. 7 Source data for graphs.

Source Data Extended Data Fig. 8 Source data for graphs.

Source Data Extended Data Fig. 10 Source data for graphs.

## Data Availability

Source data are provided with this paper. All other data supporting the findings of this study are available from the corresponding author on reasonable request. Sequencing data that support the findings of this study have been deposited in the European Genome/Phenome Archive (https://ega-archive.org/) under accession code EGAD50000001434. Count matrices are available via GitHub at https://github.com/linnarsson-lab/human-meninges-development. A permanent archived version of the repository is available via Zenodo at https://doi.org/10.5281/zenodo.21356807 (ref. ^66^). Xenium in situ data and images have been deposited in the BioImage Archive under accession code S-BIAD1600 (https://www.ebi.ac.uk/bioimage-archive/). Previously published sequencing data that were re-analysed here are available at NCBI Gene Expression Omnibus under accession code GSE183656.

## Code availability

All original code for the analysis and visualization of data, and all code needed to reproduce all figures, is available via GitHub at https://github.com/linnarsson-lab/human-meninges-development, without any restrictions to access. A permanent archived version is available via Zenodo at https://doi.org/10.5281/zenodo.21356807 (ref. ^66^).

## References

[1] Aldinger, K. A. et al. FOXC1 is required for normal cerebellar development and is a major contributor to chromosome 6p25.3 Dandy-Walker malformation. *Nat. Genet.* **41**, 1037–1042 (2009). 10.1038/ng.422 PMC2843139 19668217

[2] Brastianos, P. K. et al. Advances in multidisciplinary therapy for meningiomas. *Neuro. Oncol.* **21**, i18–i31 (2019). 10.1093/neuonc/noy136 PMC6347080 30649489

[3] Cleland, J. Description of two tumours adherent to the deep surface of the dura mater. *Glasg. Med. J.* **11**, 148–159 (1864). PMC5801940 30432487

[4] Schmidt, M. B. Ueber die Pacchioni’schen Granulationen und ihr Verhältniss zu den Sarcomen und Psammomen der Dura mater. *Archiv für pathologische Anatomie und Physiologie und für klinische Medicin (Virchow’s Achiv)* **170**, 429–464 (1902).

[5] Bailey, P. & Bucy, P. C. The origin and nature of meningeal tumors. *Am. J. Cancer* **15**, 15–54 (1931).

[6] WHO Classification of Tumours Editorial Board. *Central Nervous System Tumours* Vol. 6 (International Agency for Research on Cancer, 2021).

[7] Gold, M. P. et al. Developmental basis of SHH medulloblastoma heterogeneity. *Nat. Commun.* **15**, 270 (2024). 10.1038/s41467-023-44300-0 PMC10774283 38191555

[8] DeSisto, J. et al. Single-cell transcriptomic analyses of the developing meninges reveal meningeal fibroblast diversity and function. *Dev. Cell* **54**, 43–59 (2020). 10.1016/j.devcel.2020.06.009 PMC7769050 32634398

[9] Wang, A. Z. et al. Single-cell profiling of human dura and meningioma reveals cellular meningeal landscape and insights into meningioma immune response. *Genome Med.* **14**, 49 (2022). 10.1186/s13073-022-01051-9 PMC9088131 35534852

[10] Pietilä, R. et al. Molecular anatomy of adult mouse leptomeninges. *Neuron* **111**, 3745–3764 (2023). 10.1016/j.neuron.2023.09.002 37776854

[11] Kearns, N. A. et al. Dissecting the human leptomeninges at single-cell resolution. *Nat. Commun.* **14**, 7036 (2023). 10.1038/s41467-023-42825-y PMC10624900 37923721

[12] Siletti, K. et al. Transcriptomic diversity of cell types across the adult human brain. *Science* **382**, eadd7046 (2023). 10.1126/science.add7046 37824663

[13] Braun, E. et al. Comprehensive cell atlas of the first-trimester developing human brain. *Science* **382**, eadf1226 (2023). 10.1126/science.adf1226 37824650

[14] Farmer, D. T. et al. The developing mouse coronal suture at single-cell resolution. *Nat. Commun.* **12**, 4797 (2021). 10.1038/s41467-021-24917-9 PMC8355337 34376651

[15] Li, B. et al. Dissecting calvarial bones and sutures at single-cell resolution. *Biol. Rev. Camb. Philos. Soc.* **98**, 1749–1767 (2023). 10.1111/brv.12975 37171117

[16] Sankowski, R. et al. Multiomic spatial landscape of innate immune cells at human central nervous system borders. *Nat. Med.* **30**, 186–198 (2024). 10.1038/s41591-023-02673-1 PMC10803260 38123840

[17] Mortus, J. R., Zhang, Y. & Hughes, D. P. M. Developmental pathways hijacked by osteosarcoma. *Adv. Exp. Med. Biol.* **804**, 93–118 (2014). 10.1007/978-3-319-04843-7_5 24924170

[18] Okano, A. et al. Associations of pathological diagnosis and genetic abnormalities in meningiomas with the embryological origins of the meninges. *Sci. Rep.* **11**, 6987 (2021). 10.1038/s41598-021-86298-9 PMC7998008 33772057

[19] Choudhury, A. et al. Meningioma DNA methylation groups identify biological drivers and therapeutic vulnerabilities. *Nat. Genet.* **54**, 649–659 (2022). 10.1038/s41588-022-01061-8 PMC9374001 35534562

[20] To, K. et al. Spatially resolved multiomics of human meninges development reveal lineage and disease dynamics. *Nat. Cell Biol.* 10.1038/s41556-026-02075-8 (2026). PMC13661283 42806090

[21] Heaton, H. et al. Souporcell: robust clustering of single-cell RNA-seq data by genotype without reference genotypes. *Nat. Methods* **17**, 615–620 (2020). 10.1038/s41592-020-0820-1 PMC7617080 32366989

[22] O’Byrne, S. et al. Discovery of a CD10-negative B-progenitor in human fetal life identifies unique ontogeny-related developmental programs. *Blood* **134**, 1059–1071 (2019). 10.1182/blood.2019001289 31383639

[23] Goh, I. et al. Yolk sac cell atlas reveals multiorgan functions during human early development. *Science* **381**, eadd7564 (2023). 10.1126/science.add7564 PMC7614978 37590359

[24] Jardine, L. et al. Blood and immune development in human fetal bone marrow and Down syndrome. *Nature* **598**, 327–331 (2021). 10.1038/s41586-021-03929-x PMC7612688 34588693

[25] Vanlandewijck, M. et al. A molecular atlas of cell types and zonation in the brain vasculature. *Nature* **554**, 475–480 (2018). 10.1038/nature25739 29443965

[26] Kalucka, J. et al. Single-cell transcriptome atlas of murine endothelial cells. *Cell* **180**, 764–779 (2020). 10.1016/j.cell.2020.01.015 32059779

[27] Cao, J. et al. A human cell atlas of fetal gene expression. *Science* **370**, eaba7721 (2020). 10.1126/science.aba7721 PMC7780123 33184181

[28] Winkler, E. A. et al. A single-cell atlas of the normal and malformed human brain vasculature. *Science* **375**, eabi7377 (2022). 10.1126/science.abi7377 PMC8995178 35084939

[29] Hupe, M. et al. Gene expression profiles of brain endothelial cells during embryonic development at bulk and single-cell levels. *Sci. Signal.* **10**, eaag2476 (2017). 10.1126/scisignal.aag2476 28698213

[30] Jain, A., Ang, P. S., Matrongolo, M. J. & Tischfield, M. A. Understanding the development, pathogenesis, and injury response of meningeal lymphatic networks through the use of animal models. *Cell. Mol. Life Sci.* **80**, 332 (2023). 10.1007/s00018-023-04984-5 PMC11072018 37872442

[31] Wilting, J. et al. The transcription factor Prox1 is a marker for lymphatic endothelial cells in normal and diseased human tissues. *FASEB J.* **16**, 1271–1273 (2002). 10.1096/fj.01-1010fje 12060670

[32] Wigle, J. T. et al. An essential role for Prox1 in the induction of the lymphatic endothelial cell phenotype. *EMBO J.* **21**, 1505–1513 (2002). 10.1093/emboj/21.7.1505 PMC125938 11927535

[33] Jung, E. et al. Development and characterization of a novel Prox1–EGFP lymphatic and Schlemm’s canal reporter rat. *Sci. Rep.* **7**, 5577 (2017). 10.1038/s41598-017-06031-3 PMC5514086 28717161

[34] Antila, S. et al. Development and plasticity of meningeal lymphatic vessels. *J. Exp. Med.* **214**, 3645–3667 (2017). 10.1084/jem.20170391 PMC5716035 29141865

[35] Dani, N. et al. A cellular and spatial map of the choroid plexus across brain ventricles and ages. *Cell* **184**, 3056–3074 (2021). 10.1016/j.cell.2021.04.003 PMC8214809 33932339

[36] Guldbrandsen, A. et al. In-depth characterization of the cerebrospinal fluid (CSF) proteome displayed through the CSF proteome resource (CSF-PR). *Mol. Cell. Proteomics* **13**, 3152–3163 (2014). 10.1074/mcp.M114.038554 PMC4223498 25038066

[37] Dayon, L. et al. Proteomes of paired human cerebrospinal fluid and plasma: relation to blood–brain barrier permeability in older adults. *J. Proteome Res.* **18**, 1162–1174 (2019). 10.1021/acs.jproteome.8b00809 30702894

[38] Mosby, L. S., Bowen, A. E. & Hadjivasiliou, Z. Morphogens in the evolution of size, shape and patterning. *Development* **151**, dev202412 (2024). 10.1242/dev.202412 PMC7616732 39302048

[39] Zhu, X.-J. et al. BMP-FGF signaling axis mediates Wnt-induced epidermal stratification in developing mammalian skin. *PLoS Genet.* **10**, e1004687 (2014). 10.1371/journal.pgen.1004687 PMC4199507 25329657

[40] Betsholtz, C. et al. Advances and controversies in meningeal biology. *Nat. Neurosci.* **27**, 2056–2072 (2024). 10.1038/s41593-024-01701-8 PMC11862877 39333784

[41] Peyre, M. et al. PDGF activation in PGDS-positive arachnoid cells induces meningioma formation in mice promoting tumor progression in combination with Nf2 and Cdkn2ab loss. *Oncotarget* **6**, 32713–32722 (2015). 10.18632/oncotarget.5296 PMC4741724 26418719

[42] Choudhury, A. et al. Perivascular NOTCH3^+^ stem cells drive meningioma tumorigenesis and resistance to radiotherapy. *Cancer Discov.* **14**, 1823–1837 (2024). 10.1158/2159-8290.CD-23-1459 PMC11452293 38742767

[43] La Manno, G. et al. Molecular architecture of the developing mouse brain. *Nature* **596**, 92–96 (2021). 10.1038/s41586-021-03775-x 34321664

[44] Blei, D. M., Ng, A. Y. & Jordan, M. I. Latent Dirichlet allocation. *J. Mach. Learn. Res.* **3**, 993–1022 (2003).

[45] LeBleu, V. S. & Neilson, E. G. Origin and functional heterogeneity of fibroblasts. *FASEB J.* **34**, 3519–3536 (2020). 10.1096/fj.201903188R 32037627

[46] Plikus, M. V. et al. Fibroblasts: origins, definitions, and functions in health and disease. *Cell* **184**, 3852–3872 (2021). 10.1016/j.cell.2021.06.024 PMC8566693 34297930

[47] Derk, J. et al. Formation and function of the meningeal arachnoid barrier around the developing mouse brain. *Dev. Cell* **58**, 635–644 (2023). 10.1016/j.devcel.2023.03.005 PMC10231667 36996816

[48] Nassiri, F. et al. A clinically applicable integrative molecular classification of meningiomas. *Nature* **597**, 119–125 (2021). 10.1038/s41586-021-03850-3 PMC11604310 34433969

[49] Driver, J. et al. A molecularly integrated grade for meningioma. *Neuro. Oncol.* **24**, 796–808 (2022). 10.1093/neuonc/noab213 PMC9071299 34508644

[50] Chen, W. C. et al. Targeted gene expression profiling predicts meningioma outcomes and radiotherapy responses. *Nat. Med.* **29**, 3067–3076 (2023). 10.1038/s41591-023-02586-z PMC11073469 37944590

[51] Lucas, C.-H. G. et al. Spatial genomic, biochemical and cellular mechanisms underlying meningioma heterogeneity and evolution. *Nat. Genet.* **56**, 1121–1133 (2024). 10.1038/s41588-024-01747-1 PMC11239374 38760638

[52] Yamashima, T., Kida, S. & Yamamoto, S. Ultrastructural comparison of arachnoid villi and meningiomas in man. *Mod. Pathol.* **1**, 224–234 (1988). 3237703

[53] Tohma, Y., Yamashima, T. & Yamashita, J. Immunohistochemical localization of cell adhesion molecule epithelial cadherin in human arachnoid villi and meningiomas. *Cancer Res.* **52**, 1981–1987 (1992). 1551127

[54] Kalamarides, M. et al. Identification of a progenitor cell of origin capable of generating diverse meningioma histological subtypes. *Oncogene* **30**, 2333–2344 (2011). 10.1038/onc.2010.609 21242963

[55] Boetto, J., Peyre, M. & Kalamarides, M. Mouse models in meningioma research: a systematic review. *Cancers* **13**, 3712 (2021). 10.3390/cancers13153712 PMC8345085 34359639

[56] Louis, D. N. et al. The 2021 WHO classification of tumors of the central nervous system: a summary. *Neuro. Oncol.* **23**, 1231–1251 (2021). 10.1093/neuonc/noab106 PMC8328013 34185076

[57] Dobin, A. et al. STAR: ultrafast universal RNA-seq aligner. *Bioinformatics* **29**, 15–21 (2013). 10.1093/bioinformatics/bts635 PMC3530905 23104886

[58] McGinnis, C. S., Murrow, L. M. & Gartner, Z. J. DoubletFinder: doublet detection in single-cell RNA sequencing data using artificial nearest neighbors. *Cell Syst.* **8**, 329–337 (2019). 10.1016/j.cels.2019.03.003 PMC6853612 30954475

[59] Tirosh, I. et al. Dissecting the multicellular ecosystem of metastatic melanoma by single-cell RNA-seq. *Science* **352**, 189–196 (2016). 10.1126/science.aad0501 PMC4944528 27124452

[60] Marco Salas, S., Kuemmerle, L.B. & Mattsson-Langseth, C. et al. Optimizing Xenium In Situ data utility by quality assessment and best-practice analysis workflows. *Nat. Methods* **22**, 813–823 (2025). 10.1038/s41592-025-02617-2 PMC11978515 40082609

[61] Mitchel, J., Gao, T., Petukhov, V., Cole, E. & Kharchenko, P. V. Impact and correction of segmentation errors in spatial transcriptomics. *Nat. Genet.* **58**, 434–444 (2026). 10.1038/s41588-025-02497-4 41559218

[62] Bergen, V., Lange, M., Peidli, S., Wolf, F. A. & Theis, F. J. Generalizing RNA velocity to transient cell states through dynamical modeling. *Nat. Biotechnol.* **38**, 1408–1414 (2020). 10.1038/s41587-020-0591-3 32747759

[63] Neftel, C. et al. An integrative model of cellular states, plasticity, and genetics for glioblastoma. *Cell* **178**, 835–849 (2019). 10.1016/j.cell.2019.06.024 PMC6703186 31327527

[64] Shannon, P. et al. Cytoscape: a software environment for integrated models of biomolecular interaction networks. *Genome Res.* **13**, 2498–2504 (2003). 10.1101/gr.1239303 PMC403769 14597658

[65] Szklarczyk, D. et al. The STRING database in 2023: protein-protein association networks and functional enrichment analyses for any sequenced genome of interest. *Nucleic Acids Res.* **51**, D638–D646 (2023). 10.1093/nar/gkac1000 PMC9825434 36370105

[66] Copied TextX Vinsland E. Lönnerberg P. Linnarsson S. linnarsson-lab/human-meninges-development: code accompanying Vinsland, et al. 2026 (version v2026-07-14) *Zenodo*10.5281/zenodo.21356807 (2026).

