# Supplementary material for: Cell atlas of the developing human meninges reveals a dura-like nature of meningiomas
Source: Nat Cell Biol. 2026 Sep 28;28(10):2155–68. doi: 10.1038/s41556-026-02074-9 (PMC13661240; doi:10.1038/s41556-026-02074-9)
Supplement: Supplementary file 1 [file 41556_2026_2074_MOESM1_ESM.pdf]

nature portfolio

Corresponding author(s): Elin Vinsland, Sten Linnarsson

Last updated by author(s): Jun 18, 2026

Reporting Summary

Nature Portfolio wishes to improve the reproducibility of the work that we publish. This form provides structure for consistency and transparency in reporting. For further information on Nature Portfolio policies, see our Editorial Policies and the Editorial Policy Checklist.

Statistics

For all statistical analyses, confirm that the following items are present in the figure legend, table legend, main text, or Methods section.

n/a	Confirmed	
☐	☒	The exact sample size (*n*) for each experimental group/condition, given as a discrete number and unit of measurement
☐	☒	A statement on whether measurements were taken from distinct samples or whether the same sample was measured repeatedly
☐	☒	The statistical test(s) used AND whether they are one- or two-sided<br>*Only common tests should be described solely by name; describe more complex techniques in the Methods section.*
☒	☐	A description of all covariates tested
☐	☒	A description of any assumptions or corrections, such as tests of normality and adjustment for multiple comparisons
☐	☒	A full description of the statistical parameters including central tendency (e.g. means) or other basic estimates (e.g. regression coefficient) AND variation (e.g. standard deviation) or associated estimates of uncertainty (e.g. confidence intervals)
☐	☒	For null hypothesis testing, the test statistic (e.g. *F*, *t*, *r*) with confidence intervals, effect sizes, degrees of freedom and *P* value noted<br>*Give P values as exact values whenever suitable.*
☒	☐	For Bayesian analysis, information on the choice of priors and Markov chain Monte Carlo settings
☒	☐	For hierarchical and complex designs, identification of the appropriate level for tests and full reporting of outcomes
☒	☐	Estimates of effect sizes (e.g. Cohen's *d*, Pearson's *r*), indicating how they were calculated

Our web collection on statistics for biologists contains articles on many of the points above.

Software and code

Policy information about availability of computer code

Data collection

All software and versions used have been reported in the Methods section of the manuscript.
Cytograph (Siletti et al).
https://github.com/linnarsson-lab/adult-human-brain/tree/main/cytograph
Souporcell (Heaton et al.)
https://github.com/wheaton5/souporcell
Tomotopy v0.12.2 (Blei et al.)
https://bab2min.github.io/tomotopy/v0.13.0/en/
STAR v2.7.10a (Dobin et al.)
https://github.com/alexdobin/STAR
CellRanger v6.1.2 (10X Genomics)
https://www.10xgenomics.com/support/software/cell-ranger/latest
Cytoscape v3.10.1 (Shannon et al.)
https://cytoscape.org
STRING v2.2.0 (Szklarczyk et al.)
https://www.string-db.org
Xenium data processing (Salas et al., this manuscript)
https://www.biorxiv.org/content/10.1101/2023.02.13.528102v1
scVelo 0.3.3 (Bergen et al.)
https://scvelo.readthedocs.io/en/stable/installation.html
Code for analysis and visualisation (This manuscript)

https://github.com/linnarsson-lab/human-meninges-development

Data analysis

All original code for the analysis and visualisation of data has been deposited to our companion GitHub page at https://github.com/linnarsson-lab/human-meninges-development.

For manuscripts utilizing custom algorithms or software that are central to the research but not yet described in published literature, software must be made available to editors and reviewers. We strongly encourage code deposition in a community repository (e.g. GitHub). See the Nature Portfolio guidelines for submitting code & software for further information.

Data

Policy information about availability of data

All manuscripts must include a data availability statement. This statement should provide the following information, where applicable:

- Accession codes, unique identifiers, or web links for publicly available datasets
- A description of any restrictions on data availability
- For clinical datasets or third party data, please ensure that the statement adheres to our policy

scRNA-seq: BAM files are available from the European Genome/Phenome Archive (https://ega-archive.org/) under accession number EGAD50000001434. Accessing the data requires signing a data access agreement to rpotect the privacy of donors under GDPR.
Count matrices are available from our companion GitHub page at https://github.com/linnarsson-lab/human-meninges-development.
Xenium in situ data and images are available from the BioImage Archive under accession number S-BIAD1600 (https://www.ebi.ac.uk/bioimage-archive/).

Research involving human participants, their data, or biological material

Policy information about studies with human participants or human data. See also policy information about sex, gender (identity/presentation), and sexual orientation and race, ethnicity and racism.

Reporting on sex and gender

As our fetal samples were collected and processed as soon as possible, the sex of the sample could not be determined ahead of time, as such we collected from both sexes. Sex was reported for the meningioma and dura samples and can be found in Supplementary table 1. Sex/Gender was not considered in this manuscript as we collected samples from both sexes and we do not expect it to have a significant impact on our findings.

Reporting on race, ethnicity, or other socially relevant groupings

Samples were collected anonymously from donation in Sweden, the United Kingdom, and the US. The donation procedures did not allow us to collect race or ethnicity or similar information about the donors. As such we do not know if the ethnic backgrounds of the samples are broadly representative of the general population.

Population characteristics

See above.

Recruitment

Donors were recruited as part of routine health care, upon receiving a meningioma diagnosis, or upon electing to perform an abortion.

Ethics oversight

Human prenatal samples were collected from elective medical abortions at the Department of Gynecology, Danderyd Hospital and Karolinska Huddinge Hospital, and Addenbrooke's Hospital in Cambridge, following oral and written informed consent by the patient. In Sweden, the use of abortion material was approved by the Swedish Ethical Review Authority and the National Board of Health and Welfare. In the UK, approval from the National Research Ethics Service Committee East of England – Cambridge Central was obtained (Local Research Ethics Committee, 96/085).

Human dura samples and one meningioma sample ("SL123") were collected from the Karolinska Hospital with informed consent from the patients and with ethical approval from the Swedish Ethical Review Authority (2020-03505). The use of samples was approved by the Swedish Ethical Review Authority (2020-02096).

Meningioma samples were collected from the University of California San Francisco (UCSF), complied with all relevant ethical regulations, and was approved by the UCSF Institutional Review Board (13–12587, 17–22324, 17–23196 and 18–24633). As part of routine clinical practice at UCSF, all patients who were included in this study signed a written waiver of informed consent to contribute deidentified data to research projects.

Note that full information on the approval of the study protocol must also be provided in the manuscript.

Field-specific reporting

Please select the one below that is the best fit for your research. If you are not sure, read the appropriate sections before making your selection.

☒ Life sciences ☐ Behavioural & social sciences ☐ Ecological, evolutionary & environmental sciences

For a reference copy of the document with all sections, see nature.com/documents/nr-reporting-summary-flat.pdf

Life sciences study design

All studies must disclose on these points even when the disclosure is negative.

Sample size	The study is exploratory, focusing on an atlas of the developing meninges. Due to the nature of human fetal samples, it was not possible to include a fixed number of samples per timepoint. Rather, a sufficent number of samples was included to ensure deep sampling of developmental lineages where no single human sample dominated. These considerations were based on prior experience. There is no accepted minimum or maximum sample size for such a cell atlas; generally the more samples are included, the better the resolution.
Data exclusions	No samples were excluded
Replication	Single-cell data was validated by extensive spatial transcriptomics data at two timepoints, as well as targeted validation by immunohistochemistry as indicated. Some pre-natal ages could not be replicated by more than one sample, because we do not determine the ages of human material that can be received, therefore, it is difficult to replicate within specific time frames of the project.
Randomization	N/A
Blinding	We were blinded to the donor patient information due to confidentiality and anonymity around abortions. Blinding of the age of the fetal samples was not possible, since we needed to know the age for analysis.

Reporting for specific materials, systems and methods

We require information from authors about some types of materials, experimental systems and methods used in many studies. Here, indicate whether each material, system or method listed is relevant to your study. If you are not sure if a list item applies to your research, read the appropriate section before selecting a response.

Materials & experimental systems

n/a	Involved in the study	
☐	☒	Antibodies
☒	☐	Eukaryotic cell lines
☒	☐	Palaeontology and archaeology
☒	☐	Animals and other organisms
☒	☐	Clinical data
☒	☐	Dual use research of concern
☒	☐	Plants

Methods

n/a	Involved in the study	
☒	☐	ChIP-seq
☒	☐	Flow cytometry
☒	☐	MRI-based neuroimaging

Antibodies

Antibodies used	Recombinant Anti-Ki67 antibody [B56] Abcam Cat# ab279653, RRID: AB_2934265<br>Aquaporin 1/AQP1 Antibody (1/22) Santa Cruz Biotechnology Cat# sc-32737, RRID: AB_626693<br>Anti-Iba1 antibody [EPR16588] Abcam Cat# ab178846, RRID: AB_2636859<br>Anti-alpha smooth muscle Actin antibody [SP171] Abcam Cat# ab150301<br>Goat anti-Mouse IgG (H+L) Highly Cross-Adsorbed Secondary Antibody, Alexa Fluor™ Plus 555 Thermo Fisher Scientific Cat# A32727, RRID: AB_2633276<br>Goat anti-Rabbit IgG (H+L) Highly Cross-Adsorbed Secondary Antibody, Alexa Fluor™ 647 Thermo Fisher Scientific Cat# A-21245, RRID: AB_2535813<br>Goat anti-Mouse IgG2b Cross-Adsorbed Secondary Antibody, Alexa Fluor™ 555 Thermo Fisher Scientific Cat# A-21147, RRID: AB_2535783
Validation	According to manufacturer's website

Plants

Seed stocks	*Report on the source of all seed stocks or other plant material used. If applicable, state the seed stock centre and catalogue number. If plant specimens were collected from the field, describe the collection location, date and sampling procedures.*
Novel plant genotypes	*Describe the methods by which all novel plant genotypes were produced. This includes those generated by transgenic approaches, gene editing, chemical/radiation-based mutagenesis and hybridization. For transgenic lines, describe the transformation method, the number of independent lines analyzed and the generation upon which experiments were performed. For gene-edited lines, describe the editor used, the endogenous sequence targeted for editing, the targeting guide RNA sequence (if applicable) and how the editor was applied.*
Authentication	*Describe any authentication procedures for each seed stock used or novel genotype generated. Describe any experiments used to assess the effect of a mutation and, where applicable, how potential secondary effects (e.g. second site T-DNA insertions, mosiacism, off-target gene editing) were examined.*
