# Supplementary material for: Cell atlas of the developing human meninges reveals a dura-like nature of meningiomas
Source: Nat Cell Biol. 2026 Sep 28;28(10):2155–68. doi: 10.1038/s41556-026-02074-9 (PMC13661240; doi:10.1038/s41556-026-02074-9)

nature portfolio

Peer Review File

Cell atlas of the developing human meninges reveals a dura-like nature of meningiomas

Corresponding Author: Professor Sten Linnarsson

Version 0:

Decision Letter:

*Please delete the link to your author homepage if you wish to forward this email to co-authors.

Dear Sten,

Your manuscript, "Cell atlas of the developing human meninges reveals a dura origin of meningioma", has now been seen by 4 referees: an expert in (human) brain development (referee 1); an expert in single-cell and spatial transcriptomics, cancer, computational biology (referee 2); referee 3, who co-reviewed the manuscript together with referee 2; and an expert in brain cancer, genomics (referee 4). As you will see from their comments (attached below) they find this work of potential interest, but have raised substantial concerns, which in our view would need to be addressed with considerable revisions before we can consider publication in Nature Cell Biology.

Nature Cell Biology editors discuss the referee reports in detail within the editorial team, including the chief editor, to identify key referee points that should be addressed with priority. To guide the scope of the revisions, I have listed these points below. We are committed to providing a fair and constructive peer-review process, so please feel free to contact me if you would like to discuss any of the referee comments further.

In particular, it would be essential to:

(A) Provide further analyses, clarifications, discussion, and thorough reporting as requested by reviewers #2, #3 and #4 in the following points:

Reviewers #2 and #3:
"Sample metadata and donor characteristics require further clarification. This study includes samples from 13 donors across 5–13 PCW, but additional details on donor demographics, biological variance, and potential confounders (e.g., gestational age precision, maternal health, tissue procurement factors) would enhance interpretability. As this atlas will serve as a foundational resource, transparent metadata reporting is essential for future reanalysis and integration."

"Quality control, batch correction, and computational pipeline details should be more fully described. Given the multi-sample, multi-platform nature of the dataset, clear documentation of QC thresholds, filtering criteria, and how batch effects were mitigated is critical for reproducibility."

"Spatial validation of fibroblast and barrier-like layers could be expanded. The discovery of an inner dura layer expressing barrier genes is interesting, but appears to rely heavily on selected spatial slices. A more systematic description of spatial reproducibility across donors, sections, and developmental stages would increase confidence in this structural finding, especially since it affects the proposed disease-origin model."

"Integration of tumor samples would benefit from more detailed explanation. Menigioma bulk RNA-seq, scRNA-seq, and spatial datasets are incorporated, but it remains unclear how variability across datasets, platforms, and tumor grades was handled. Clarifying harmonization strategies and limitations would help readers assess robustness of the developmental–tumor mapping."

"The conclusion that “Over time, endothelial cells transitioned from the primary vascular plexus at PCW 5, to arterial and venous capillaries around PCW 7-9, and larger arterioles and venous lymphatic-like cells at PCW 12-13” is not adequately supported by the current analysis. Trajectory or pseudotime analysis would be more appropriate for demonstrating developmental transitions. Please consider pseudotime analysis. Additionally, Fig. 3E appears to show cell composition rather than transitional changes."

"The observation that the forebrain lacks venous capillaries at PCW13 may be due to limited sample size. This finding should be presented cautiously unless validated in a larger cohort."

"Lines 403-405: Please specify in the Methods how the signature scores were computed and provide the list of the 50 enriched genes used to build these signatures. It is also unclear how these enriched genes were determined, please clarify that."

"Transferring scRNA-seq LDA topics directly onto the meningioma spatial transcriptomic data raises methodological concerns. LDA topics are inherently dataset-specific, reflecting the latent gene program structure of the dataset on which they were trained. Given the substantial biological and technical differences between the scRNA-seq data and the spatial data, direct topic transfer may not faithfully capture the latent structure of the tumor samples. A more robust analysis would be to perform LDA independently on the spatial

transcriptomics samples, characterize the tumor-specific topics, and then evaluate their correspondence to the scRNA-seq programs."

Reviewer #4:
"It would be helpful if the authors could briefly comment on how batch effects were assessed and addressed. Because all cells were integrated into a single dataset for top-level cytograph clustering, a quantitative summary of donor and developmental-stage composition (e.g., per-cluster donor/stage fractions or per-sample cell-type compositions) would strengthen the manuscript. Although the UMAPs do not show obvious batch-driven artifacts, providing such summaries would increase confidence that major clusters are robust and shared across donors and developmental stages, further enhancing the utility of the atlas as a resource."

"For the Xenium datasets, the decision to retain only transcripts within segmented nuclei seems reasonable to minimize segmentation artifacts, but it may discard a substantial fraction of cytoplasmic signal. A short comment on how this choice might influence sensitivity for particular cell types, or whether any systematic bias across lineages was observed, would be informative for interpreting the spatial data."

"RNA diffusion or “smearing” is a known and common issue in spatial transcriptomics and can lead to apparent expression in neighboring cells. Is it possible that CDH1 expression in “inner dura” cells reflects diffusion from adjacent arachnoid barrier cells rather than authentic endogenous expression? A short discussion or control analysis would help clarify the observed signal is intrinsic or an artifact."

(B) Substantially revise per our instructions on the comment by Reviewers #2 and #3: "The inference that meningioma originates from dura fibroblast states, although compelling, requires caution and further validation. The developmental similarity between dura fibroblasts and meningioma transcriptomes is intriguing, but the claim of a dura-origin model would benefit from more explicit mechanistic evidence. Functional validation—genetic, lineage tracing, or perturbation studies—would be needed to solidify this paradigm shift". In terms of this comment, please note the following:
* While we find the comment about mechanistic dissection of interest, we consider it to be beyond the scope of the current study, which is being considered as a Resource; so we would not require new experiments and analyses aiming to mechanistically dissect this part.
* However, we would require the origin claim to be validated and fully supported by data if it is to be made. We have discussed the issue within the editorial team and we would welcome data per the reviewers' instructions to validate this claim. That said, we have decided that the manuscript presents sufficient interest for us as a Resource regardless of this claim, which means that we would be happy if you prefer to textually revise your claims, instead of performing new experiments for this specific comment; in this case, however, we would ask that you please ensure you have substantially revised the text and carefully ensured you have made no strong claims about meningioma origin throughout the manuscript before resubmission.

(C) Address the issues raised by reviewer #4 regarding the part of the study referring to meningioma data:

"In Figure 6, panel F provides a helpful visual summary of mean topic scores across tumors, showing a clear separation between arachnoid/pia vs. inner-dura/dura/barrier. However, the evidence remains largely descriptive. Given that this topic analysis underpins a central claim regarding the putative cell of origin, adding a statistical comparison at the individual sample level would strengthen the claim (for example, per-tumor mean topic weights for arachnoid versus inner dura/barrier topics, with effect sizes and confidence intervals)."

"Previous studies have commonly suggested the arachnoid as the putative cell of origin for meningioma, whereas the current study identified dura-related populations as dominant among tumor cells. It would be helpful if the authors could briefly summarize the historical rationale behind the arachnoid-origin hypothesis and clarify how their findings reshape this understanding."

"Some of the 20 most frequently mutated genes such as NF1 and NF2 are tumor suppressors, and their transcript levels are often depleted in meningiomas, especially in NF-loss tumors. Therefore, it is somewhat surprising to see them included in this analysis and highly expressed in iDura populations. Instead of focusing on these tumor suppressors, it may be more informative to highlight proto-oncogenes whose elevated expression is commonly observed in tumor cells and may better reflect lineage- or state-specific oncogenic programs."

(D) All other referee concerns pertaining to strengthening existing data, providing clarifications and improving presentation, should also be addressed.

(E) Finally, please pay close attention to our guidelines on statistical and methodological reporting (listed below) as failure to do so may delay the reconsideration of the revised manuscript. In particular please provide:

- unprocessed images of all gels/blots in the form of a multi-page pdf file. Please ensure that blots/gels are labeled and the sections presented in the figures are clearly indicated.

- all numerical source data in Excel format, with data for different figures provided as different sheets within a single Excel file. The file should include source data giving rise to graphical representations and statistical descriptions in the paper and for all instances where the figures present representative experiments of multiple independent repeats, the source data of all repeats should be provided.

We would be happy to consider a revised manuscript that would satisfactorily address these points.

When revising the manuscript please:

- ensure that it conforms to our format instructions and publication policies (see below and www.nature.com/nature/authors/).

- provide a point-by-point rebuttal to the full referee reports verbatim, as provided at the end of this letter.

- provide the completed Reporting Summary (found here https://www.nature.com/authors/policies/ReportingSummary.pdf). This is essential for reconsideration of the manuscript and this document will be available to editors and referees in the event of peer review. For more information see http://www.nature.com/authors/policies/availability.html or contact me.

Nature Cell Biology is committed to improving transparency in authorship. As part of our efforts in this direction, we are now requesting that all authors identified as ‘corresponding author’ on published papers create and link their Open Researcher and Contributor Identifier (ORCID) with their account on the Manuscript Tracking System (MTS), prior to acceptance. ORCID helps the scientific community achieve unambiguous attribution of all scholarly contributions. You can create and link your ORCID from the home page of the MTS by clicking on ‘Modify my Springer Nature account’. For more information please visit please visit www.springernature.com/orcid.

Please submit the revised manuscript files and the point-by-point rebuttal to the referee comments using this link:

Link Redacted

*This url links to your confidential home page and associated information about manuscripts you may have submitted or be reviewing for us. If you wish to forward this email to co-authors, please delete the link to your homepage.

We would like to receive a revised submission within six months. We would be happy to consider a revision even after this timeframe, however if the resubmission deadline is missed and the paper is eventually published, the submission date will be the date when the revised manuscript was received.

We hope that you will find our referees' comments, and editorial guidance helpful. Please do not hesitate to contact me if there is anything you would like to discuss.

Best wishes,

Stelios

Dr. Stylianos Lefkopoulos
He/him/his
Senior Editor, Nature Cell Biology
Springer Nature
Heidelberger Platz 3, 14197 Berlin, Germany

E-mail: stylianos.lefkopoulos@springernature.com
Bluesky: @slefkopoulos.bsky.social
LinkedIn: www.linkedin.com/in/stylianos-lefkopoulos-81b007a0

Reviewers' Comments:

Reviewer #1 (Remarks to the Author):

This study presents a comprehensive atlas of human developing meninges using snRNAseq integrated with extensive spatial transcriptomics data from 13 donors, 16 samples spanning 5-13 post-conception weeks (pcw). The sampling includes primarily forebrain samples with regional dissections at 10 pcw, supplemented by regional spatial transcriptomics (Xenium, 389 genes) at 6 and 9.5 pcw.
Key findings include:
• Comprehensive characterization of immune, vascular, and fibroblast populations during meningeal development from primary meninx through later maturation stages
• Discovery of an inner CDH-1+ dura lineage
• Identification of ELP progenitors of B-cell lineages in the primary meninx at 5 pcw, placing them in the periphery one week earlier than previously found in fetal liver
• Demonstration of early vascular specification and subarachnoid lymphangiogenesis in fetal development

• Characterization of choroid plexus development showing early epithelial cell specification and function similar to rodents, with a potentially human-specific fibroblast type expressing TCF21 and immune-related genes (PTPRC, CD38)
• Evidence that meningioma tumors likely arise from the inner dura lineage rather than arachnoid lineages, based on spatial analyses of 10 meningioma tumors with published scRNAseq and bulk RNAseq datasets, new spatial transcriptomics of adult dura samples, gene expression similarities, and enrichment of driver gene expression in dural lineages

This study addresses a critical knowledge gap in the field by providing the first comprehensive atlas of human developing meninges at this scale and resolution. The datasets represent an important resource for the developmental neuroscience community.

The early identification of B-cell lineage progenitors at 5 pcw is an important developmental finding showing earlier distribution than liver one week later.

The discovery of the inner CDH-1+ dura lineage is highly novel and represents one of the most important findings of the paper. The reinterpretation of meningioma cellular origins—challenging the prior hypothesis of arachnoid derivation in favor of dural derivation—is significant and well-supported by multiple lines of evidence. This hypothesis is particularly well supported through: (1) spatial transcriptomics demonstrating the existence and organization of the inner dural layer, (2) gene expression similarities between tumors and inner dura, (3) enrichment of driver genes in developing dura cells, and (4) integration with existing published datasets.

The experimental design is excellent, with appropriate sampling across developmental timepoints and integration of multiple complementary technologies (snRNAseq, spatial transcriptomics, immunohistochemistry on floating sections). The data quality is exceptional. Integration of datasets shows minimal batch correction, indicating technically sound experimental execution. The combination of snRNAseq with spatial transcriptomics (Xenium) provides robust validation of cell type identities and spatial relationships.

The paper reads well overall. However, minor presentation issues exist:
• Figure 4d insets are too small to visualize clearly and should be enlarged
• Figure S6B UMAP lacks a color key
• Sex of samples is not identified in Figure S1

The addition of validation using immunohistochemistry on floating sections provides an excellent complementary layer of validation beyond the spatial transcriptomics.

Topic modeling was used to excellent effect to compare scRNAseq data with spatial data, demonstrating enrichment of dural versus arachnoid signatures in meningioma.

Overall Assessment
I find few issues with this paper. The analyses are comprehensive, appropriate, and well-presented. The resource is important, and new findings are impactful. The study is exceptionally well-written and accessible to a broad audience. The paper effectively communicates both the technical aspects of the multi-modal approach and the biological significance of the findings. This study represents an exceptional contribution to the field of meningeal development and provides critical insights into meningioma biology.

Reviewer #2 (Remarks to the Author):

The authors aimed to generate a comprehensive cellular and spatial atlas of human meningeal development from 5 to 13 post-conception weeks (PCW) using high-resolution scRNA-seq and spatial transcriptomics, and connect these developmental trajectories with adult meningeal biology and meningioma tumorigenesis. They studied the emergence of pia, arachnoid, and dura fibroblast populations, the maturation of immune and vascular compartments, and linked fetal meningeal cell states to meningioma molecular signatures. Notably, the authors challenge the long-standing paradigm of arachnoid cap cell origin for meningioma and propose a new model in which tumors arise from dura-derived fibroblast states. This work provides a valuable foundation for understanding meningeal development and disease.

The work contained several strengths, including the integration of developmental and tumor datasets, the use of multi-platform data (single-cell and spatial), and the discovery of an inner dura layer with barrier-like features. The proposed dura-origin model for meningioma offers an intriguing and potentially field-shifting perspective. The dataset is extensive with >150,000 fetal cells providing a useful reference for future developmental and disease research.

There are, however, several directions to improve:

1. Sample metadata and donor characteristics require further clarification. This study includes samples from 13 donors across 5–13 PCW, but additional details on donor demographics, biological variance, and potential confounders (e.g., gestational age precision, maternal health, tissue procurement factors) would enhance interpretability. As this atlas will serve as a foundational resource, transparent metadata reporting is essential for future reanalysis and integration.
2. Quality control, batch correction, and computational pipeline details should be more fully described. Given the multi-sample, multi-platform nature of the dataset, clear documentation of QC thresholds, filtering criteria, and how batch effects were mitigated is critical for reproducibility. Furthermore, it would be beneficial to compare the newly generated fetal data with existing adult or murine meningeal datasets to assess biological consistency. Even though no prior study integrates fetal and adult meninges comprehensively, benchmarking against publicly available single-platform datasets would strengthen confidence in the conclusions.
3 The inference that meningioma originates from dura fibroblast states, although compelling, requires caution and further validation. The developmental similarity between dura fibroblasts and meningioma transcriptomes is intriguing, but the claim of a dura-origin model would benefit from more explicit mechanistic evidence. Functional validation—genetic, lineage tracing, or perturbation studies—would be needed to solidify this paradigm shift. Additionally, tumor–fetal comparisons are sensitive to batch variability and tumor heterogeneity, and further discussion of these limitations is warranted.

4 Spatial validation of fibroblast and barrier-like layers could be expanded. The discovery of an inner dura layer expressing barrier genes is interesting, but appears to rely heavily on selected spatial slices. A more systematic description of spatial reproducibility across donors, sections, and developmental stages would increase confidence in this structural finding, especially since it affects the proposed disease-origin model.
5 Integration of tumor samples would benefit from more detailed explanation. Menigioma bulk RNA-seq, scRNA-seq, and spatial datasets are incorporated, but it remains unclear how variability across datasets, platforms, and tumor grades was handled. Clarifying harmonization strategies and limitations would help readers assess robustness of the developmental–tumor mapping.

Below are the comments from an early career researcher co-reviewed this paper:

The study addresses an important biological question, but several methodological issues require further clarification or additional analyses to support the authors conclusions.

- The conclusion that "Over time, endothelial cells transitioned from the primary vascular plexus at PCW 5, to arterial and venous capillaries around PCW 7-9, and larger arterioles and venous lymphatic-like cells at PCW 12-13" is not adequately supported by the current analysis. Trajectory or pseudotime analysis would be more appropriate for demonstrating developmental transitions. Please consider pseudotime analysis. Additionally, Fig. 3E appears to show cell composition rather than transitional changes.
- The observation that the forebrain lacks venous capillaries at PCW13 may be due to limited sample size. This finding should be presented cautiously unless validated in a larger cohort.
- Lines 403-405: Please specify in the Methods how the signature scores were computed and provide the list of the 50 enriched genes used to build these signatures. It is also unclear how these enriched genes were determined, please clarify that.
- Given the distinct biological differences between outer dura, inner dura, the meningeal barrier, and the skull/dura progenitor population as described in the section "Identification of an inner and outer dura layer", a cell-cell communication analysis would be highly informative. A ligand-receptor analysis would identify the signaling pathways that drive differentiation of outer versus inner dura and pattern formation. Such analysis would offer mechanistic insight into how these layers interact and are specified during PCW5-6.
- Lines 436-439: The multi-layer fibroblasts organizations (meningeal barrier, inner dura sheet, periosteal layer, periderm) suggests the presence of distinct fibroblasts niches. A spatial niche detection or neighborhood analysis would substantially strengthen the interpretation of fibroblast layer formation.
- Lines 532-538, The LDA analysis identifies 35 gene modules (topics), and several are described as corresponding to pia, arachnoid, PTGDS+ precursors, inner dura, and dura. For interpretability it would be very helpful to report the top genes contributing to each topic (e.g., top 10–20 genes per module) or atleast the topics reported in Fig. 6A and to provide annotation linking these top genes to cell types or anatomical locations where these modules show higher scores. This would allow readers to better understand which transcriptional programs each topic represents, and what genes are the top contributors to these topics.
- Transferring scRNA-seq LDA topics directly onto the meningioma spatial transcriptomic data raises methodological concerns. LDA topics are inherently dataset-specific, reflecting the latent gene program structure of the dataset on which they were trained. Given the substantial biological and technical differences between the scRNA-seq data and the spatial data, direct topic transfer may not faithfully capture the latent structure of the tumor samples. A more robust analysis would be to perform LDA independently on the spatial transcriptomics samples, characterize the tumor-specific topics, and then evaluate their correspondence to the scRNA-seq programs.
- Lines 582-592: The description of spatially structured vs uniformly mixed tumors, as well as the presence of nested dura-like, inner-dura-like, periosteal-like, chondrogenic, and cranial-like regions (Fig. 6H), strongly suggests the existence of distinct cellular neighborhoods or niches. It would be highly informative to perform a cellular neighborhood or niche detection analysis to provide a better understanding of the spatial organization of meningiomas, vascular-immune microenvironments, dura-like vs cranial-like spatial architectures and would support many of the spatial claims made in the manuscript.
- The Methods section does not explain why K=35 topics were selected for LDA. Please justify the choice of topic number.
- Some LDA topics were described as not biologically meaningful. Did the authors consider using a smaller K (topic number) to obtain fewer but more coherent and interpretable topics?

Reviewer #3 (Remarks to the Author):

I co-reviewed this manuscript with one of the reviewers who provided the listed reports. This is part of the Nature Cell Biology initiative to facilitate training in peer review and to provide appropriate recognition for Early Career Researchers who co-review manuscripts.

Reviewer #4 (Remarks to the Author):

The authors present an impressive and well-curated dataset of human meninges across development, adult dura, and meningiomas, profiled using both scRNA-seq and spatial transcriptomics. Major cellular compartments are clearly clustered and annotated, and the developmental time series is leveraged effectively to highlight changes in fibroblast, vascular, and immune populations over time. Overall, this represents a valuable resource, and the authors should be commended for putting together such a comprehensive dataset.

Major comments
1. It would be helpful if the authors could briefly comment on how batch effects were assessed and addressed. Because all cells were integrated into a single dataset for top-level cytograph clustering, a quantitative summary of donor and developmental-stage composition (e.g., per-cluster donor/stage fractions or per-sample cell-type compositions) would strengthen the manuscript. Although the UMAPs do not show obvious batch-driven artifacts, providing such summaries would increase confidence that major clusters are robust and shared across donors and developmental stages, further enhancing the utility of the atlas as a resource.

2. In Figure 6, panel F provides a helpful visual summary of mean topic scores across tumors, showing a clear separation between arachnoid/pia vs. inner-dura/dura/barrier. However, the evidence remains largely descriptive. Given that this topic analysis underpins a central claim regarding the putative cell of origin, adding a statistical comparison at the individual sample level would strengthen the claim (for example, per-tumor mean topic weights for arachnoid versus inner dura/barrier topics, with effect sizes and confidence intervals).

3. For the Xenium datasets, the decision to retain only transcripts within segmented nuclei seems reasonable to minimize segmentation artifacts, but it may discard a substantial fraction of cytoplasmic signal. A short comment on how this choice might influence sensitivity for

particular cell types, or whether any systematic bias across lineages was observed, would be informative for interpreting the spatial data.

4. As the meninges mature as a continuum of cell states, it would be informative to apply trajectory analysis, such as pseudotime and RNA velocity, to identify transcriptional factors or master regulators driving the transitions between developmental or lineage-specific states.

5. Previous studies have commonly suggested the arachnoid as the putative cell of origin for meningioma, whereas the current study identified dura-related populations as dominant among tumor cells. It would be helpful if the authors could briefly summarize the historical rationale behind the arachnoid-origin hypothesis and clarify how their findings reshape this understanding.

6. RNA diffusion or “smearing” is a known and common issue in spatial transcriptomics and can lead to apparent expression in neighboring cells. Is it possible that CDH1 expression in “inner dura” cells reflects diffusion from adjacent arachnoid barrier cells rather than authentic endogenous expression? A short discussion or control analysis would help clarify the observed signal is intrinsic or an artifact.

7. Some of the 20 most frequently mutated genes such as NF1 and NF2 are tumor suppressors, and their transcript levels are often depleted in meningiomas, especially in NF-loss tumors. Therefore, it is somewhat surprising to see them included in this analysis and highly expressed in iDura populations. Instead of focusing on these tumor suppressors, it may be more informative to highlight proto-oncogenes whose elevated expression is commonly observed in tumor cells and may better reflect lineage- or state-specific oncogenic programs.

GUIDELINES FOR MANUSCRIPT SUBMISSION TO NATURE CELL BIOLOGY

READABILITY OF MANUSCRIPTS – Nature Cell Biology is read by cell biologists from diverse backgrounds, many of whom are not native English speakers. Authors should aim to communicate their findings clearly, explaining technical jargon that might be unfamiliar to non-specialists, and avoiding non-standard abbreviations. Titles and abstracts should concisely communicate the main findings of the study, and the background, rationale, results and conclusions should be clearly explained in the manuscript in a manner accessible to a broad cell biology audience. Nature Cell Biology uses British spelling.

MANUSCRIPT FORMAT – please follow the guidelines listed in our Guide to Authors regarding manuscript formats at Nature Cell Biology.

TITLE – should be no more than 100 characters including spaces, without punctuation and avoiding technical terms, abbreviations, and active verbs..

AUTHOR NAMES – should be given in full.

AUTHOR AFFILIATIONS – should be denoted with numerical superscripts (not symbols) preceding the names. Full addresses should be included, with US states in full and providing zip/post codes. The corresponding author is denoted by: "Correspondence should be addressed to [initials]."

ABSTRACT AND MAIN TEXT – please follow the guidelines that are specific to the format of your manuscript, as listed in our Guide to Authors (http://www.nature.com/ncb/pdf/ncb_gta.pdf) Briefly, Nature Cell Biology Articles, Resources and Technical Reports have 3500 words, including a 150 word abstract, and the main text is subdivided in Introduction, Results, and Discussion sections. Nature Cell Biology Letters have up to 2500 words, including a 180 word introductory paragraph (abstract), and the text is not subdivided in sections.

ACKNOWLEDGEMENTS – should be kept brief. Professional titles and affiliations are unnecessary. Grant numbers can be listed.

AUTHOR CONTRIBUTIONS – must be included after the Acknowledgements, detailing the contributions of each author to the paper (e.g. experimental work, project planning, data analysis etc.). Each author should be listed by his/her initials.

FINANCIAL AND NON-FINANCIAL COMPETING INTERESTS – the authors must include one of three declarations: (1) that they have no financial and non-financial competing interests; (2) that they have financial and non-financial competing interests; or (3) that they decline to respond, after the Author Contributions section. This statement will be published with the article, and in cases where financial and non-financial competing interests are declared, these will be itemized in a web supplement to the article. For further details please see https://www.nature.com/licenceforms/nrg/competing-interests.pdf.

REFERENCES – are limited to a total of 70 for Articles, Resources, Technical Reports; and 40 for Letters. This includes references in the main text and Methods combined. References must be numbered sequentially as they appear in the main text, tables and figure legends and Methods and must follow the precise style of Nature Cell Biology references. References only cited in the Methods should be numbered consecutively following the last reference cited in the main text. References only associated with Supplementary Information (e.g. in supplementary legends) do not count toward the total reference limit and do not need to be cited in numerical continuity with references in the main text. Only published papers can be cited, and each publication cited should be included in the numbered reference list, which should include the manuscript titles. Footnotes are not permitted.

METHODS – Nature Cell Biology publishes methods online. The methods section should be provided as a separate Word document, which will be copyedited and appended to the manuscript PDF, and incorporated within the HTML format of the paper.

Methods should be written concisely, but should contain all elements necessary to allow interpretation and replication of the results. As a guideline, Methods sections typically do not exceed 3,000 words. The Methods should be divided into subsections listing reagents and techniques. When citing previous methods, accurate references should be provided and any alterations should be noted. Information must be provided about: antibody dilutions, company names, catalogue numbers and clone numbers for monoclonal antibodies; sequences of RNAi and cDNA probes/primers or company names and catalogue numbers if reagents are commercial; cell line names, sources and information on cell line identity and authentication. Animal studies and experiments involving human subjects must be reported in detail, identifying the committees approving the protocols. For studies involving human subjects/samples, a statement must be included confirming that informed consent was obtained. Statistical analyses and information on the reproducibility of experimental results should be provided in a section titled "Statistics and Reproducibility".

All Nature Cell Biology manuscripts submitted on or after March 21 2016 must include a Data availability statement at the end of the Methods section. For Springer Nature policies on data availability see http://www.nature.com/authors/policies/availability.html; for more information on this particular policy see http://www.nature.com/authors/policies/data/data-availability-statements-data-citations.pdf. The Data availability statement should include:

• Accession codes for primary datasets (generated during the study under consideration and designated as "primary accessions") and secondary datasets (published datasets reanalysed during the study under consideration, designated as "referenced accessions"). For primary accessions data should be made public to coincide with publication of the manuscript. A list of data types for which submission to community-endorsed public repositories is mandated (including sequence, structure, microarray, deep sequencing data) can be found here http://www.nature.com/authors/policies/availability.html#data.

• Unique identifiers (accession codes, DOIs or other unique persistent identifier) and hyperlinks for datasets deposited in an approved repository, but for which data deposition is not mandated (see here for details http://www.nature.com/sdata/data-policies/repositories).

• At a minimum, please include a statement confirming that all relevant data are available from the authors, and/or are included with the manuscript (e.g. as source data or supplementary information), listing which data are included (e.g. by figure panels and data types) and mentioning any restrictions on availability.

• If a dataset has a Digital Object Identifier (DOI) as its unique identifier, we strongly encourage including this in the Reference list and citing the dataset in the Methods.

We recommend that you upload the step-by-step protocols used in this manuscript to protocols.io. More details can found at https://www.protocols.io/help/publish-articles.

DISPLAY ITEMS – main display items are limited to 6-8 main figures and/or main tables for Articles, Resources, Technical Reports; and 5 main figures and/or main tables for Letters. For Supplementary Information see below.

FIGURES – Colour figure publication costs $600 for the first, and $300 for each subsequent colour figure. All panels of a multi-panel figure must be logically connected and arranged as they would appear in the final version. Unnecessary figures and figure panels should be avoided (e.g. data presented in small tables could be stated briefly in the text instead).

All imaging data should be accompanied by scale bars, which should be defined in the legend.
Cropped images of gels/blots are acceptable, but need to be accompanied by size markers, and to retain visible background signal within the linear range (i.e. should not be saturated). The boundaries of panels with low background have to be demarked with black lines. Splicing of panels should only be considered if unavoidable, and must be clearly marked on the figure, and noted in the legend with a statement on whether the samples were obtained and processed simultaneously. Quantitative comparisons between samples on different gels/blots are discouraged; if this is unavoidable, it should only be performed for samples derived from the same experiment with gels/blots were processed in parallel, which needs to be stated in the legend.

Figures should be provided at approximately the size that they are to be printed at (single column is 86 mm, double column is 170 mm) and should not exceed an A4 page (8.5 x 11"). Reduction to the scale that will be used on the page is not necessary, but multi-panel figures should be sized so that the whole figure can be reduced by the same amount at the smallest size at which essential details in each panel are visible. In the interest of our colour-blind readers we ask that you avoid using red and green for contrast in figures. Replacing red with magenta and green with turquoise are two possible colour-safe alternatives. Lines with widths of less than 1 point should be avoided. Sans serif typefaces, such as Helvetica (preferred) or Arial should be used. All text that forms part of a figure should be rewritable and removable.

We accept files from the following graphics packages in either PC or Macintosh format:

- For line art, graphs, charts and schematics we prefer Adobe Illustrator (.AI), Encapsulated PostScript (.EPS) or Portable Document Format (.PDF). Files should be saved or exported as such directly from the application in which they were made, to allow us to restyle them according to our journal house style.

- We accept PowerPoint (.PPT) files if they are fully editable. However, please refrain from adding PowerPoint graphical effects to objects, as this results in them outputting poor quality raster art. Text used for PowerPoint figures should be Helvetica (preferred) or Arial.

- We do not recommend using Adobe Photoshop for designing figures, but we can accept Photoshop generated (.PSD or .TIFF) files only if each element included in the figure (text, labels, pictures, graphs, arrows and scale bars) are on separate layers. All text should be editable in 'type layers' and line-art such as graphs and other simple schematics should be preserved and embedded within 'vector smart objects' - not flattened raster/bitmap graphics.

- Some programs can generate Postscript by 'printing to file' (found in the Print dialogue). If using an application not listed above, save the file in PostScript format or email our Art Editor, Allen Beattie for advice (a.beattie@nature.com).

Regardless of format, all figures must be vector graphic compatible files, not supplied in a flattened raster/bitmap graphics format, but should be fully editable, allowing us to highlight/copy/paste all text and move individual parts of the figures (i.e. arrows, lines, x and y axes, graphs, tick marks, scale bars etc.). The only parts of the figure that should be in pixel raster/bitmap format are photographic images or 3D rendered graphics/complex technical illustrations.

All placed images (i.e. a photo incorporated into a figure) should be on a separate layer and independent from any superimposed scale bars or text. Individual photographic images must be a minimum of 300+ DPI (at actual size) or kept constant from the original picture acquisition and not decreased in resolution post image acquisition. All colour artwork should be RGB format.

FIGURE LEGENDS – must not exceed 350 words for each figure to allow fit on a single printed NCB page together with the figure. They must include a brief title for the whole figure, and short descriptions of each panel with definitions of the symbols used, but without detailing methodology.

TABLES – main tables should be provided as individual Word files, together with a brief title and legend. For supplementary tables see below.

SUPPLEMENTARY INFORMATION – Supplementary information is material directly relevant to the conclusion of a paper, but which cannot be included in the printed version in order to keep the manuscript concise and accessible to the general reader. Supplementary information is an integral part of a Nature Cell Biology publication and should be prepared and presented with as much care as the main display item, but it must not include non-essential data or text, which may be removed at the editor's discretion. All supplementary material is fully peer-reviewed and published online as part of the HTML version of the manuscript. Supplementary Figures and Supplementary Notes are appended at the end of the main PDF of the published manuscript.

Supplementary items should relate to a main text figure, wherever possible, and should be mentioned sequentially in the main manuscript, designated as Supplementary Figure, Table, Video, or Note, and numbered continuously (e.g. Supplementary Figure 1, Supplementary Figure 2, Supplementary Table 1, Supplementary Table 2 etc.).

Unprocessed scans of all key data generated through electrophoretic separation techniques need to be presented in a supplementary figure that should be labelled and numbered as the final supplementary figure, and should be mentioned in every relevant figure legend. This figure does not count towards the total number of figures and is the only figure that can be displayed over multiple pages, but should be provided as a single file, in PDF or TIFF format. Data in this figure can be displayed in a relatively informal style, but size markers and the figures panels corresponding to the presented data must be indicated.

The total number of Supplementary Figures (not including the "unprocessed scans" Supplementary Figure) should not exceed the number of main display items (figures and/or tables (see our Guide to Authors and March 2012 editorial http://www.nature.com/ncb/authors/submit/index.html#suppinfo; http://www.nature.com/ncb/journal/v14/n3/index.html#ed). No restrictions apply to Supplementary Tables or Videos, but we advise authors to be selective in including supplemental data.

Each Supplementary Figure should be provided as a single page and as an individual file in one of our accepted figure formats and should be presented according to our figure guidelines (see above). Supplementary Tables should be provided as individual Excel files. Supplementary Videos should be provided as .avi or .mov files up to 50 MB in size. Supplementary Figures, Tables and Videos much be accompanied by a separate Word document including titles and legends.

GUIDELINES FOR EXPERIMENTAL AND STATISTICAL REPORTING

REPORTING REQUIREMENTS – To improve the quality of methods and statistics reporting in our papers we have recently revised the reporting checklist we introduced in 2013. We are now asking all life sciences authors to complete two items: an Editorial Policy Checklist (found here https://www.nature.com/authors/policies/Policy.pdf) that verifies compliance with all required editorial policies and a reporting summary (found here https://www.nature.com/authors/policies/ReportingSummary.pdf) that collects information on experimental design and reagents. These documents are available to referees to aid the evaluation of the manuscript. Please note that these forms are dynamic 'smart pdfs' and must therefore be downloaded and completed in Adobe Reader. We will then flatten them for ease of use by the reviewers. If you would like to reference the guidance text as you complete the template, please access these flattened versions at http://www.nature.com/authors/policies/availability.html.

STATISTICS – Wherever statistics have been derived the legend needs to provide the n number (i.e. the sample size used to derive statistics) as a precise value (not a range), and define what this value represents. Error bars need to be defined in the legends (e.g. SD, SEM) together with a measure of centre (e.g. mean, median). Box plots need to be defined in terms of minima, maxima, centre, and percentiles. Ranges are more appropriate than standard errors for small data sets. Wherever statistical significance has been derived, precise p values need to be provided and the statistical test used needs to be stated in the legend. Statistics such as error bars must not be derived from $n<3$. For sample sizes of $n<5$ please plot the individual data points rather than providing bar graphs. Deriving statistics from technical replicate samples, rather than biological replicates is strongly discouraged. Wherever statistical significance has been derived, precise p values need to be provided and the statistical test stated in the legend.

Information on how many times each experiment was repeated independently with similar results needs to be provided in the legends and/or Methods for all experiments, and in particular wherever representative experiments are shown.

We strongly recommend the presentation of source data for graphical and statistical analyses as a separate Supplementary Table, and request that source data for all independent repeats are provided when representative experiments of multiple independent repeats, or

averages of two independent experiments are presented. This supplementary table should be in Excel format, with data for different figures provided as different sheets within a single Excel file. It should be labelled and numbered as one of the supplementary tables, titled “Statistics Source Data”, and mentioned in all relevant figure legends.

--------- Please don't hesitate to contact NCB@nature.com should you have queries about any of the above requirements ---------

Version 1:

Decision Letter:

10th May 2026

Dear Sten,

Thank you for submitting your revised manuscript "Cell atlas of the developing human meninges reveals a dura-like nature of meningiomas" (NCB-RS60100A). It has now been seen by the original referees and their comments are below. The reviewers find that the paper has improved in revision, and therefore we'll be happy in principle to publish it in Nature Cell Biology, pending minor revisions to comply with our editorial and formatting guidelines.

If the current version of your manuscript is in a PDF format, please email us a copy of the file in an editable format (Microsoft Word or LaTex)-- we cannot proceed with PDFs at this stage.

We are now performing detailed checks on your paper and will send you a checklist detailing our editorial and formatting requirements in about 1 to 2 weeks. Please do not upload the final materials and make any revisions until you receive this additional information from us.

Thank you again for your interest in Nature Cell Biology. Please do not hesitate to contact me if you have any questions.

Best wishes,
Stelios

Dr. Stylianos Lefkopoulos
He/him/his
Senior Editor, Nature Cell Biology
Springer Nature
Heidelberger Platz 3, 14197 Berlin, Germany

E-mail: stylianos.lefkopoulos@springernature.com
Bluesky: @slefkopoulos.bsky.social
LinkedIn: www.linkedin.com/in/stylianos-lefkopoulos-81b007a0

Reviewer #1 (Remarks to the Author):

The authors have appropriately addressed the minor issues I flagged. Further, they have nicely addressed the comments of the other reviewers. I reiterate that this is an excellent and important resource.

Reviewer #2 (Remarks to the Author):

Authors have addressed all concerns

Reviewer #3 (Remarks to the Author):

I co-reviewed this manuscript with one of the reviewers who provided the listed reports. This is part of the Nature Cell Biology initiative to facilitate training in peer review and to provide appropriate recognition for Early Career Researchers who co-review manuscripts.

Reviewer #4 (Remarks to the Author):

The authors have provided satisfactory responses to all concerns raised, and the manuscript has been strengthened as a result. Overall, I believe the dataset represents a valuable resource for the community, and the authors are to be congratulated for the considerable effort put into this work.

Version 2:

Decision Letter:

Dear Sten,

I am pleased to inform you that your manuscript, "Cell atlas of the developing human meninges reveals a dura-like nature of meningiomas", has now been accepted for publication in Nature Cell Biology. Congratulations!

Thank you for sending us the final manuscript files to be processed for print and online production, and for returning the manuscript checklists and other forms. Your manuscript will now be passed to our production team who will be in contact with you if there are any questions with the production quality of supplied figures and text.

Over the next few weeks, your paper will be copyedited to ensure that it conforms to Nature Cell Biology style. Once your paper is typeset, you will receive an email with a link to choose the appropriate publishing options for your paper and our Author Services team will be in touch regarding any additional information that may be required.

After the grant of rights is completed, you will receive a link to your electronic proof via email with a request to make any corrections within 48 hours. If, when you receive your proof, you cannot meet this deadline, please inform us at rjsproduction@springernature.com immediately.

You will not receive your proofs until the publishing agreement has been received through our system.

Due to the importance of these deadlines, we ask that you please let us know now whether you will be difficult to contact over the next month. If this is the case, we ask you provide us with the contact information (email, phone and fax) of someone who will be able to check the proofs on your behalf, and who will be available to address any last-minute problems.

If you have any questions about our publishing options, costs, Open Access requirements, or our legal forms, please contact ASJournals@springernature.com

Once your paper has been scheduled for online publication, the Nature press office will be in touch to confirm the details. An online order form for reprints of your paper is available at https://www.nature.com/reprints/author-reprints.html. All co-authors, authors' institutions and authors' funding agencies can order reprints using the form appropriate to their geographical region.

Publication is conditional on the manuscript not being published elsewhere and on there being no announcement of this work to any media outlet until the online publication date in Nature Cell Biology.

You may wish to make your media relations office aware of your accepted publication, in case they consider it appropriate to organize some internal or external publicity. Once your paper has been scheduled you will receive an email confirming the publication details. This is normally 3-4 working days in advance of publication. If you need additional notice of the date and time of publication, please let the production team know when you receive the proof of your article to ensure there is sufficient time to coordinate. Further information on our embargo policies can be found here: https://www.nature.com/authors/policies/embargo.html

Please note that *Nature Cell Biology* is a Transformative Journal (TJ). Authors may publish their research with us through the traditional subscription access route or make their paper immediately open access through payment of an article-processing charge (APC). Authors will not be required to make a final decision about access to their article until it has been accepted. Find out more about Transformative Journals

Authors may need to take specific actions to achieve compliance with funder and institutional open access mandates. If your research is supported by a funder that requires immediate open access (e.g. according to Plan S principles or the NIH public access policy) then you should select the gold OA route, and we will direct you to the compliant route where possible. Because authors warrant under our subscription licensing terms that they haven't committed to licensing any version of their article under a licence inconsistent with the terms of our agreement – including the applicable embargo period – publication under the subscription model isn't suitable for authors whose funders require no embargo.

To assist our authors in disseminating their research to the broader community, our SharedIt initiative provides you with a unique shareable link that will allow anyone (with or without a subscription) to read the published article. Recipients of the link with a subscription will also be able to download and print the PDF.

If your paper includes color figures, please be aware that in order to help cover some of the additional cost of four-color reproduction, Nature Portfolio charges our authors a fee for the printing of their color figures. Please contact our offices for exact pricing and details.

As soon as your article is published, you will receive an automated email with your shareable link.

If you have not already done so, we strongly recommend that you upload the step-by-step protocols used in this manuscript to protocols.io (https://protocols.io), an open online resource that allows researchers to share their detailed experimental know-how. All uploaded protocols are made freely available and are assigned DOIs for ease of citation. Protocols and Nature Portfolio journal papers in which they are used can be linked to one another, and this link is clearly and prominently visible in the online versions of both. Authors who performed the specific experiments can act as primary authors for the Protocol as they will be best placed to share the methodology details, but the Corresponding Author of the present research paper should be included as one of the authors. By uploading your Protocols onto protocols.io, you are enabling researchers to more readily reproduce or adapt the methodology you use, as well as increasing the visibility of your protocols and papers. You can also establish a dedicated workspace to collect your lab Protocols. Further information can be found at https://www.protocols.io/help/publish-articles.

You can use a single sign-on for all your accounts, view the status of all your manuscript submissions and reviews, access usage statistics for your published articles and download a record of your refereeing activity for the Nature Portfolio.

Nature Cell Biology encourages authors presenting evidence for cell, biological, molecular, and genetic interactions to consider

communicating these findings using Biofactoid (https://biofactoid.org/). This tool helps users share a searchable representation of interactions (e.g. binding, gene expression, post-translational modification) between genes, gene products, or chemicals. Information added to Biofactoid, with author attribution, is shared on social media and public databases, such as Pathway Commons, where it can be discovered and analyzed in the context of a large and growing corpus of knowledge.

Please feel free to contact us if you have any questions.

With kind regards,
Stelios

Dr. Stylianos Lefkopoulos
He/him/his
Senior Editor, Nature Cell Biology
Springer Nature
Heidelberger Platz 3, 14197 Berlin, Germany

E-mail: stylianos.lefkopoulos@springernature.com
Bluesky: @slefkopoulos.bsky.social
LinkedIn: www.linkedin.com/in/stylianos-lefkopoulos-81b007a0

Click here if you would like to recommend Nature Cell Biology to your librarian
http://www.nature.com/subscriptions/recommend.html#forms

** Visit the Springer Nature Editorial and Publishing website at www.springernature.com/editorial-and-publishing-jobs for more information about our career opportunities. If you have any questions please click here.**

Open Access This Peer Review File is licensed under a Creative Commons Attribution 4.0 International License, which permits use, sharing, adaptation, distribution and reproduction in any medium or format, as long as you give appropriate credit to the original author(s) and the source, provide a link to the Creative Commons license, and indicate if changes were made.
In cases where reviewers are anonymous, credit should be given to 'Anonymous Referee' and the source.
The images or other third party material in this Peer Review File are included in the article's Creative Commons license, unless indicated otherwise in a credit line to the material. If material is not included in the article's Creative Commons license and your intended use is not permitted by statutory regulation or exceeds the permitted use, you will need to obtain permission directly from the copyright holder.
To view a copy of this license, visit https://creativecommons.org/licenses/by/4.0/

NATURE CELL BIOLOGY REBUTTAL

Reviewers' Comments:

Reviewer #1 (Remarks to the Author):

This study presents a comprehensive atlas of human developing meninges using snRNAseq integrated with extensive spatial transcriptomics data from 13 donors, 16 samples spanning 5-13 post-conception weeks (pcw). The sampling includes primarily forebrain samples with regional dissections at 10 pcw, supplemented by regional spatial transcriptomics (Xenium, 389 genes) at 6 and 9.5 pcw.

Key findings include:
• Comprehensive characterization of immune, vascular, and fibroblast populations during meningeal development from primary meninx through later maturation stages
• Discovery of an inner CDH-1+ dura lineage
• Identification of ELP progenitors of B-cell lineages in the primary meninx at 5 pcw, placing them in the periphery one week earlier than previously found in fetal liver
• Demonstration of early vascular specification and subarachnoid lymphangiogenesis in fetal development
• Characterization of choroid plexus development showing early epithelial cell specification and function similar to rodents, with a potentially human-specific fibroblast type expressing TCF21 and immune-related genes (PTPRC, CD38)
• Evidence that meningioma tumors likely arise from the inner dura lineage rather than arachnoid lineages, based on spatial analyses of 10 meningioma tumors with published scRNAseq and bulk RNAseq datasets, new spatial transcriptomics of adult dura samples, gene expression similarities, and enrichment of driver gene expression in dural lineages

This study addresses a critical knowledge gap in the field by providing the first comprehensive atlas of human developing meninges at this scale and resolution. The datasets represent an important resource for the developmental neuroscience community.

The early identification of B-cell lineage progenitors at 5 pcw is an important developmental finding showing earlier distribution than liver one week later.

The discovery of the inner CDH-1+ dura lineage is highly novel and represents one of the most important findings of the paper. The reinterpretation of meningioma cellular origins—challenging the prior hypothesis of arachnoid derivation in favor of dural derivation—is significant and well-supported by multiple lines of evidence. This hypothesis is particularly well supported through: (1) spatial transcriptomics demonstrating the existence and organization of the inner dural layer, (2) gene expression similarities between tumors and inner dura, (3) enrichment of driver genes in developing dura cells, and (4) integration with existing published datasets.

The experimental design is excellent, with appropriate sampling across developmental

timepoints and integration of multiple complementary technologies (snRNAseq, spatial transcriptomics, immunohistochemistry on floating sections). The data quality is exceptional. Integration of datasets shows minimal batch correction, indicating technically sound experimental execution. The combination of snRNAseq with spatial transcriptomics (Xenium) provides robust validation of cell type identities and spatial relationships.

We thank Reviewer #1 for their very encouraging remarks, and for so clearly outlining the most important findings of our manuscript.

The paper reads well overall. However, minor presentation issues exist:
• Figure 4d insets are too small to visualize clearly and should be enlarged
• Figure S6B UMAP lacks a color key
• Sex of samples is not identified in Figure S1

We thank the reviewer for noticing, and have now fixed all three presentation issues. Figure 4d revised:

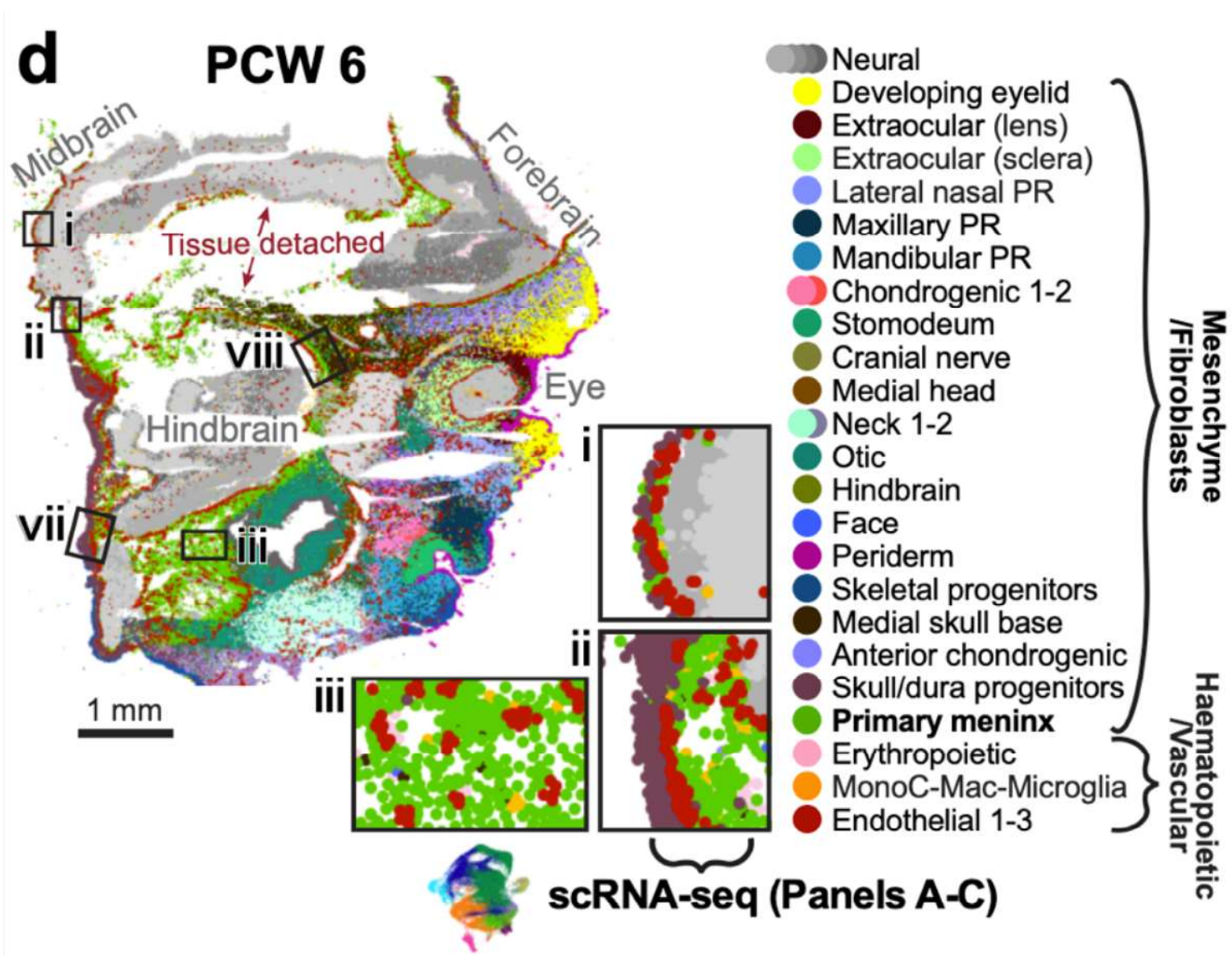

The addition of validation using immunohistochemistry on floating sections provides an excellent complementary layer of validation beyond the spatial transcriptomics.

Topic modeling was used to excellent effect to compare scRNAseq data with spatial data, demonstrating enrichment of dural versus arachnoid signatures in meningioma.

Overall Assessment
I find few issues with this paper. The analyses are comprehensive, appropriate, and well-presented. The resource is important, and new findings are impactful. The study is exceptionally well-written and accessible to a broad audience. The paper effectively communicates both the technical aspects of the multi-modal approach and the biological significance of the findings. This study represents an exceptional contribution to the field of meningeal development and provides critical insights into meningioma

biology.

We sincerely thank the reviewer for such positive and encouraging feedback on our manuscript, especially for acknowledging it as an important resource, and for noticing the efforts we had put into the experimental design, data quality, and presentation of biological findings.

Reviewer #2 (Remarks to the Author):

The authors aimed to generate a comprehensive cellular and spatial atlas of human meningeal development from 5 to 13 post-conception weeks (PCW) using high-resolution scRNA-seq and spatial transcriptomics, and connect these developmental trajectories with adult meningeal biology and meningioma tumorigenesis. They studied the emergence of pia, arachnoid, and dura fibroblast populations, the maturation of immune and vascular compartments, and linked fetal meningeal cell states to meningioma molecular signatures. Notably, the authors challenge the long-standing paradigm of arachnoid cap cell origin for meningioma and propose a new model in which tumors arise from dura-derived fibroblast states. This work provides a valuable foundation for understanding meningeal development and disease.

The work contained several strengths, including the integration of developmental and tumor datasets, the use of multi-platform data (single-cell and spatial), and the discovery of an inner dura layer with barrier-like features. The proposed dura-origin model for meningioma offers an intriguing and potentially field-shifting perspective. The dataset is extensive with >150,000 fetal cells providing a useful reference for future developmental and disease research.

We thank the reviewer for considering our manuscript as a useful resource, and for pointing out the potential impact of our findings. We also thank the reviewer for the constructive feedback below, which we have now addressed.

There are, however, several directions to improve:

1. Sample metadata and donor characteristics require further clarification. This study includes samples from 13 donors across 5–13 PCW, but additional details on donor demographics, biological variance, and potential confounders (e.g., gestational age precision, maternal health, tissue procurement factors) would enhance interpretability. As this atlas will serve as a foundational resource, transparent metadata reporting is essential for future reanalysis and integration.

We added more information about this, please see lines 932-942 in Methods under "Population characteristics", and lines 951-952. All other metadata that was available to us is reported in Supplementary Table 1.

2. Quality control, batch correction, and computational pipeline details should be more fully described. Given the multi-sample, multi-platform nature of the dataset, clear

documentation of QC thresholds, filtering criteria, and how batch effects were mitigated is critical for reproducibility. Furthermore, it would be beneficial to compare the newly generated fetal data with existing adult or murine meningeal datasets to assess biological consistency. Even though no prior study integrates fetal and adult meninges comprehensively, benchmarking against publicly available single-platform datasets would strengthen confidence in the conclusions.

We have now added more information in the text, figure legends, and Methods. Our intention is for this resource to be as easily accessible and reproducible as possible. We provide detailed information in our Methods section ("**QUANTIFICATION AND STATISTICAL ANALYSIS**") with QC documentation, filtering criteria, and computational pipeline details for all analyses. For accessibility and reproducibility, we also created an accompanying github page containing:

- raw data
- data files post-processing (as per our pipeline) both as .loom files and .h5ad, and directly browsable in CELLxGENE.
- instructions for installation of pipelines, our whole computational pipeline, and all source code (Jupyter notebooks).

Regarding batch correction, we performed Harmony batch correction on the scRNA-seq atlas as a test but concluded that the data was well integrated without it (SFig. 1e-f), and lines 117-118. This is important evidence supporting the quality of our experimental procedures, which we have developed over many years to achieve consistent, high-quality raw data.

Also see SFig. 4e and lines 364-365 for comments on fibroblast batch effects. For the spatial tumour data, to avoid supressing true biological variation the seven samples we collected were analysed separately (both clustering, LDA topic modeling, and karyotyping). It is well known that cancer single-cell data clusters strongly by tumor, and integration across patients would severely distort the data. Further details about the tumour samples below (indicated by ***).

We confirmed our main conclusions about *SLC47A1+CDH1+* cells in both murine (La Manno & Siletti et al.) and adult human (Siletti et al.) datasets (Fig. 4, SFig. 4, Fig. 5, SFig. 5, SFig. 6 & Fig. 7). We used publicly available datasets for various comparisons throughout the manuscript, i.e. from Goh et al., Jardine et al. (Fig. 2 & SFig. 2; immune cells), Braun et al. (Fig. 3; vascular development), and Choudhury et al. (Fig. 6, SFig. 6, Fig. 7 & SFig. 7; meningioma RNA-seq and scRNA-seq).

3 The inference that meningioma originates from dura fibroblast states, although compelling, requires caution and further validation. The developmental similarity between dura fibroblasts and meningioma transcriptomes is intriguing, but the claim of a dura-origin model would benefit from more explicit mechanistic evidence. Functional validation—genetic, lineage tracing, or perturbation studies—would be needed to solidify this paradigm shift. Additionally, tumor–fetal comparisons are sensitive to

batch variability and tumor heterogeneity, and further discussion of these limitations is warranted.

We agree that while our data supports a dura (even inner-dura) cell of origin, true mechanistic evidence is lacking. An obvious next step would be to generate mouse models inducing tumor formation specifically in inner dura cells. However, this is outside the scope of the current Resource paper, and we have instead revised the text to be much more careful about the claims we make. We now frame the findings as supporting a hypothesis of a dura origin of meningioma, and outline the evidence in favor but also the missing mechanistic evidence in the Discussion. Furthermore,

→ We changed the title of the manuscript (removed "a dura origin of meningioma").
→ We compared the fetal topics to tumour data from both spatial transcriptomics, scRNA-seq, and bulk RNA-seq.
→ We added independent LDA topic modelling of the tumour samples, including enriched genes/topic (now Table S8 and SFig. 8).
→ We added that our results raise (lines 537-539) "intriguing hypotheses that meningiomas 1) originating from arachnoid barrier cells later develop into dura-like cells, and/or 2) could originate from dural cells, instead of, or in addition to, the arachnoid.", and (lines 572-574) "While future mechanistic data is necessary to establish a new model, these results lend further independent support to the possibility that meningioma tumours could arise from cells of the dural lineage (Fig. 7g)."
→ We revised the Discussion.

4 Spatial validation of fibroblast and barrier-like layers could be expanded. The discovery of an inner dura layer expressing barrier genes is interesting, but appears to rely heavily on selected spatial slices. A more systematic description of spatial reproducibility across donors, sections, and developmental stages would increase confidence in this structural finding, especially since it affects the proposed disease-origin model.

Unfortunately, we did not receive intact fetal heads that were older than PCW 9 (from which we see *SLC47A1+CDH1+* cells), to spatially map the development of the arachnoid-dura barrier further. It's very difficult to get whole (intact) fetal heads, especially as fetuses get older/bigger; in Sweden currently 95% of abortions occur before PCW 10. We understand that this limits the confidence in the proposed disease-origin model, and have toned down our claims about the inner dura in the manuscript. Note that we present this observation as leading to a hypothesis of the inner dura being part of the arachnoid barrier, and discuss it extensively (lines 603-620), and it's relation to meningiomas (lines 624-644). We don't think, however, that it changes the confidence in our main finding that meningiomas are overall dura-like. In addition, in support of an inner dura layer that is *CDH1+,* our manuscript also shows:

- Spatial data from 4 human adult duras, confirming *SLC47A1+CDH1+* cells (SFig. 5f).
- *CDH1* positivity around the telencephalon, and have now added a panel to zoom into several regions (SFig. 5c).

- In the scRNA-seq data we see *SLC47A1+CDH1+* cells across donors from >PCW 9 (i.e. not limited to one spatial section). We have added a quantification of *SLC47A1+CDH1+* and *SLC22A6+CDH1+* cells across donors (SFig. 5b).
- We also confirm the existance of *SLC47A1+CDH1+* cells in the Siletti et al. dataset (human adult) and La Manno et al. dataset (mouse development).

Revised SFig. 5b-c:

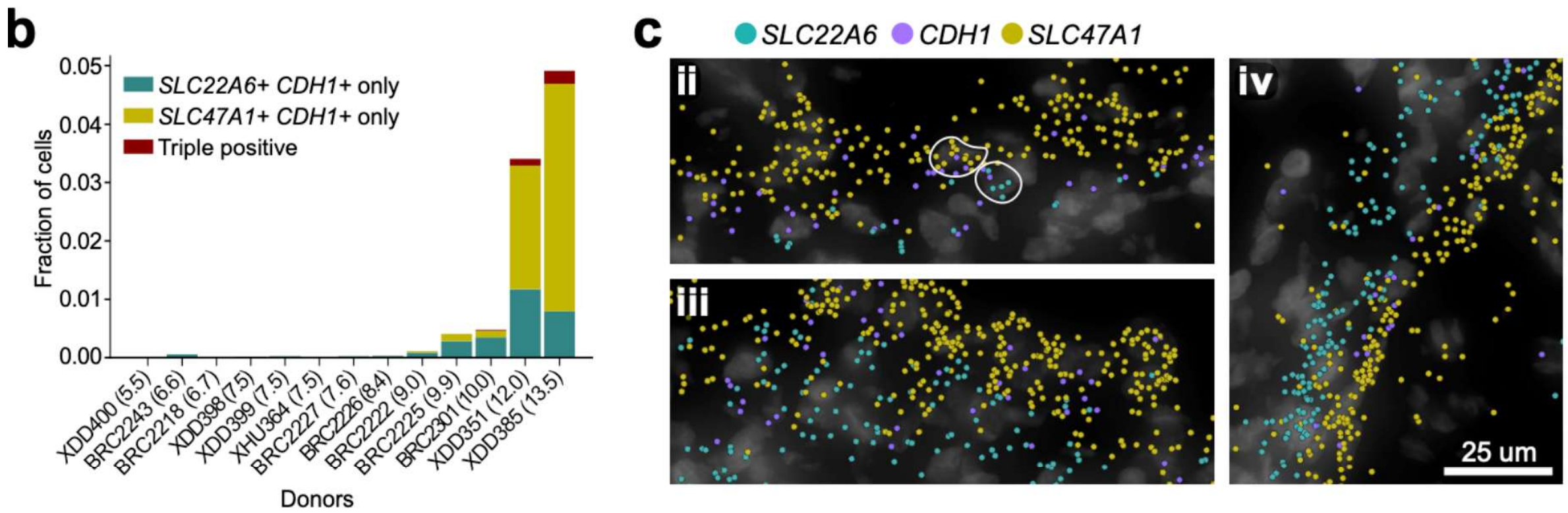

5 <u>Integration of tumor samples would benefit from more detailed explanation</u>. Menigioma bulk RNA-seq, scRNA-seq, and spatial datasets are incorporated, but it remains unclear how variability across datasets, platforms, and tumor grades was handled. Clarifying harmonization strategies and limitations would help readers assess robustness of the developmental–tumor mapping.

***We have now added more information in the text, figure legends, and Methods. To clarify, we did not integrate the different data modalities. For example, the LDA topic transfer was performed on our fetal scRNA-seq data, and transferred to A) the spatial data (each sample independently), B) scRNA-seq of meningioma (pooled but not harmonized), and C) bulk RNA data (185 samples, presented per methylation grouping as per Choudhury et al.).

To summarise tumour samples:
→ our spatial: n= 7 (1 grade I, 6 grade III)
→ bulk RNA-seq: n= 185 (across grades I-III)
→ scRNA-seq: n= 6 (2 grade I, 3 grade II, 1 grade III)

Since we generated spatial data from meningiomas, we decided to focus on grade III hypermitotic meningiomas (6 out of 7 samples collected). However, bulk RNA-seq from Choudhury et al. was generated from n=185 meningiomas across grades I-III, and scRNA-seq from Choudhury et al. was generated from n=6 meningiomas also across grades I-III. We therefore conclude that our main observation that cell types in meningioma tumours are mostly dura-like is robust, as it applies to all tumour grades and data modalities we performed our analyses on. Please see the Discussion for comments on our limitations.

Below are the comments from an early career researcher co-reviewed this paper:

The study addresses an important biological question, but several methodological issues require further clarification or additional analyses to support the authors conclusions.

• The conclusion that "Over time, endothelial cells transitioned from the primary vascular plexus at PCW 5, to arterial and venous capillaries around PCW 7-9, and larger arterioles and venous lymphatic-like cells at PCW 12-13" is not adequately supported by the current analysis. Trajectory or pseudotime analysis would be more appropriate for demonstrating developmental transitions. Please consider pseudotime analysis. Additionally, Fig. 3E appears to show cell composition rather than transitional changes.

We thank the reviewer for noticing and for the suggestion. After implementing scVelo analysis, we have rephrased that paragraph - please see lines 226-230.

Revised Fig. 3b with added RNA Velocities:

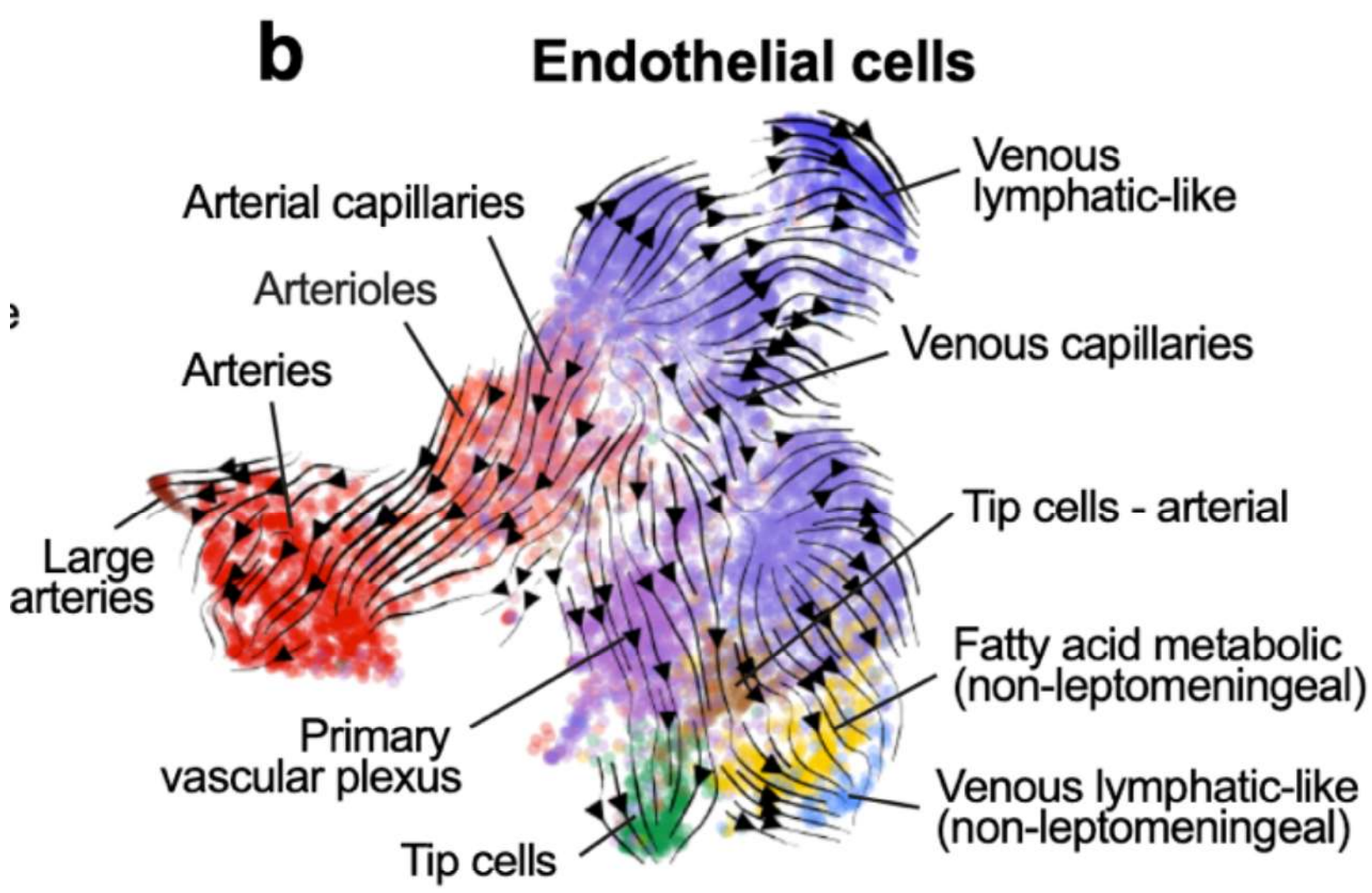

• The observation that the forebrain lacks venous capillaries at PCW13 may be due to limited sample size. This finding should be presented cautiously unless validated in a larger cohort.

Absolutely. Our observation was that there were no venous capillaries [that whole developmental period] even at PCW 13 – but we have rephrased it now to make it clear that we weren't only referring to PCW 13, but PCW 5-13. Please see lines 230-232.

• Lines 403-405: Please specify in the Methods how the signature scores were computed and provide the list of the 50 enriched genes used to build these signatures. It is also unclear how these enriched genes were determined, please clarify that.

We provided descriptions under Methods "Fibroblast layer-maturation score", and in the jupyter notebook "Fig4_SFig4_and_layer_score_Fig7.ipynb" (available on our accompanying github page), containing the full pipeline including calculation of the top 50 genes per layer. We have also added a more detailed explanation in the Methods section now and hope that it will be clearer to the readers. For the list of enriched and filtered genes used for the analysis see SFig. 4f.

• Given the distinct biological differences between outer dura, inner dura, the meningeal barrier, and the skull/dura progenitor population as described in the section "Identification of an inner and outer dura layer", a <u>cell-cell communication analysis would be highly informative</u>. A <u>ligand-receptor analysis</u> would identify the signaling pathways that drive differentiation of outer versus inner dura and pattern formation. Such analysis would offer mechanistic insight into how these layers interact and are specified during PCW5-6.

We had performed such analysis but decided not to include it because of the many caveats. Computational analyses of receptor-ligand pairs can only generate hypotheses about possible interactions, but does not show the existence of true functional interactions. There are numerous reasons why a possible interaction may not be actual (for example, the ligand concentration may not reach the Kd of receptor binding, the receptor may not be sorted to the cell surface, the downstream signalling pathways may not be present, or may be inactive, the transmitting and receiving cells may not be in proximity, or may be separated by a membrane, the ligand may be sequestered by extracellular matrix and so an and so forth). As a consequence, such analyses often generate hundreds of weakly supported hypotheses. Below is a summary of what we found when testing just one possible interaction, that between inner dura and arachnoid barrier cells:

CellPhoneDB analysis showing inferred signalling interactions (Y-axis) between inner dura and arachnoid barrier cells (X-axis) in <u>one</u> L-R direction. AB = arachnoid barrier, iD = inner dura.

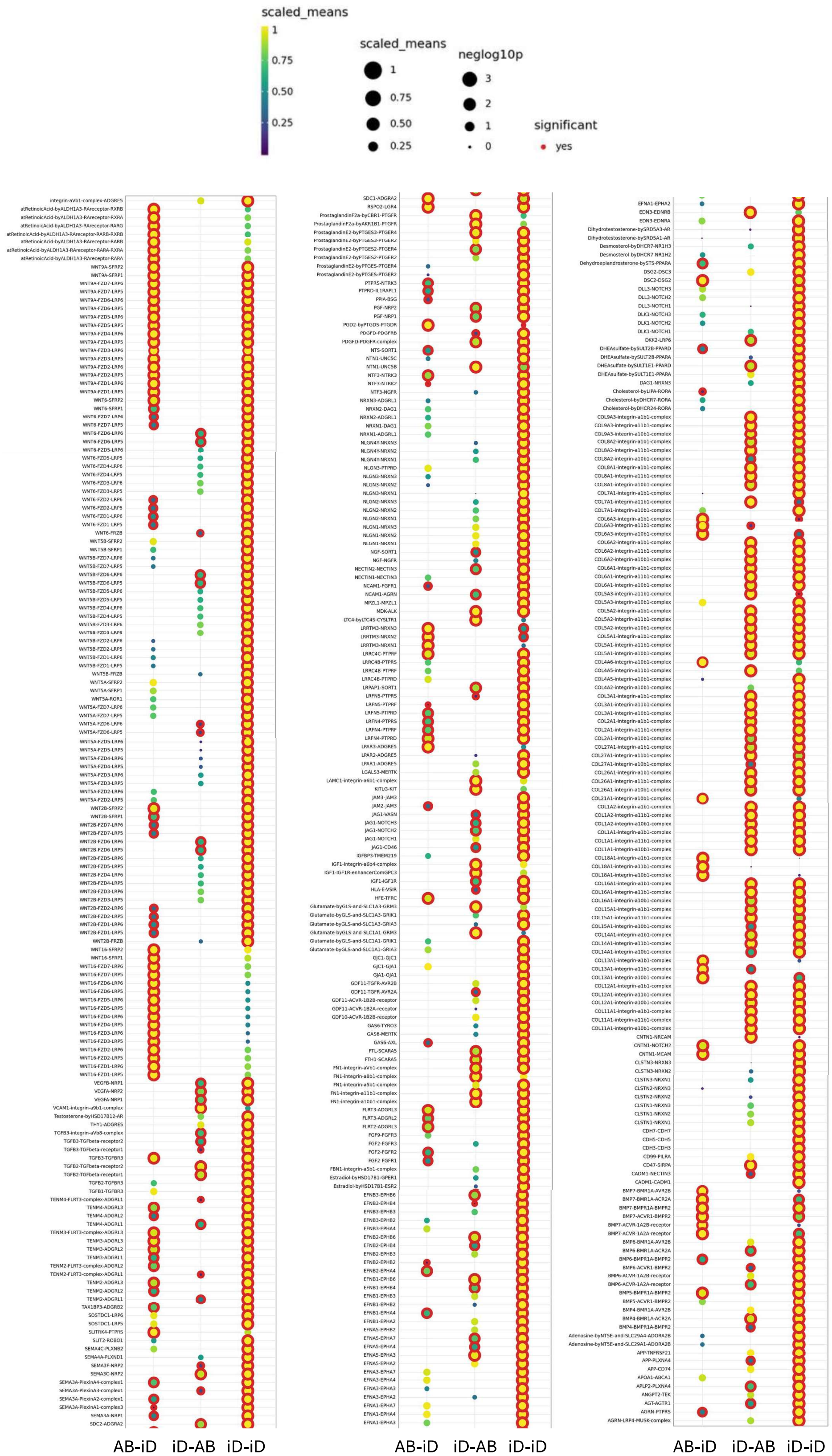

• Lines 436-439: The multi-layer fibroblasts organizations (meningeal barrier, inner dura sheet, periosteal layer, periderm) suggests the presence of distinct fibroblasts niches. A spatial niche detection or neighborhood analysis would substantially strengthen the interpretation of fibroblast layer formation.

We have now implemented a Banksy spatial domain analysis, which for example supported our conclusions about (1) that osteogenic/dura progenitors together with the primary meninx seem to form the entire meninges, and (2) the relatedness between the outer dura and other osteogenic tissues such as the developing skull. See panel additions in SFig. 4h-i (also below), and proportions of cell types in each domain in Table S9.

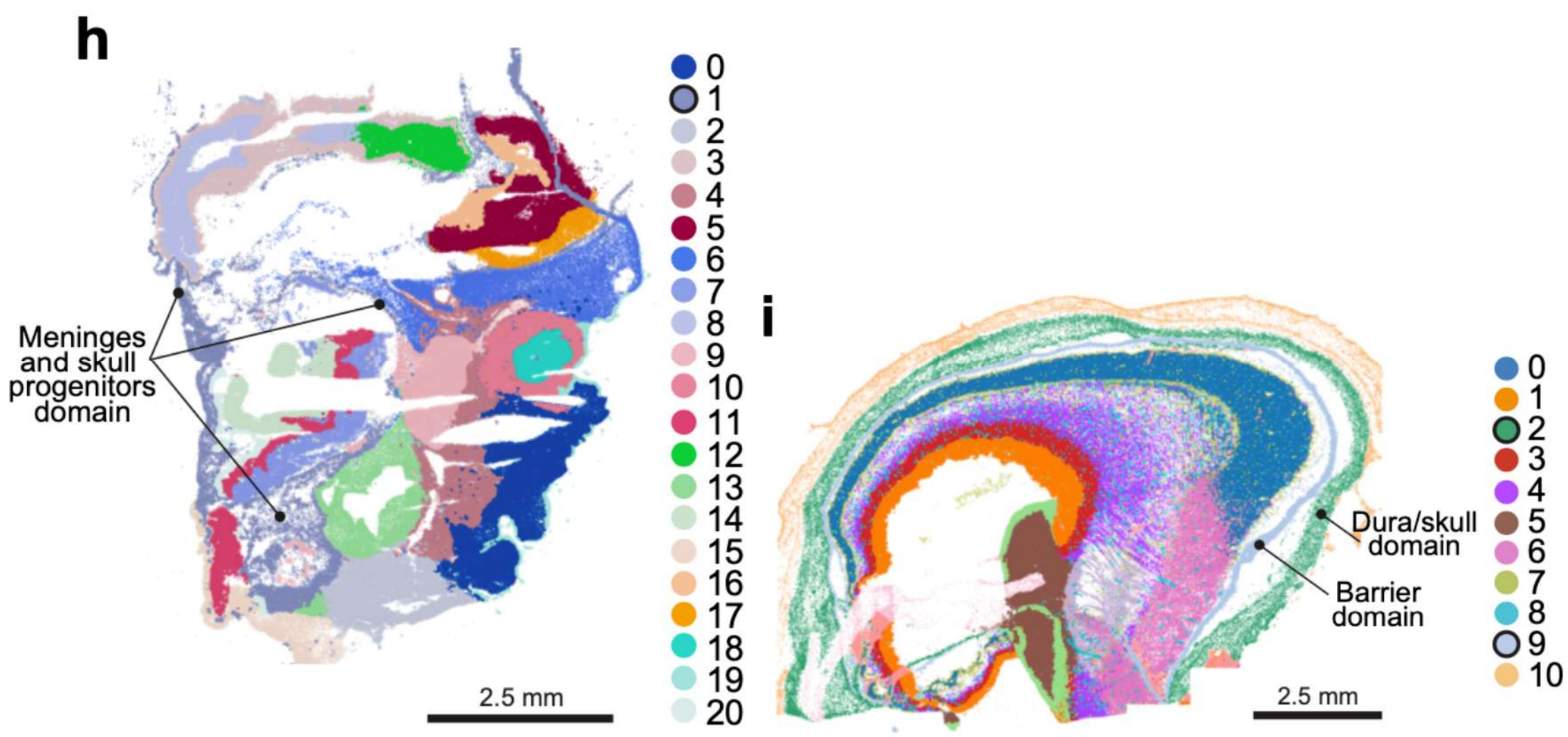

• Lines 532-538, The LDA analysis identifies 35 gene modules (topics), and several are described as corresponding to pia, arachnoid, PTGDS+ precursors, inner dura, and dura. For interpretability it would be very helpful to report the top genes contributing to each topic (e.g., top 10–20 genes per module) or atleast the topics reported in Fig. 6A and to provide annotation linking these top genes to cell types or anatomical locations where these modules show higher scores. This would allow readers to better understand which transcriptional programs each topic represents, and what genes are the top contributors to these topics.

We have provided this information, please see Supplementary Table 7 for top genes/topic together with topic annotations.

• Transferring scRNA-seq LDA topics directly onto the meningioma spatial transcriptomic data raises methodological concerns. LDA topics are inherently dataset-specific, reflecting the latent gene program structure of the dataset on which they were trained. Given the substantial biological and technical differences between the scRNA-seq data and the spatial data, direct topic transfer may not faithfully capture the latent structure of the tumor samples. A more robust analysis would be to perform

LDA independently on the spatial transcriptomics samples, characterize the tumor-specific topics, and then evaluate their correspondence to the scRNA-seq programs.

We thank the reviewer for suggesting this additional analysis. We performed LDA topic modelling on our tumour spatial data independently, and identified genes that define those topics. As a validation we then transferred the tumour topics to fetal PCW 9.5 spatial data, to see if the tumour topics resemble any anatomical structures (like meningeal layers). Please see the added results in SFig. 8, Table S8, and in the results lines 531-539. We originally also transferred the LDA topics from fetal scRNA-seq to meningioma scRNA-seq (SFig. 7) and bulk RNA-seq (Fig. 6, SFig. 7), containing the whole transcriptome, and still saw the same results; that dura-topics dominate.

New **SFig. 8** showing LDA topic modeling on tumours, and transferred to fetal:

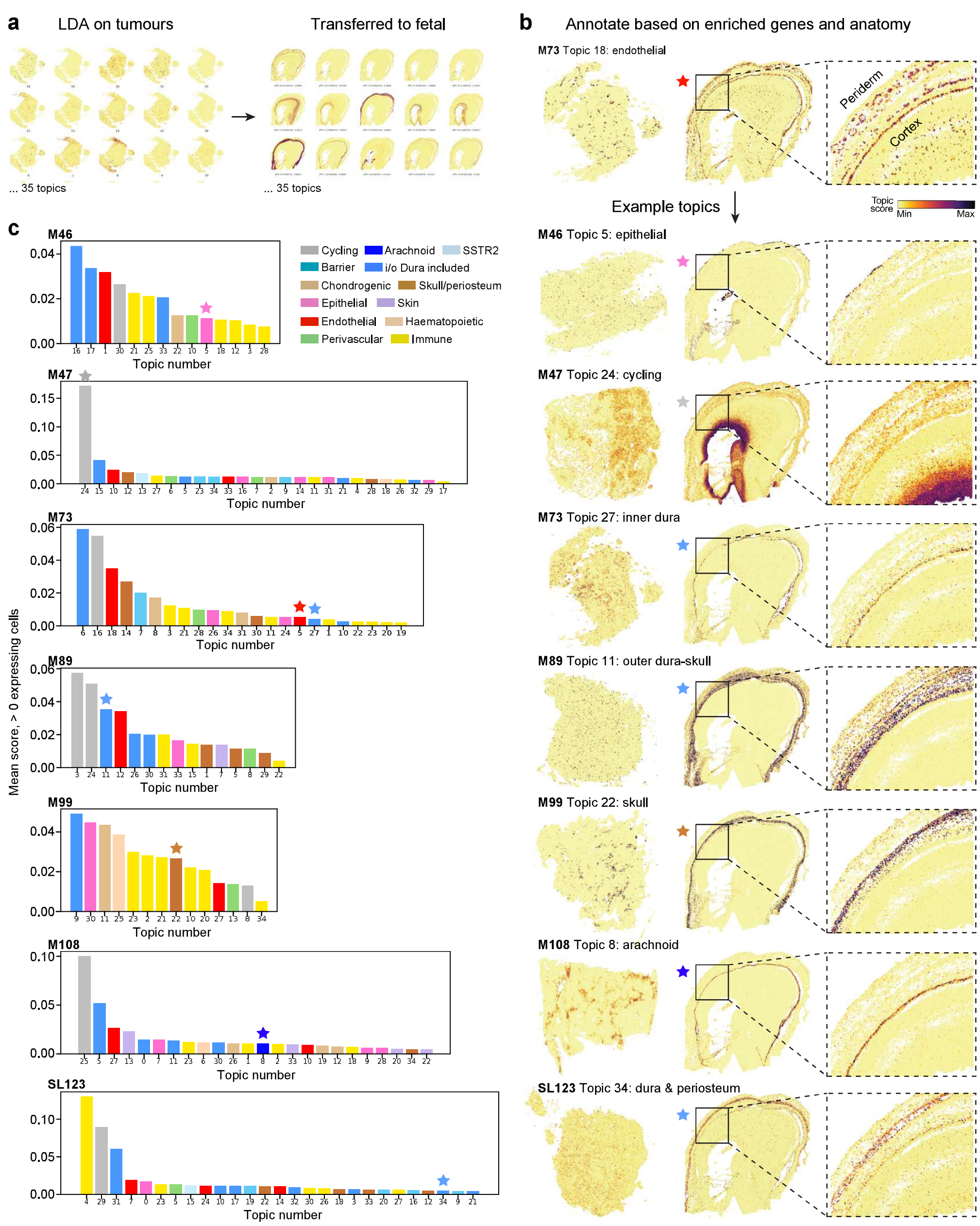

• Lines 582-592: The description of spatially structured vs uniformly mixed tumors, as well as the presence of nested dura-like, inner-dura-like, periosteal-like, chondrogenic, and cranial-like regions (Fig. 6H), strongly suggests the existence of distinct cellular neighborhoods or niches. It would be highly informative to <u>perform a cellular neighborhood or niche detection analysis to provide a better understanding of the spatial organization of meningiomas</u>, vascular-immune microenvironments, dura-like vs cranial-like spatial architectures and would support many of the spatial claims made in the manuscript.

We have now implemented a Banksy niche detection analysis of each tumour to strengthen claims made in the manuscript. See Table S9 and new SFig. 9 (also below):

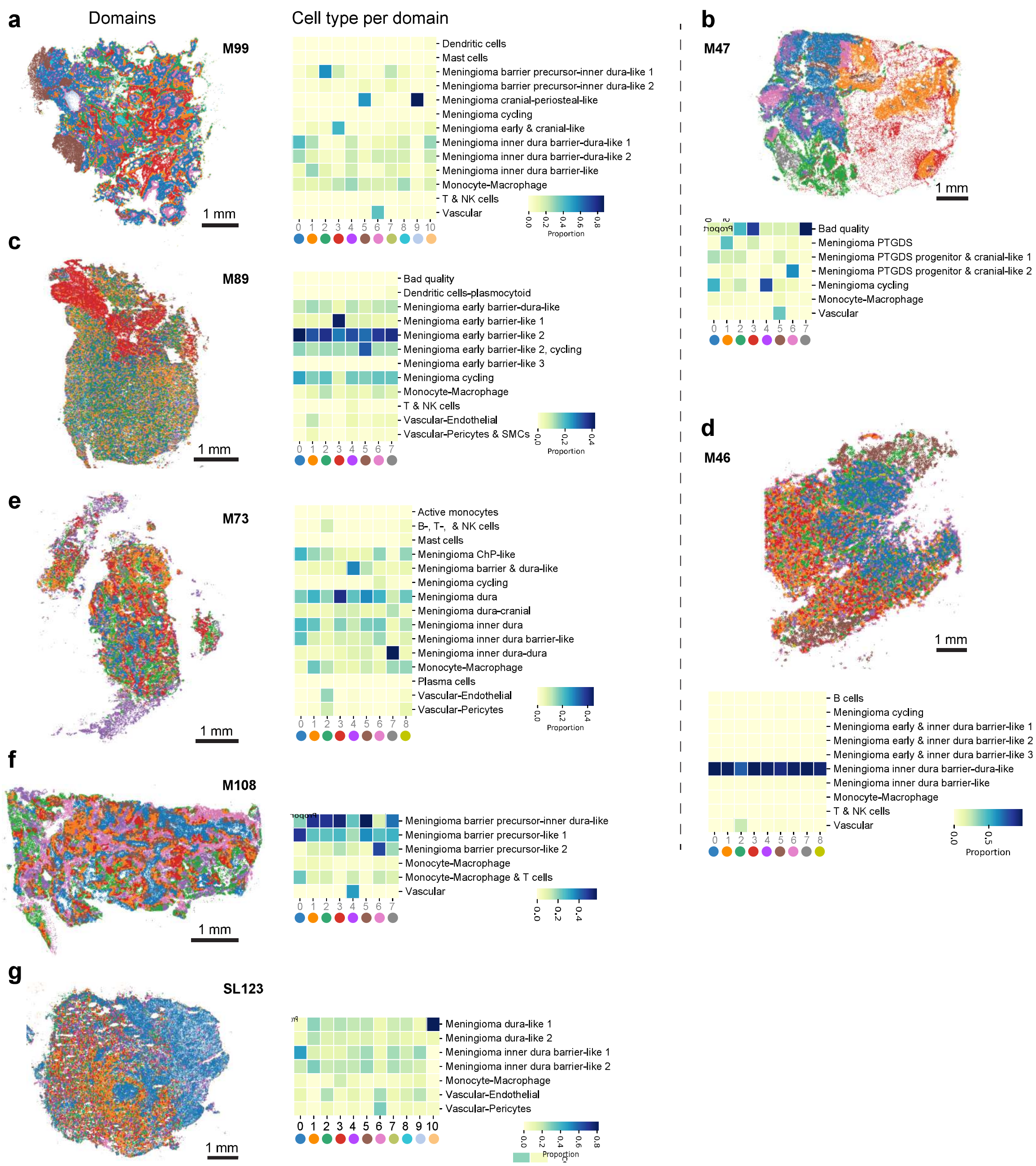

• The Methods section <u>does not explain why K=35 topics were selected for LDA</u>. Please justify the choice of topic number.
• Some LDA topics were described as not biologically meaningful. Did the authors consider using a smaller K (topic number) to obtain fewer but more coherent and interpretable topics?

We have now added an explanation behind our rationale of using k=35 in the Methods section. Since we had 55 fibroblast clusters that we could annotate to 29 distinct cell types as a reference point – we generated a range of 25, 30, and 35 topics. All of these contained non-cell type specific topics (for example 2 topics for cycling cells, and 1 topic for chromosome Y-linked genes). We made our decision of k=35 based on the resolution of meningeal topics, which were the most biologically meaningful in this study. In topic modelling, there is no objectively correct number of topics. Instead, there is always the trade-off where asking for more topics also generates more topics that are not relevant or easily interpretable (such as related to weak spatial gradients, or subtle signalling events, or nuanced experimental artefacts). Here, the key deciding factor was that with 35 topics the dura separated into two topics, representing more clearly the inner dura, and dura. With 25 and 30 topics there was only one dura topic. Other relevant topics, such as those active in arachnoid and barrier cells, did not split further. Therefore, we didn't generate more than k=35 either since it stopped showing meaningful new topics (see Table below). Since any number of topics can be generated, we believe we still kept a relatively conservative approach.

Table showing topics active in meningeal layers when generating 25, 30 and 35 topics:

	Topic description and matching topic number			
Total	Dura	Barrier	Arachnoid Precursors/PTGDS	Arachnoid
25 Topics	22	12	12	3
30 Topics	19	16	2	28
35 Topics	17, 30	20	28	23

Reviewer #3 (Remarks to the Author):

I co-reviewed this manuscript with one of the reviewers who provided the listed reports. This is part of the Nature Cell Biology initiative to facilitate training in peer review and to provide appropriate recognition for Early Career Researchers who co-review manuscripts.

We thank Reviewer #3 for their constructive feedback, and hope that we addressed them all to an extent that strengthens the manuscript for NCB.

Reviewer #4 (Remarks to the Author):

The authors present an impressive and well-curated dataset of human meninges across development, adult dura, and meningiomas, profiled using both scRNA-seq and

spatial transcriptomics. Major cellular compartments are clearly clustered and annotated, and the developmental time series is leveraged effectively to highlight changes in fibroblast, vascular, and immune populations over time. Overall, this represents a valuable resource, and the authors should be commended for putting together such a comprehensive dataset.

We thank the reviewer for considering our manuscript as a useful curated resource, and for taking an interest in our findings. We also thank the reviewer for the constructive feedback below, which we have now addressed and believe significantly strengthens the manuscript.

Major comments

1. It would be helpful if the authors could briefly comment on how batch effects were assessed and addressed. Because all cells were integrated into a single dataset for top-level cytograph clustering, a quantitative summary of donor and developmental-stage composition (e.g., per-cluster donor/stage fractions or per-sample cell-type compositions) would strengthen the manuscript. Although the UMAPs do not show obvious batch-driven artifacts, providing such summaries would increase confidence that major clusters are robust and shared across donors and developmental stages, further enhancing the utility of the atlas as a resource.

We thank the reviewer for this suggestion, and added a barchart showing the percentage of major cell types per donor (see Fig. 1f). We performed Harmony batch correction for donors on the scRNA-seq atlas (SFig. 1e-f), and on pooled datasets of PCW 5-6, PCW 9-10, and PCW 12-13 samples (SFig. 4e), but concluded that the data was well integrated without it. Therefore, we proceeded without batch correction to avoid supressing true biological variability. To increase confidence of cell type annotations we annotated the clusters on 4 different levels of confidence/robustness (Fig. 1e, Table S2).

Revised Fig. 1f

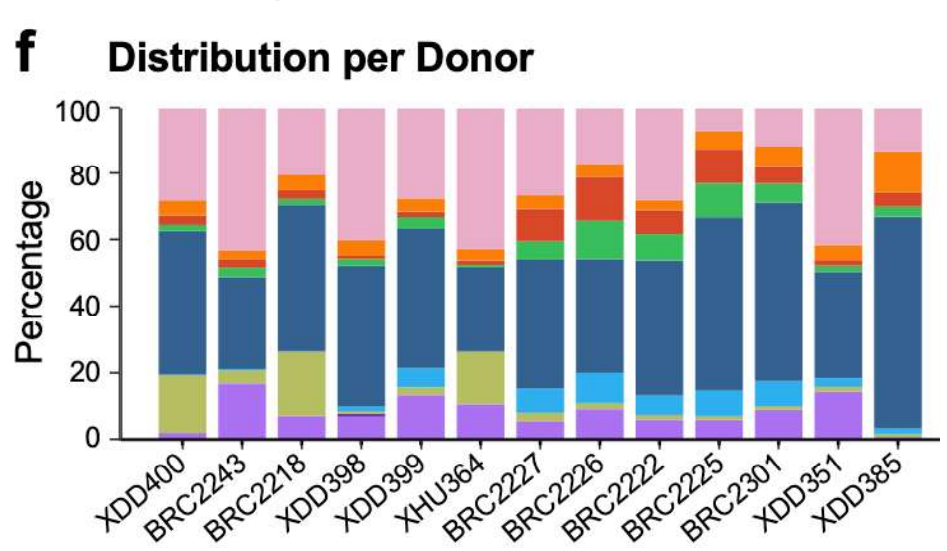

SFig. 1f

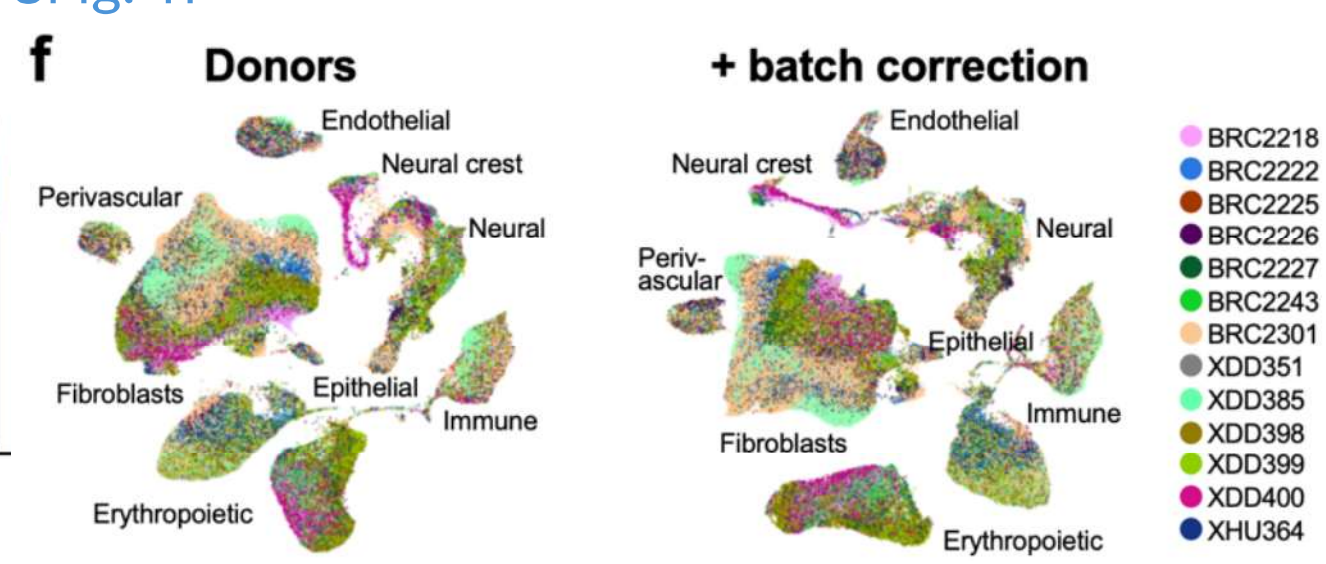

2. In Figure 6, panel F provides a helpful visual summary of mean topic scores across tumors, showing a clear separation between arachnoid/pia vs. inner-dura/dura/barrier. However, the evidence remains largely descriptive. Given that this topic analysis underpins a central claim regarding the putative cell of origin, adding a statistical comparison at the individual sample level would strengthen the claim (for example, per-tumor mean topic weights for arachnoid versus inner dura/barrier topics, with effect sizes and confidence intervals).

We have now used a Mann-Whitney U-test with FDR multiple correction <5%, and revised Figure 6f:

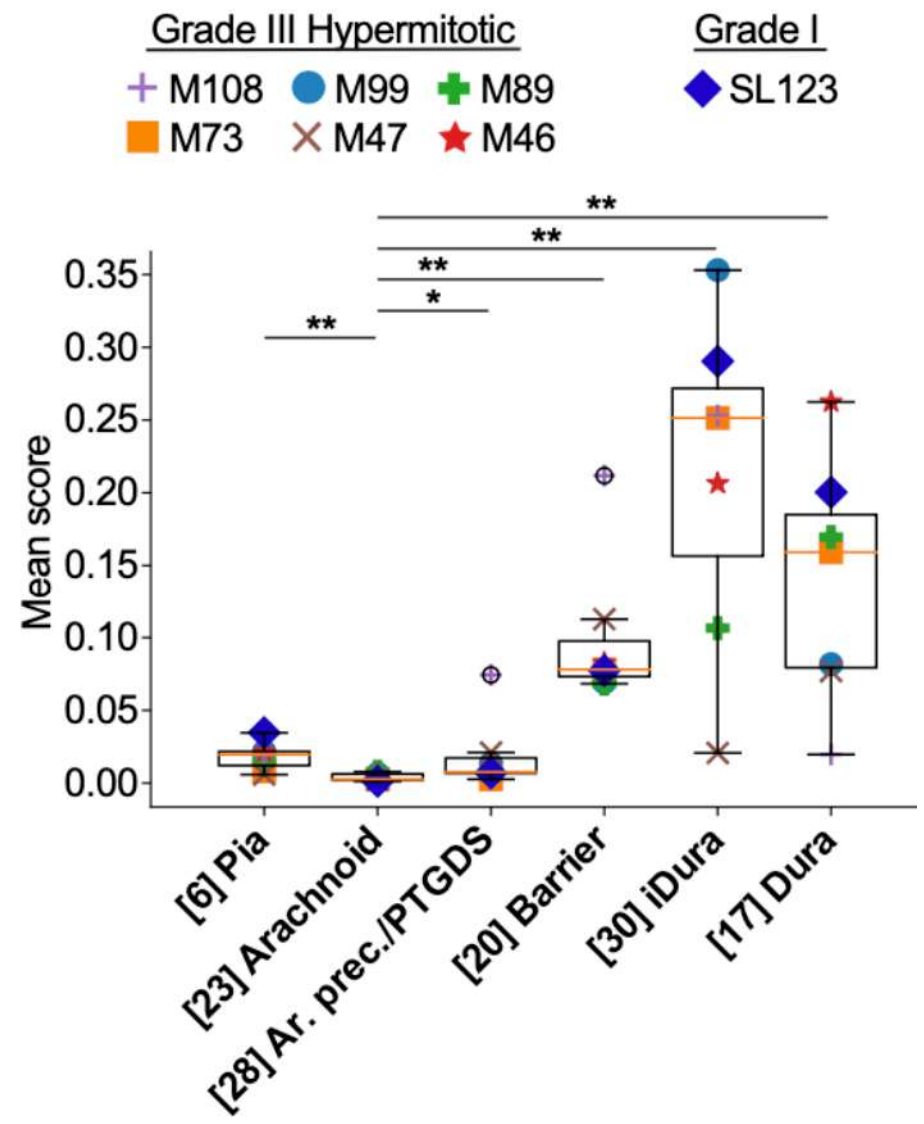

3. For the Xenium datasets, the decision to retain only transcripts within segmented nuclei seems reasonable to minimize segmentation artifacts, but it may discard a substantial fraction of cytoplasmic signal. A short comment on how this choice might influence sensitivity for particular cell types, or whether any systematic bias across lineages was observed, would be informative for interpreting the spatial data.

This is a fair comment. Briefly, we decided to segment nuclei because the cells were densely packed in fetal tissues, so this would minimize "contamination" of molecules from neighbouring cells (especially layer-building fibroblasts). Nuclear segmentation can lead to filtering out too many cells, but since we had a good number of transcripts per nuclei this did not happen. However, it is important to note that the signal is not only nuclear, since we are capturing 3D structures in 2D – i.e. cytoplasmic RNA above and below the nuclei are also captured.

All major cell types that were found in the scRNA-seq data were also found in the Xenium datasets (as illustrated in all figures). Therefore, we don't see any obvious lineage biases. For clustering we focused on the fibroblasts, and can note that to have individual clusters of all immune cell types that we manually validated in the Xenium dataset further clustering is necessary. A few references of the general caveats of in situ analysis, including Xenium and nuclear segmentation biases, have been added to the Methods section.

4. As the meninges mature as a continuum of cell states, it would be informative to apply trajectory analysis, such as pseudotime and RNA velocity, to identify transcriptional factors or master regulators driving the transitions between developmental or lineage-specific states.

We did this on a subset of fibroblasts only containing those of the meningeal lineages (removed choroid plexus fibroblasts etc). We can see that 1) the pia, arachnoid, and dura are all endpoints, 2) the cells that we labelled as precursors behave as precursors, 3) potentially what we annotated as the earliest chondrogenic [progenitors] give rise to the primary meninx, and osteogenic lineages, and 4) what we annotated as arachnoid precursors potentially can give rise to the pia and inner dura.

However, our data does not show a typical linear or Y-shaped lineage trajectory (as seen in for example neurogenesis or haematopoieses), consistent with our observations in Figure 4. Therefore, we think the scVelo results on these fibroblasts are too complex to draw definitive conclusions about lineages and master regulators. While it would be very interesting to answer the questions that Reviewer #4 raises, since it is not in the scope of this manuscript to prove inferred lineages, we did not integrate this panel into the manuscript.

scVelo analysis on PCW 5-13 meningeal fibroblasts:

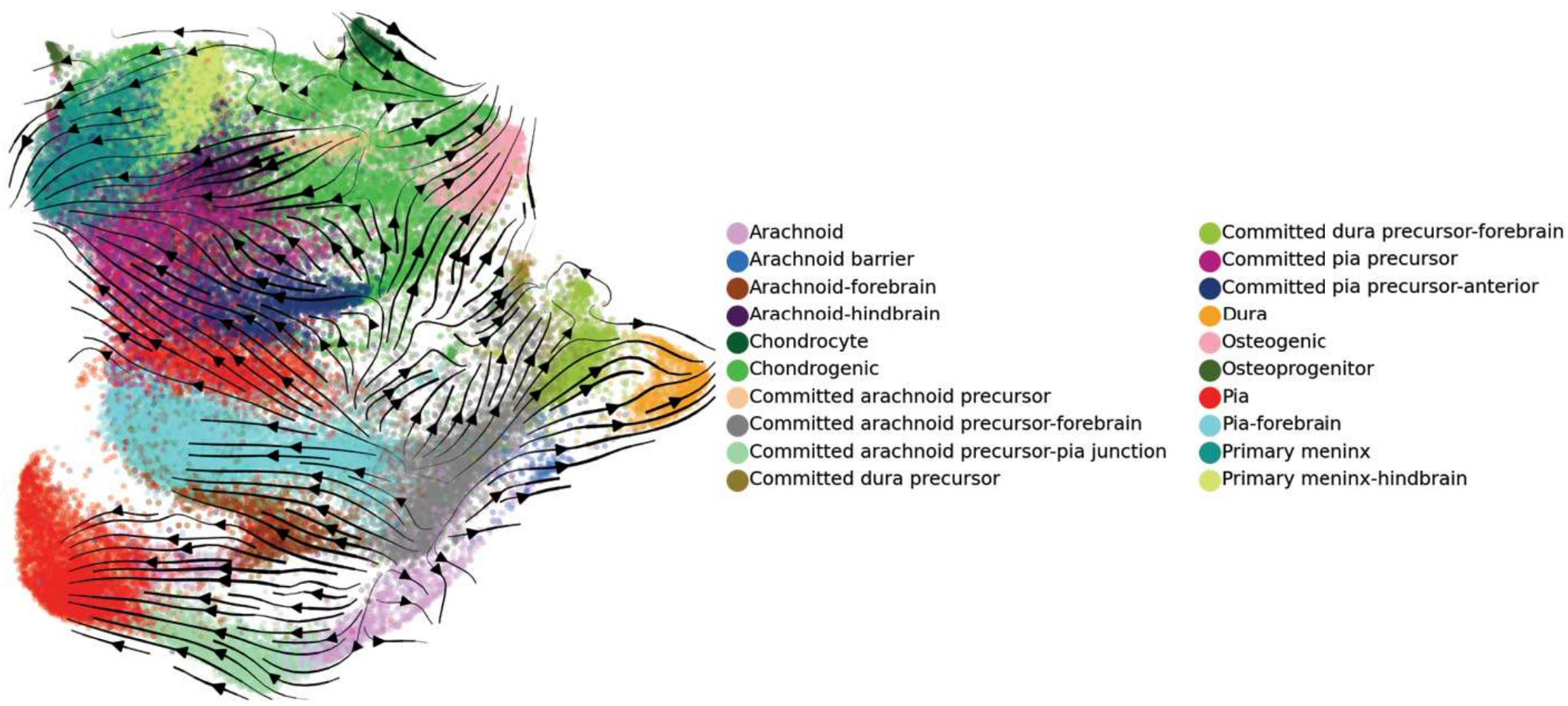

5. Previous studies have commonly suggested the arachnoid as the putative cell of origin for meningioma, whereas the current study identified dura-related populations as dominant among tumor cells. It would be helpful if the authors could briefly summarize the historical rationale behind the arachnoid-origin hypothesis and clarify how their findings reshape this understanding.

We have described the historical rationale of an arachnoid origin in the introduction (lines 68-75), and put our results in this context both in the results section (lines 420-432) and discussion (lines 638-641 & 653-670).

6. RNA diffusion or "smearing" is a known and common issue in spatial transcriptomics and can lead to apparent expression in neighboring cells. Is it possible that CDH1 expression in "inner dura" cells reflects diffusion from adjacent arachnoid barrier cells rather than authentic endogenous expression? A short discussion or control analysis would help clarify the observed signal is intrinsic or an artifact.

We agree that this is a common issue, and a valid concern. However,

1) we performed nuclear segmentation of the Xenium spatial data to mitigate this effect, and the “inner dura” appear as a separate cluster (Fig. 4e & j),

2) in the spatial data we see *SLC22A6+CDH1+* cells (arachnoid barrier precursors) and *SLC47A1+CDH1+* cells (inner dura) – but for example neighbouring pial *LAMC3+* cells or endothelial cells that were also *CDH1+* were not observed (other than a couple of cells here and there probably reflecting the extent of diffusion, rather than as a consistent band of *CDH1+* cells as with the arachnoid barrier/inner dura cells). I.e. if spread of *CDH1* molecules was due to diffusion/smearing, we believe this artifact would be observed in other cell types neighbouring the arachnoid too.
3) we observe *SLC47A1+CDH1+* cells quite evenly around the whole sagittal section of the telencephalon.

In SFig. 5, spatial transcriptomics at PCW 9.5 around the head, and zoomed in:

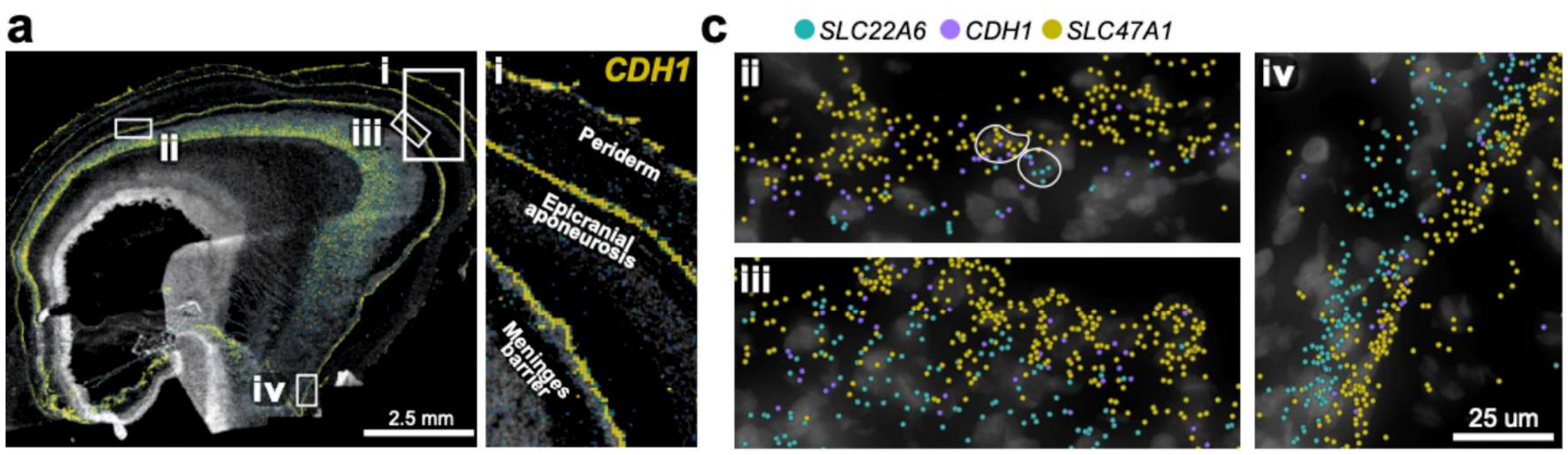

4) we see *SLC22A6+CDH1+* and *SLC47A1+CDH1+* cells in scRNA-seq data too, across mouse embryonic scRNA-seq (La Manno et al.), human developmental scRNA-seq (this manuscript) and human adult single <u>nucleus</u> RNA-seq data (Siletti et al.). scRNA-seq and snRNA-seq data does not suffer the same issues concerning contamination of signal by direct neighbours, so this is a strong corroboration.

In Fig. 5, scRNA-seq data as above:

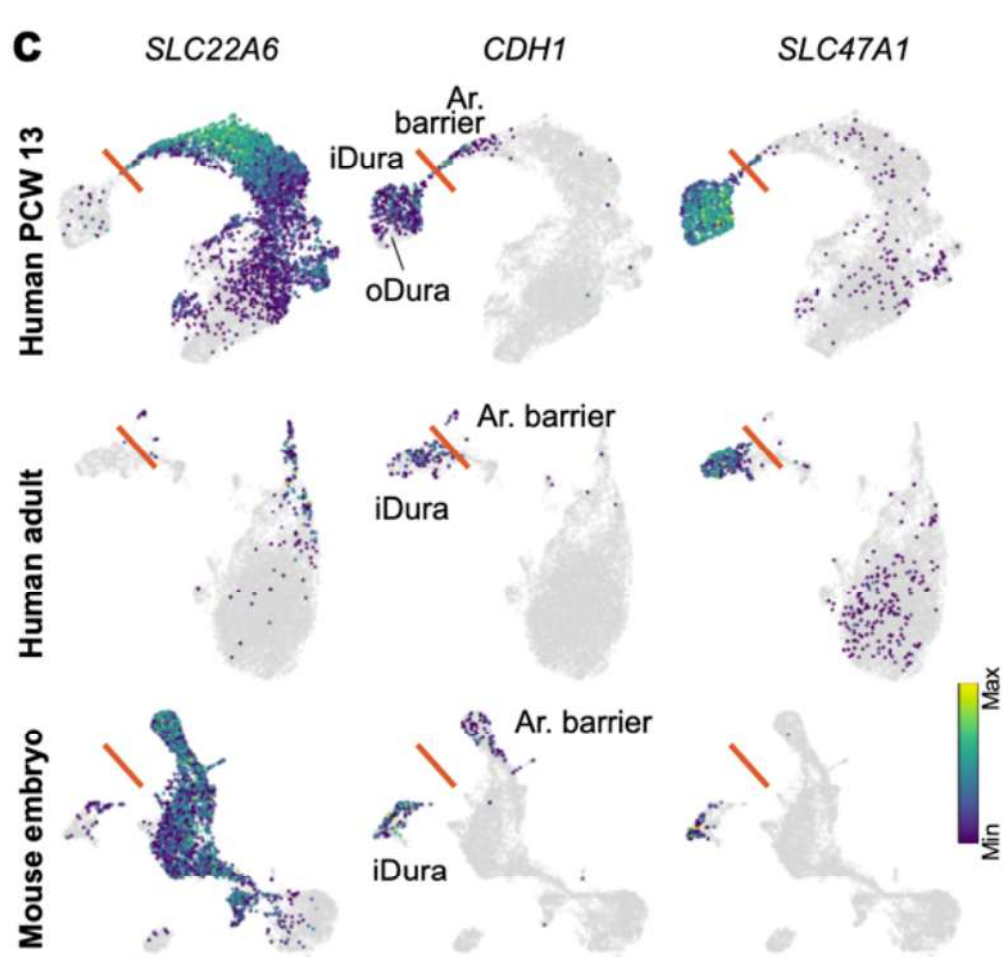

(In SFig. 5, human adult dura spatial data:)

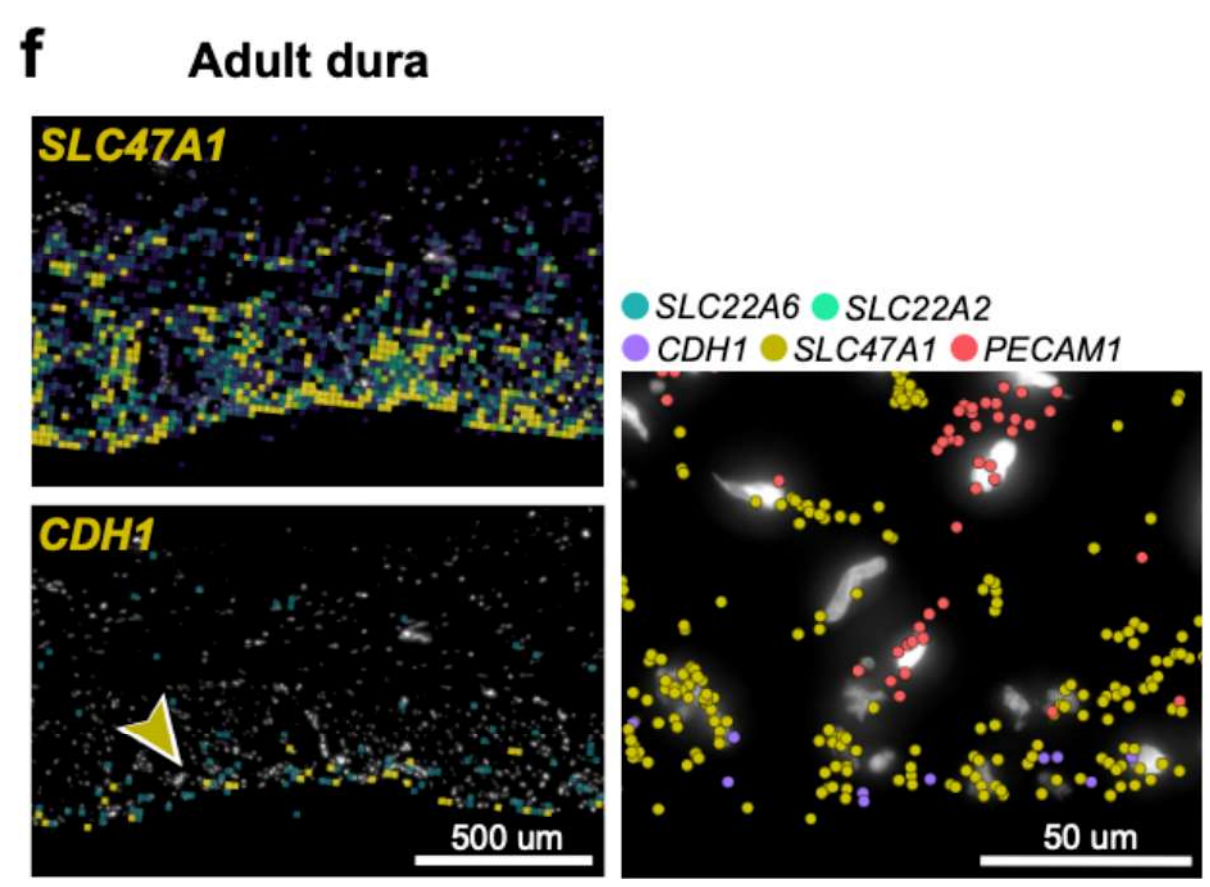

7. Some of the 20 most frequently mutated genes such as NF1 and NF2 are tumor suppressors, and their transcript levels are often depleted in meningiomas, especially in NF-loss tumors. Therefore, it is somewhat surprising to see them included in this analysis and highly expressed in iDura populations. Instead of focusing on these tumor suppressors, it may be more informative to highlight proto-oncogenes whose elevated expression is commonly observed in tumor cells and may better reflect lineage- or state-specific oncogenic programs.

The genes that are frequently mutated in a cancer type are expected to be expressed in the cell type of origin, before transformation to cancer. This is especially true for tumor suppressor genes, whose loss is what drives tumorigenesis. If a tumor suppressor gene were not expressed in the cell of origin, its loss would have no effect. Ergo, it has to be expressed in the (normal) cell type where the mutation happens to cause tumor formation. Similarly, an amplified proto-oncogene must be expressed in the cell of origin, for its amplification to drive tumorigenesis, since multiple copies of a gene that is not expressed will have no effect. And again similarly, an activating mutation in a proto-oncogene protein will have no effect if the proto-oncogene is not expressed. It follows that the patterns of frequently mutated genes should match (in some way) the patterns of genes expressed in the cell of origin.

There will of course be some exceptions. For example, a common driver in many cancers, *TERT* (telomerase) is not normally expressed in any somatic cells. Activating mutations in the promoter cause it to be expressed *de novo*, and this helps drive tumor formation by removing the Hayflick limit to cell division. We have now redone the analysis omitting *TERT*, with unchanged results (unsurprisingly, since the gene is not expressed in any normal somatic cells).

Since the reviewer requested, we also ran the analysis without tumour supressors (*NF2, NF1, TSC2, APC, LRP1B, LZTR1, KMT2C, KDM6A, SMARCA4, PRKAR1A*), and with only clear proto-oncogenes in meningioma (*AKT1, SMO, EGFR, MET, ROS1, TERT*) +/- likely oncogenes (*KLF4, PREX2, NOTCH1*). See their expression in meningeal cell types:

1. Clear + likely oncogenes

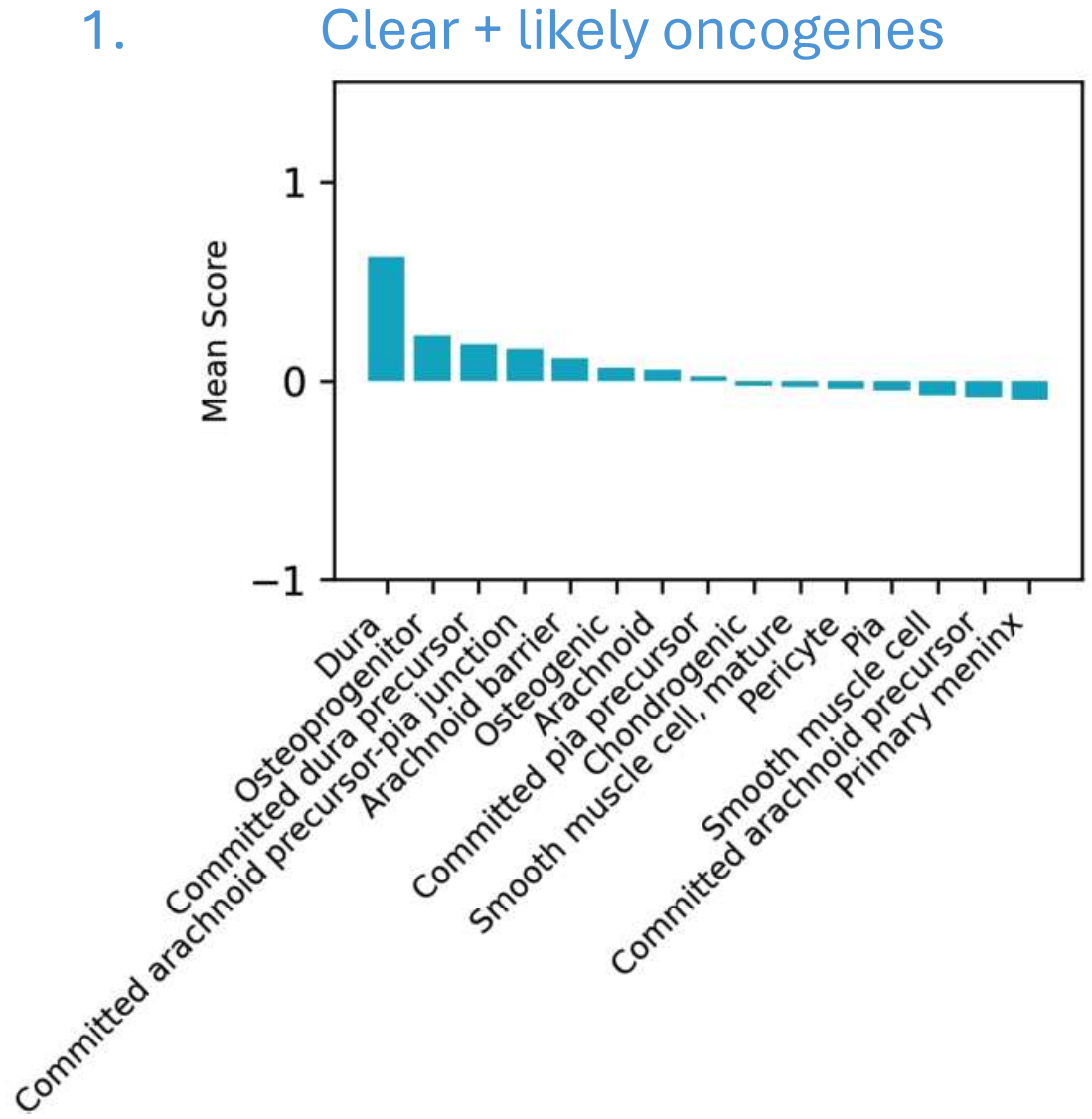

2. Only clear oncogenes

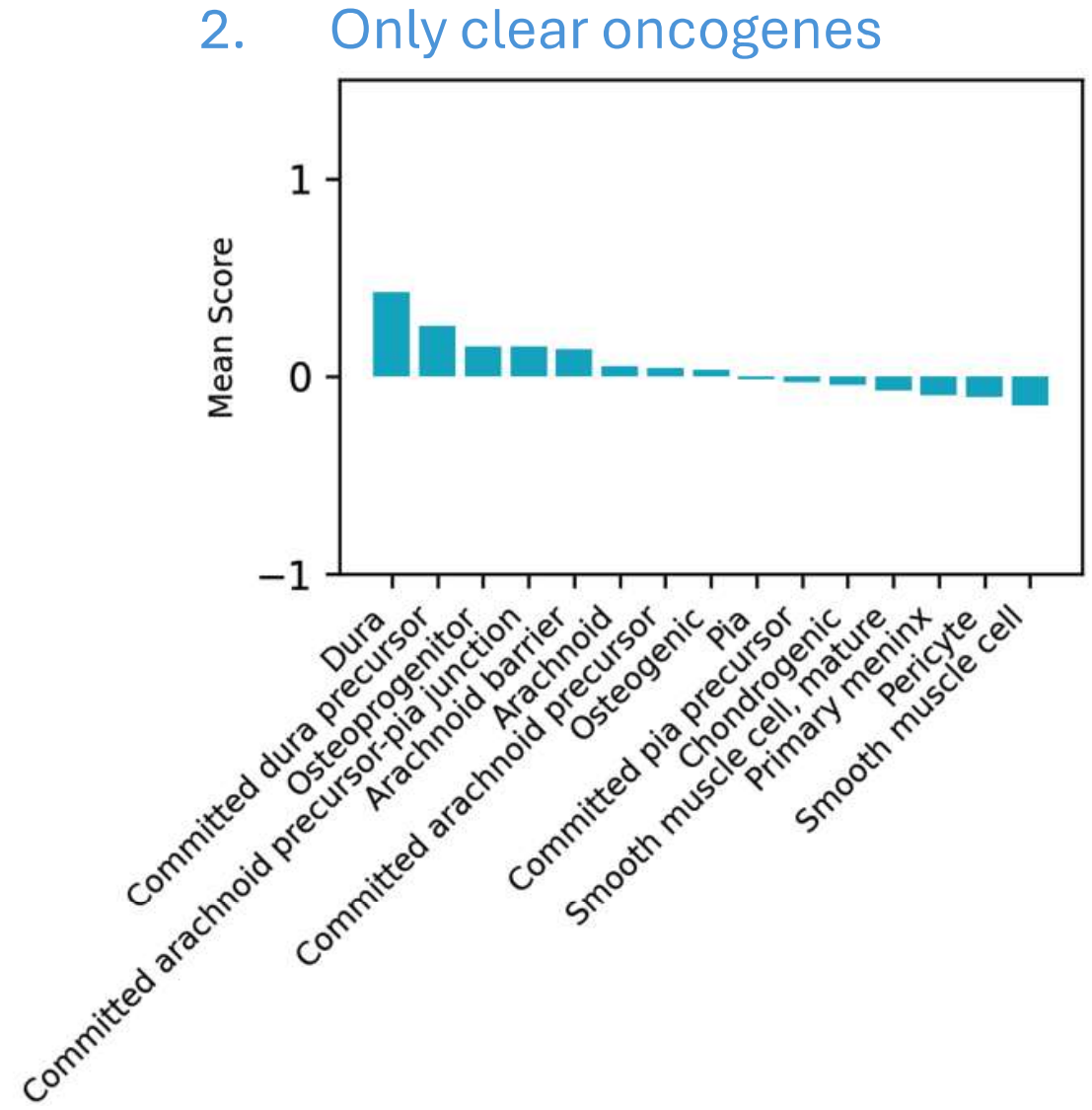

Supplement: Supplementary file 2 [file 41556_2026_2074_MOESM2_ESM.pdf]
